# Analyzing Grid-Based Direct Quantum Molecular Dynamics Using Non-Linear Dimensionality Reduction

**DOI:** 10.3390/molecules26247418

**Published:** 2021-12-07

**Authors:** Gareth W. Richings, Scott Habershon

**Affiliations:** Department of Chemistry, University of Warwick, Coventry CV4 7AL, UK

**Keywords:** quantum dynamics, MCTDH, diffusion maps

## Abstract

Grid-based schemes for simulating quantum dynamics, such as the multi-configuration time-dependent Hartree (MCTDH) method, provide highly accurate predictions of the coupled nuclear and electronic dynamics in molecular systems. Such approaches provide a multi-dimensional, time-dependent view of the system wavefunction represented on a coordinate grid; in the case of non-adiabatic simulations, additional information about the state populations adds a further layer of complexity. As such, wavepacket motion on potential energy surfaces which couple many nuclear and electronic degrees-of-freedom can be extremely challenging to analyse in order to extract physical insight beyond the usual expectation-value picture. Here, we show that non-linear dimensionality reduction (NLDR) methods, notably diffusion maps, can be adapted to extract information from grid-based wavefunction dynamics simulations, providing insight into key nuclear motions which explain the observed dynamics. This approach is demonstrated for 2-D and 9-D models of proton transfer in salicylaldimine, as well as 8-D and full 12-D simulations of *cis*-*trans* isomerization in ethene; these simulations demonstrate how NLDR can provide alternative views of wavefunction dynamics, and also highlight future developments.

## 1. Introduction

Accurately simulating wavefunction evolution describing the coupled nuclear/electronic dynamics of molecules following photo-excitation remains a frontier challenge in computational chemistry; refs. [1,2,3,4,5,6,7,8,9] the computational expense associated with accurately modelling excited state electronic structure, as well as that associated with representing and propagating time-dependent wavefunctions, means that there remain opportunities for algorithm development. Grid-based schemes in particular, where a time-dependent wavefunction is generally represented as a series of complex values on a coordinate-grid, broadly represent the most accurate strategy for modelling quantum molecular dynamics, refs. [1,10,11,12] but pay the price of large computational expense for increasingly large system sizes. Nevertheless, the extraordinary physical and chemical insight which can be gained from these highly-accurate dynamics simulations, including photospectra, refs. [13,14,15,16] relaxation rates, refs. [14,17] chemical reaction rates, refs. [18,19] reaction mechanisms and more, ref. [20] means that they remain a highly active area of research.

The predominant grid-based time-dependent wavefunction propagation scheme for many-dimensional systems is the multi-configuration time-dependent Hartree (MCTDH [2,6,21,22]) strategy and its multi-layer extensions [23,24,25,26,27,28,29]. Here, a time evolving wavefunction is represented as a sum of Hartree products of single particle functions (SPFs) which depend on a small number (typically one or two) of nuclear degrees-of-freedom. The SPFs are themselves represented on an underlying discrete variable representation (DVR [1,30,31,32,33]); in other words, the SPFs, and hence the full time evolving wavefunction, are ultimately represented on a grid of points distributed in coordinate space. Most commonly, the coordinates describing the system are taken to be vibrational normal mode coordinates derived from evaluation of the Hessian matrix at a minimum on the potential energy surface (PES), although alternative internal coordinates have also been explored in quantum dynamics simulations [6]. Equations governing the time evolution of the SPFs and the related expansion coefficients in the wavefunction approximation are obtained by direct application of the time-dependent variational principle, refs. [34,35] yielding a coupled set of equations-of-motion which can be used to propagate the multi-dimensional wavefunction. As well as modelling wavefunction evolution on a single Born-Oppenheimer electronic state, this scheme can be readily expanded to account for non-adiabatic simulations on multiple electronic states (using either the single-set or multi-set formulation [6]), enabling direct quantum dynamics simulations of molecular systems following photoexcitation.

The power of MCTDH lies in the use of a time-dependent set of SPFs to represent the terms in the overall sum of Hartree products. This time dependence, as well as the use of the time-dependent variational principle, ensures that the SPFs are optimally adapted to model the wavefunction dynamics, enabling smaller overall basis-set sizes when compared to quantum dynamics methods using fixed basis-sets. However, it is worth noting two important drawbacks of MCTDH. First is the typical requirement that the underlying potential energy surfaces (PESs), on which the time-dependent wavefunction evolves, be represented in a sum-of-products form; Refs. [2,6,36] as shown below, the MCTDH equations-of-motion demand that integrals over SPFs and the PES are performed in full dimensionality, and the sum-of-products PES form ensures that these integrals can be performed efficiently to enable propagation over significant time periods. Second, MCTDH operates in the so-called diabatic representation, requiring that the PESs describing multiple electronic states be diabatized before MCTDH propagation; while several schemes have been demonstrated to achieve this diabatization, these are ultimately approximations which should be carefully benchmarked [10,37,38,39,40,41,42,43,44,45,46].

In our own recent work, refs. [10,11,12,41,46,47,48,49] we have sought to address some of the challenges of MCTDH (and other grid-based schemes). In particular, we have shown how one can develop “on-the-fly” grid-based wavefunction propagation schemes in which an underlying PES is automatically and iteratively generated in tandem with wavefunction evolution. Here, kernel ridge regression (KRR), refs. [50,51] in combination with efficient sampling schemes, has been shown to be capable of automatically generating PESs in the required sum-of-products form for MCTDH propagation, enabling so-called “direct dynamics” simulations without the requirement of pre-fitting a PES. Subsequent work has demonstrated how efficient many-body tensor decomposition schemes, as well as improved additive kernel functions, can dramatically increase the computational efficiency of these direct-dynamics strategies. Finally, we have also proposed novel diabatization strategies, particularly focussed on commonly-used complete active-space (CAS) electronic structure methods, which enable automated generation of diabatic states for MCTDH propagation.

However, while we have demonstrated that these direct MCTDH simulations can be used to model (adiabatic and non-adiabatic) quantum dynamics in molecular systems containing tens of nuclear degrees-of-freedom, we have similarly found that the challenge of interpreting the outputs of such simulations increases with dimensionality. Most commonly, simple expectation values (e.g., position) for different nuclear degrees-of-freedom are used to interpret molecular motion, as are flux-operator expectation values across different reactive dividing surfaces; in non-adiabatic simulations, the diabatic state populations provide a similarly visual representation of the underlying dynamics, allowing extraction of approximate state lifetimes, for example. However, these strategies represent a first-order view of the complex coupled nuclear/electronic dynamics, whereas the full time-dependent wavefunction available in methods such as MCTDH provides a broader wealth of information about interactions between, and the importance of, different degrees-of-freedom at different stages of the emergent dynamics. As such, it is clear that alternative methods to analyse and extract important features of wavefunction evolution are an essential, yet often overlooked, part of general wavefunction propagation schemes.

In this article, following previous work on analysis of trajectory-based quantum simulations, ref. [52] we investigate how a non-linear dimensionality reduction (NLDR [53,54,55,56,57]) method, specifically diffusion maps (DMs [53,56]), can be adapted to provide insight into the important nuclear degrees-of-freedom during grid-based wavefunction propagation. NLDR methods such as DMs play an important role in analysing the outputs of simulations of complex systems, often represented by large numbers of coupled degrees-of-freedom; given a sequence of representative “snapshots” of a system’s state (for example, a sequence of molecular structures, in the present context), NLDR schemes can be used to identify a low-dimensional space (typically one or two coordinates) which capture the “essential” features associated with the input configurations. As an example, in simulations of complex protein folding processes or other biophysical organization phenomena, refs. [56,58] NLDR methods enable identification of important reaction-coordinates which might not be immediately obvious from simple time-series of individual atomic or molecular coordinates; furthermore, the reduction from the (typically) enormous number of atomic coordinates used in biophysical simulations to just a few key coordinates provides a further advantage to analysis. Here, building on these previous demonstrations of the utility of NLDR in other settings, we aim to investigate the use of NLDR in grid-based wavefunction propagation schemes; in particular, our goal in this article is to assess the utility of DMs in extracting important molecular motions from time-dependent wavefunctions as a route to ultimately understanding photo-induced chemical reaction mechanisms, and we demonstrate this approach in a series of grid-based simulations of multi-dimensional molecular systems.

The remainder of this article is organized as follows. First, we recap the important aspects of our direct grid-based strategy for wavefunction propagation, highlighting the use of KRR, tensor decomposition of the PES, and a novel diabatization scheme to enable efficient multi-dimensional/multi-state molecular simulations. Second, we outline how DMs can be adapted and applied to grid-based wavefunction simulation strategies. Third, we demonstrate our overall simulation scheme, and the possibilities offered by DMs, in large-scale simulations of salicylaldimine and ethene; notably, we perform the first direct, full-dimensional (12-D, three electronic states) MCTDH simulations of ethene, and use the DM NLDR strategy to compare our results to previous trajectory-based simulations of the same system. Finally, we conclude by highlighting the scope for further improvements in merging wavefunction propagation with NLDR schemes for analysis.

## 2. Materials and Methods

We begin by briefly outlining the direct grid-based schemes for wavefunction propagation which emerge by combining wavefunction evolution with automatic PES generation using KRR. We then describe how diabatic states are obtained in our simulations, before finally describing how analysis using DMs can be adapted to the case of grid-based dynamics.

### 2.1. Grid-Based Dynamics Methods: The Standard Method

Before describing the diffusion map method, we first present brief summaries of the grid-based dynamics methods to which this new implementation is applicable: the standard and multi-configuration time-dependent Hartree methods (SM and MCTDH respectively), both of which have been described in detail previously. Ref. [6] In this section we outline the SM, with the MCTDH being described in the next.

For a molecular system with *f* nuclear degrees-of-freedom (DOFs), the nuclear wavefunction is represented by a linear combination of products of time-independent basis functions, each of which is weighted by a complex, time-dependent coefficient, $C_{j_{1},\cdots ,j_{f}}$. The basis functions used in this method are in the discrete variable representation (DVR), ref. [6] which is a highly localised, orthonormal transformation of standard functions (sine, harmonic oscillator eigenfunctions etc.,) the products of which form a grid in configuration space. On an electronic state, *s*, the evolving wavepacket has the form

(1) $$\begin{array}{cc} \Psi^{\left(s\right)}\left(q_{1},\cdots ,q_{f},t\right)\; & =\sum_{j_{1}=1}^{N_{1}}\cdots \sum_{j_{f}=1}^{N_{f}}C_{j_{1},\cdots ,j_{f}}^{\left(s\right)}\left(t\right)\prod_{\kappa =1}^{f}\chi_{j_{\kappa }}^{\left(\kappa \right)}\left(q_{\kappa }\right)\\ \; & =\sum_{J}C_{J}^{\left(s\right)}\left(t\right)X_{J}\left(q\right), \end{array}$$

where we introduce a compound index, *J* = $j_{1},\cdots ,j_{f}$, to simplify notation. For a system of $N_{s}$ orthonormal electronic states, the total wavefunction is

(2) $$|\Psi \rangle =\sum_{s=1}^{N_{s}}|\Psi^{\left(s\right)}\rangle |s\rangle .$$

Assuming a total Hamiltonian (describing $N_{s}$ coupled electronic states) of the following general form:

(3) $$\hat{H}=\sum_{su}^{N_{s}}|s\rangle {\hat{H}}^{\left(su\right)}\langle u|,$$

we can then derive a set coupled equations-of-motion (EOMs), using the Dirac-Frenkel variational principle, refs. [59,60] for the expansion coefficients:

(4) $$i\hbar {\dot{C}}_{J}^{\left(s\right)}=\sum_{u=1}^{N_{s}}\sum_{L}\langle X_{J}|{\hat{H}}^{\left(su\right)}|X_{L}\rangle C_{L}^{\left(u\right)}.$$

Numerical integration of these EOMs, given some well-chosen initial wavefunction, gives a solution to the coupled nuclear/electronic TDSE. With an appropriate choice of DVR functions, and given a sufficiently large number of DVR functions, the time evolution of the wavepacket can be made numerically exact for the given initial conditions and Hamiltonian.

### 2.2. Grid-Based Dynamics Methods: MCTDH

Because of the exponential scaling of the computational effort of the SM, we are limited in practice to studying systems of about five DOFs or fewer. To overcome this constraint, the MCTDH method was developed. The key advance in MCTDH is the use of a sum-of-products of time-dependent basis functions (i.e., SPFs) rather than a time-independent basis, with each product term also having its own time-dependent coefficient. The MCTDH wavefunction *ansatz* is thus:

(5) $$\begin{array}{cc} \Psi^{\left(s\right)} & \left(Q_{1},\cdots ,Q_{f},t\right)\\ \; & =\sum_{j_{1}}^{n_{1}}\cdots \sum_{j_{m}}^{n_{m}}A_{j_{1},\cdots ,j_{m}}^{\left(s\right)}\left(t\right)\prod_{\kappa =1}^{m}\varphi_{j_{\kappa }}^{\left(s,\kappa \right)}\left(Q_{\kappa },t\right)\\ \; & =\sum_{J}A_{J}^{\left(s\right)}\left(t\right)\Phi_{J}^{\left(s\right)}\left(Q,t\right), \end{array}$$

with $A_{j_{1},\cdots ,j_{m}}^{\left(s\right)}\left(t\right)$ being the coefficients, and $\varphi_{j_{\kappa }}^{\left(s,\kappa \right)}\left(Q_{\kappa },t\right)$ the $j_{\kappa }$-th SPF on the sth electronic state which is a function of a mode which is a subset of the DOFs in question $Q_{\kappa }=\left(q_{\kappa_{1}},\cdots ,q_{\kappa_{p}}\right)$ (a single SPF describes one or a few DOFs [2,6,21]). For compactness, we again use the compound index $J=j_{1},\cdots ,j_{m}$.

As with the SM, EOMs are derived from the MCTDH *ansatz* using the Dirac-Frenkel variational principle, giving EOMs for both the coefficients and the SPFs, respectively, as follows:

(6) $$i\hbar {\dot{A}}_{J}^{\left(s\right)}=\sum_{u}^{N_{s}}\sum_{L}\langle \Phi_{J}^{\left(s\right)}|{\hat{H}}^{\left(su\right)}|\Phi_{L}^{\left(u\right)}\rangle A_{L}^{\left(u\right)},$$

and

(7) $$i\hbar {\dot{\varphi }}^{\left(s,\kappa \right)}=\left(1-{\hat{P}}^{\left(s,\kappa \right)}\right){\left(\rho^{\left(s,\kappa \right)}\right)}^{-1}\sum_{u}^{N_{s}}{\langle {\hat{H}}^{\left(su\right)}\rangle }^{\left(\kappa \right)}\varphi^{\left(u,\kappa \right)}.$$

Here, we define the following: $1_{n_{\kappa }}$ is the unit matrix; ${\hat{P}}^{\left(s,\kappa \right)}$ is a projector onto the SPF space along mode κ; ${\hat{h}}^{\left(\kappa ,s\right)}$ are the one-dimensional (1D) operators; and ${\left(\rho^{\left(s,\kappa \right)}\right)}^{-1}$ is the density matrix inverse associated with κ. We can construct a Hartree product of SPFs in all modes excluding the κth to give a function, $\Phi_{J^{\kappa }}^{\left(s\right)}$, from which we can build a set of single-hole functions, $\Psi_{l}^{\left(s,\kappa \right)}=\sum_{J^{\kappa }}A_{J_{l}^{\kappa }}^{\left(s\right)}\Phi_{J^{\kappa }}^{\left(s\right)}$, which are then used to construct a mean-field matrix with elements ${\langle {\hat{H}}^{\left(su\right)}\rangle }_{jl}^{\left(\kappa \right)}$=$\langle \Psi_{j}^{\left(s,\kappa \right)}|{\hat{H}}^{\left(su\right)}|\Psi_{l}^{\left(u,\kappa \right)}\rangle$.

To integrate the SPF EOMs (Equation (7)), the SPFs are expanded as sums-of-products of DVR basis functions along each of the $\kappa_{p}$ DOFs described by $Q_{\kappa }$ i.e.,

(8) $$\varphi_{j_{\kappa }}^{\left(s,\kappa \right)}\left(Q_{\kappa },t\right)=\sum_{i_{\kappa }}^{N_{\kappa }}c_{i_{\kappa }}^{\left(s,\kappa ,j_{\kappa }\right)}\left(t\right)X_{i_{\kappa }}^{\left(\kappa \right)}\left(Q_{\kappa }\right),$$

where $X_{i_{\kappa }}^{\left(\kappa \right)}\left(Q_{\kappa }\right)$ is the $i_{\kappa }$-th basis function for mode $Q_{\kappa }$; a direct product of the DVR bases along those DOFs.

Even though the computational effort of MCTDH ultimately scales exponentially in the number of DOFs, the use of time-dependent SPFs allows the dimensionality of the basis to be kept optimally small, reducing the base of the scaling exponential. As a result a few tens of DOFs can be studied with MCTDH [6].

### 2.3. Procrustes Diabatization

In this article, we will consider systems evolving on multiple electronic states. As described below, our grid-based direct-dynamics strategy constructs PES functions “on-the-fly”, concurrent with the propagation of the nuclear wavepacket, using the output from electronic structure codes (in our case Molpro [61]). The energies and states produced by such codes are in the so-called adiabatic representation, where the electronic states are ordered by increasing energy at a given nuclear configuration. However, at points where two states, *i* and *j*, are degenerate i.e., the respective energies, $V_{ii}^{A}$ and $V_{jj}^{A}$, are equal, the non-adiabatic coupling terms (NACTs) between them,

(9) $$F_{ij}=\frac{\langle \psi_{i}|\nabla {\hat{H}}_{el}|\psi_{j}\rangle }{V_{jj}^{A}-V_{ii}^{A}},$$

diverge and the gradients of the states with respect to the nuclear geometry are discontinuous. It is difficult to fit such surfaces (and also difficult to deal with discontinuous coupling terms) using a small number of smooth functions, so we transform the states to the diabatic representation (strictly, the quasi-diabatic representation as we do not have an infinite set of states, but we will use the shorter terminology for compactness) by finding a geometry-dependent, unitary transformation of the states which minimises the NACTs and smooths the PESs. The result is set of crossing surfaces interacting via finite, potential-like diabatic coupling terms, which can be efficiently fit using smooth functions.

There are many ways to diabatise electronic states, but the method we use here, enabling diabatisation on the fly, is Procrustes diabatisation. We have described this method in detail previously, refs. [12,46] but the general idea is to take the diabatic states, $\left\{|\Psi_{i}^{D}\left(q\right)\rangle \right\}$ at a geometry, represented by the coordinates q, near to the one of interest, q+Δq, where we have the adiabatic states $\left\{|\Psi_{i}^{A}\left(q+\Delta q\right)\rangle \right\}$, and overlap them to give a matrix $S_{ij}=\langle \Psi_{i}^{D}\left(q\right)|\Psi_{j}^{A}\left(q+\Delta q\right)\rangle$. The orthogonal Procrustes method is then used to find the orthogonal matrix, A(q+Δq), which transforms S to be a close as possible to the unit matrix. In this way the resulting diabatic states at geometries throughout configuration space maintain their ’character’. The resulting matrix, A, is used to transform the diagonal, adiabatic energy matrix to the diabatic representation:

(10) $$V^{D}\left(q\right)=A^{T}\left(q\right)V^{A}\left(q\right)A\left(q\right).$$

There is a constant global phase in the transformation matrix, which means that we can set $\{|\Psi_{i}^{D}\left(q_{0}\right)\rangle \}=\{|\Psi_{i}^{A}\left(q_{0}\right)\rangle \}$ at the reference geometry, $q_{0}$, (chosen to be the location of the centre of the initial wavepacket here), and hence $A(q_{0})=I$. By moving outwards from $q_{0}$ we can diabatise states at geometries of increasing distance from the reference point, hence we end up with diabatic energies and couplings everywhere.

### 2.4. Machine-Learning PESs for Grid-Based Wavefunction Propagation

In grid-based wavefunction propagation schemes, such as MCTDH, a typical requirement is that the diabatic energies and coupling are fit to a set of appropriate functions, giving a PES on which the nuclear wavepacket propagates. This requirement of pre-fitting diabatic states and couplings is a well-known hurdle to achieving “on the fly”, or direct-dynamics, simulations. In our recent work, described in detail previously, refs. [10,11,41,47,49] we have developed a grid-based direct-dynamics scheme which uses kernel ridge regression (KRR) as the underlying regression tool; we give a brief summary of it here.

Given a set of *M* geometries represented by coordinates, $\left\{q^{l}\right\}$, where we know the diabatic energies and couplings (we will say more about coordinates, and about how this set is created, later), we define a kernel function along each DOF λ as:

(11) $$k\left(q_{\lambda },q_{\lambda }^{l}\right)=e^{-\alpha_{\lambda }{\left(q_{\lambda }-q_{\lambda }^{l}\right)}^{2}}.$$

This function is centred at $q_{\lambda }^{l}$, and its width is controlled by the real parameter $\alpha_{\lambda }$.

For low-dimensional problems (f≤3) we form a full product kernel from the 1D kernel in Equation (11),

(12) $$k^{\mathrm{Full}}\left(q,q^{l}\right)=\prod_{\lambda =1}^{f}k\left(q_{\lambda },q_{\lambda }^{l}\right),$$

and for larger systems with f>4, an additive kernel which couples up to two DOFs, is used,

(13) $$k^{\mathrm{Add}}\left(q,q^{l}\right)=\sum_{\lambda =1}^{f}k\left(q_{\lambda },q_{\lambda }^{l}\right)+\sum_{\lambda <\mu }^{f}k\left(q_{\lambda },q_{\lambda }^{l}\right)k\left(q_{\mu },q_{\mu }^{l}\right).$$

We have discussed the reasons for using the different kernels previously [49].

The subsequent calculations and reasoning hold for the kernels in Equations (12) and (13) so we use $k(q,q_{i})$ to represent both for clarity. For electronic states *s* and *u*, the diabatic energies (s=u) and coupling (s≠u) are represented as

(14) $$V_{\left(su\right)}^{\mathrm{KRR}}\left(q\right)\approx \sum_{l=1}^{M}w_{l}^{\left(su\right)}k\left(q,q_{l}\right),$$

where the lth kernel has a weight $w_{l}^{\left(su\right)}$, determined by solving (using Cholesky decomposition), the matrix equation

(15) $${\mathrm{Kw}}^{su}=b^{su}.$$

Here, $w^{su}$ is the vector containing the weights, and the elements of b are

(16) $$b_{i}^{\left(su\right)}=V^{\left(su\right)}\left(q_{i}\right).$$

The matrix K is the covariance matrix with elements [50]

(17) $$K_{mn}=k\left(q^{m},q^{n}\right)+\gamma^{2}\delta_{mn},$$

with $\gamma^{2}$ (set to $10^{-8}$ in our case) used to regularize the equations.

To determine which points to sample and include in the set of energies/couplings, used to generate the PESs in Equation (14), we periodically sample a given number of coordinates around the wavepacket centre (within three wavepacket widths along each DOF) on each state at the current propagation time using Sobol sequences [41,62,63,64]. In deciding whether to add the sampled points q to the current set of energies/couplings, the variance function, ref. [50]

(18) $$\sigma^{2}\left(q\right)=k\left(q,q\right)+\gamma^{2}-k^{T}K^{-1}k$$

is calculated, where $k_{i}=k\left(q,q_{i}\right)$. This function represents an estimate of the error in the current PES at the selected point; if the calculated variance is greater than a pre-defined input parameter, the selected point is added to the list of reference points for Equation (14), and the weights are updated accordingly. If the calculated variance is less than the threshold value, the current fit of the PESs at q is deemed sufficiently accurate and the point is not considered further, without having to calculate the energies and couplings at this configuration. In this way the PES is automatically sampled in regions where the wavepacket moves, building up a database of energies and couplings as the propagation progresses, hence improving the fit of the PESs as time moves on.

Although KRR can generate accurate PES representations for quantum dynamics, the number of terms in the PES expansion means it can be inefficient to evaluate the MCTDH EOMs (Equations (6) and (7)); as such, a further approximation is required. In passing, we note that no such problem exists for the SM, so the raw KRR PESs are used in this case. To improve efficiency of MCTDH calculations, a secondary fitting procedure decomposes the KRR PESs into terms of one and two dimensions [49]. Briefly, to obtain the 1D terms, the KRR PESs are simply evaluated at the locations of the DVR gridpoints along each DOF (all other coordinates being set to 0), the value of the relevant PES at the origin being subtracted from all but one of the DOFs to avoid double counting. The result is a set of *f* vectors, $\left\{V^{\lambda }\left(q_{\lambda }^{l}\right)\right\}$, for each diabatic state and interstate couplings.

To construct the 2D terms, which couple wavepacket motion between each pair of DOFs, we consider each pair in turn and form a grid from the DVRs along each (all other coordinates set to 0); at each point, we calculate the $N_{\lambda }\times N_{\mu }$ residue matrix (where $N_{\lambda }$ and $N_{\mu }$ are the numbers of DVR points along each DOF, λ and μ):

(19) $$V^{\lambda \mu }\left(q_{\lambda }^{l},q_{\mu }^{m}\right)=V^{\mathrm{KRR}}\left(q_{\lambda }^{l},q_{\mu }^{m}\right)-V^{\lambda }\left(q_{\lambda }^{l}\right)-V^{\mu }\left(q_{\mu }^{m}\right)-V^{\mathrm{KRR}}\left(0\right).$$

A singular value decomposition (SVD) is then used to reduce this matrix as

(20) $$V^{\lambda \mu }\left(q_{\lambda }^{l},q_{\mu }^{m}\right)=\sum_{j=1}^{\min(N_{\lambda },N_{\mu })}\sigma_{j}\;u_{lj}\;w_{mj}$$

and thus to a sum of outer-products of vectors along each of the two DOFs

(21) $$V^{\lambda \mu }=\sum_{j=1}^{\min(N_{\lambda },N_{\mu })}V_{\lambda (j)}^{\lambda \mu }⊗V_{\mu (j)}^{\lambda \mu }$$

where $V_{\lambda (j)}^{\lambda \mu }\left(q_{\lambda }^{l}\right)=\sqrt{\sigma_{j}}u_{lj}$ and $V_{\mu (j)}^{\lambda \mu }\left(q_{\mu }^{m}\right)=\sqrt{\sigma_{j}}w_{mj}$. This PES decomposition is in the sum-of-products form, as required for MCTDH, with many fewer terms than the original KRR fit, hence increasing computational efficiency.

We do not necessarily include all terms from the SVD as some may be insignificant, adding little to the accuracy of the fit whilst increasing computational effort, so by defining

(22) $$\left||R|\right|=\sum_{j=n+1}^{\min(N_{\lambda },N_{\mu })}\sigma_{j}^{2}.$$

with the singular values in decreasing order, we only include *n* terms such that ||R|| is below a pre-defined accuracy parameter (here taken to be $10^{-3}$).

### 2.5. Diffusion Maps for Grid-Based Dynamics

With an outline of our KRR-based direct-dynamics scheme using grid-based propagation, we now turn to the new aspect studied in this article, namely the integration of NLDR, specifically using DMs, to analyse grid-based wavefunction dynamics.

To analyse grid-based dynamics calculations using DMs, we have implemented a new analysis program, diffmap, within the Quantics package [65] which can post-process the results of both SM and MCTDH propagations, irrespective of whether the PESs have been pre-fitted or constructed on-the-fly. The first requirement, of course, is the wavefunction resulting from such a calculation which may involve propagation on one or more electronic states, and which is stored as a set of wavefunctions at *T* pre-determined time-intervals i.e., $\left\{\Psi \left(t_{n}\right),n=1,\ldots ,T\right\}$.

When analysing the wavepacket with diffmap there are several parameters which must be chosen before running the program (although defaults are provided); these will be described as they arise in the following, but the first is the state of interest (in other words, although the wavepacket may evolve on multiple coupled states, our DM analysis is performed on a *per* state basis). We must also choose the parameters to control wavepacket sampling, specifically which of the time-ordered set of individual wavefunctions are sampled. Currently, diffmap provides options to: (i) skip some of the initial timesteps, and (ii) sample from every nth timestep. Because we may expect the wavefunction to change relatively little between consecutive timesteps, sampling every timestep adds little insight, but increases computational effort. Finally, diffmap also requires the user to specify how many geometries are sampled at each timestep, $N_{\mathrm{samp}}$. Typically, we are only interested in sampling regions of high wavepacket density to see the general motion of the wavepacket, so we need to know the density at the selected geometries so as to determine whether to keep or discard sample points in DM analysis. Furthermore, because we are performing dynamics on a DVR grid, it is only possible to calculate the density at these gridpoints, hence we only sample geometries corresponding to DVR gridpoints when performing DM analysis.

The first step in the DM analysis performed by diffmap is the sampling of the gridpoints populated by the wavepacket at the current chosen timestep; the same procedure is carried out for each of the selected timesteps. For the wavefunction at time, $t_{n}$, which is a function of *F* active DOFs, $q=(q_{1},\ldots ,q_{F})$, i.e., $\Psi (q,t_{n})$, we first locate the centre and width of the wavepacket along each DOF, $q_{f}$, calculating $\langle q_{f}\rangle =\langle \Psi |{\hat{q}}_{f}|\Psi \rangle$ and $\langle \delta q_{f}\rangle =\sqrt{\langle q_{f}^{2}\rangle -{\langle q_{f}\rangle }^{2}}$, respectively. The DVR gridpoint nearest to the centre is noted as are the first gridpoints beyond $\langle q_{f}\rangle \pm 3\langle \delta q_{f}\rangle$ (or the terminal gridpoint if three widths of the wavefunction from the centre extends beyond the limit of the grid). These two outer gridpoints then define the range in which sampling takes place; by repeating for all *F* DOFs, we describe a hypercuboid encapsulating the wavefunction, in which the sampling occurs.

The first sampled gridpoint is that closest to the wavepacket centre, $q_{0}$, where we evaluate the density, $\rho_{0}=\sqrt{\langle \Psi \left(q_{0},t_{n}\right)|\Psi \left(q_{0},t_{n}\right)\rangle }$. This value is used to determine which of the subsequently sampled gridpoints are retained and which are discarded. A user-provided parameter, $\beta_{\min}$, determines that gridpoints, q, where $\rho =\sqrt{\langle \Psi \left(q,t_{n}\right)|\Psi \left(q,t_{n}\right)\rangle }\geq \beta_{\min}\rho_{0}$ are retained when sampled, while those that do not fulfil this condition are discarded. In this way we sample regions of relatively high wavefunction density only. The gridpoints sampled are chosen by using Sobol sequences along each DOF to find each $q_{f}$ (and hence q). The Sobol sequence gives a quasi-random number between 0 and 1, whilst the domain between 0 and 1 is partitioned equally into a number of segments equal to the number of gridpoints in the sampling region along the current DOF; the number given by the Sobol procedure is in one of these segments, hence giving the gridpoint selected. We use this sampling scheme, rather than checking all gridpoints q, as it is quite possible for the number of combinations of q to grow very large, potentially exceeding the integer overflow range. The process of selecting gridpoints using a Sobol sequence, then checking the wavepacket density to determine whether to save or discard the point, continues until we have saved the pre-defined number of points. From a practical viewpoint, we have found that it may be necessary to reduce either $N_{\mathrm{samp}}$ or $\beta_{\min}$ to speed up the sampling, or if there are insufficient points with large wavefunction density within the sampling region.

Once the sampling of gridpoints has been completed for all chosen timesteps, DM analysis proceeds by examining the eigenvectors of a diffusion operator defined as follows. First, for all sampled gridpoints (noting that all points sampled at all timesteps are compared in a single set), we form the kernel array

(23) $$k_{ij}=\exp{\left(-\frac{|q_{i}-q_{j}|}{D}\right)}^{2},$$

where *D* is a user-defined parameter which moderates the correlation between distant points. We note that Euclidean distances between the gridpoints are used; in this work we use mass-frequency scaled normal modes as coordinates. Within the direct-dynamics method using this coordinate system, where electronic structure calculations are performed on-the-fly, all molecular geometries are in the same Eckart frame defined by the normal modes at the reference geometry, hence bringing the geometries into maximum coincidence.

Next, we calculate a normalisation vector

(24) $$v_{i}=\sum_{j=1}^{N_{\mathrm{samp}}}k_{ij},$$

and a re-normalisation vector

(25) $$Z_{i}=\sum_{j=1}^{N_{\mathrm{samp}}}\frac{k_{ij}}{{\left(v_{i}v_{j}\right)}^{\alpha }},$$

from which we construct the diffusion operator as

(26) $$a\left(q_{i},q_{j}\right)=\frac{1}{Z_{i}}\frac{k_{ij}}{{\left(v_{i}v_{j}\right)}^{\alpha }}.$$

In these latter two equations, the user-provided parameter, α, defines the diffusion model in use: α=0 gives Laplacian diffusion, α=1/2 is an approximation to the backward Fokker-Planck operator, and α=1 approximates Laplace-Beltrami heat diffusion. Following Virshup et al. [52] we use α=1 here, as this results in a diffusion map which is invariant to the density of the sampled points.

Finally, the eigenvectors of the matrix a (Equation (26)) with the six lowest-value eigenvalues are found using the implicitly-restarted Arnoldi method as implemented in the ARPACK package [66]. The eigenvectors are $N_{\mathrm{samp}}$-dimensional, each component representing the coordinate of the corresponding sampled gridpoint within the reduced dimensionality space given by the selected eigenvector. The eigenvector with the largest eigenvalue (of 1) with all components equal is ignored, as it simply shows that all points are part of a single cluster. By examining the eigenvalues of the second to sixth eigenvectors, we look for a large step in the eigenvalues; the eigenvectors before this jump are the dominant ones which determine the dimension of the reduced space. Using the sampled grid-points, the wavefunction dynamics within the reduced dimensional space can then be studied, enabling identification of important DOFs.

The above methodology, implemented in diffmap, enables DM analysis to be performed for grid-based wavefunction dynamics, implemented in Quantics using SM and MCTDH. In the following section, we illustrate the utility of this scheme by studying the adiabatic and non-adiabatic dynamics of many-dimensional molecular systems.

## 3. Results

To test our implementation of the DM method for grid-based nuclear dynamics we will focus on two representative examples: (i) the ground-state proton transfer dynamics in salicylaldimine (using 2D and 9D models), and (ii) the excited state *cis*-*trans* isomerisation of ethene (using 8D and 12D models).

### 3.1. Dynamics of Salicylaldimine

First, we analyse dynamics calculations on the ground-state proton transfer in salicylaldimine. We and others [67] have studied this reaction previously, and so have a good idea of the important motions involved in the dynamics; as a result, this is an ideal system on which to test the DM method.

We have carried out a series of dynamics calculations with an increasing number of DOFs included in order to evaluate whether the DM analysis can sufficiently reveal the key underlying molecular motions associated with the emergent dynamics. In all calculations we used the VCHAM-fitted potential of Polyak et al. [67] either directly in standard grid-based calculations or to evaluate individual energy values when performing direct-dynamics/grid-based (DD-GB) calculations using KRR. These dynamics calculations all used mass-frequency scaled normal modes as the coordinate system, the normal modes having been calculated at the location of the proton transfer transition state. In Table 1 we present the modes, along with details of the DVR bases and initial wavepacket conditions along each mode used in all of the subsequent calculations.

#### 3.1.1. 2D Salicylaldimine

As a first test of the DM method we performed 2D calculations using modes 1A’ and 36A’; these modes are the imaginary frequency mode representing the motion of the proton between the N and O atoms, and the in-plane bending mode of the same proton respectively. This calculation was a ‘direct-dynamics’ SM propagation (DD-SM) in which KRR is used to generate the PESs but the SM is used for wavefunction propagation. The calculations used full kernel KRR (Equation (12)) with α=0.02 along both modes; 300 gridpoints were sampled every 1 fs (within three widths of the current wavepacket centre). Points were added to the database of energies if the calculated variance (Equation (18)) was greater than $10^{-6}$. The wavepacket was propagated for a total of 100 fs using the default short-iterative Lanczos integrator in Quantics. The results were compared to standard grid-based dynamics on the full PES to ensure that the sampling and kernel width reproduced the full PES (and dynamics) accurately. In our KRR-based simulations, a total of 41 gridpoints were ultimately used to construct the KRR database.

Subsequently, we analysed the wavepacket using the DM method. To construct the DM, 10 gridpoints were sampled every 20 fs, with $\beta_{\min}=0.2$. In total, this gave a sample set of 60 gridpoints. Furthermore, we used D=2 in Equation (23) for the DM analysis.

**Figure 1:**
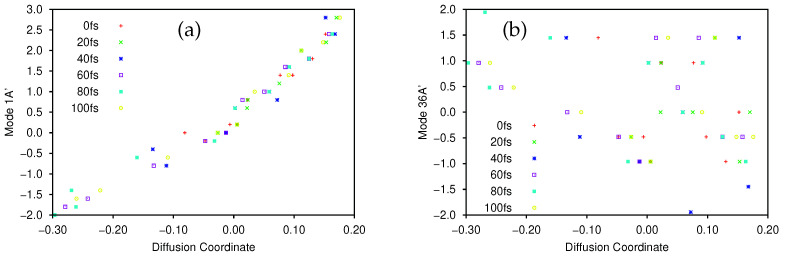
Sampled normal mode coordinates of salicylaldimine plotted as functions of the diffusion coordinate: (**a**) Mode 1A’; (**b**) Mode 36A’. In both cases data points are labelled according to the sample time; gridpoints were sampled every 20 fs during the dynamics.

The largest (non-trivial) eigenvalues of the diffusion operator were 0.630, 0.364 and 0.286. Following Virshup et al. [52] we note that there is a large drop between the first and second greatest eigenvalues, which indicates that the motion is well-described by the first non-trivial eigenvector. As such, in Figure 1 we present plots of the normal mode coordinates as functions of the associated diffusion coordinate (given by the elements of the eigenvector). Figure 1a gives the plot of the mode 1A’ coordinate against the diffusion coordinate, whilst Figure 1 shows the same plot using mode 36A’ coordinates. Note that different symbols are used for points sampled at different times.

The main point to note is the difference in the correlation between the normal mode and diffusion coordinates; there is strong correlation between the 1A’ normal mode and the diffusion coordinate, whilst there is little correlation between the 36A’ mode and the diffusion coordinate. This indicates that the dynamics is dominated by motion along the 1A’ mode while movement along the bending mode contributes much less to the overall motion; this is exactly what we would expect because the normal modes were calculated at the peak of the transition barrier which separates the PES minima at the keto and enol tautomer geometries. With the initial wavepacket starting on the enol side of the barrier (but not at the minimum), there is tunnelling motion to the keto side along the 1A’ mode, followed by motion back and forth, through and across the barrier. The bending mode acts more as a bath with relatively uncorrelated motion i.e., the wavepacket spreads along the mode during propagation rather than contributing to the overall motion.

The motion of the wavepacket is even clearer when considering Figures S1 and S2 in the Supplementary Materials, where the data in Figure 1a,b, respectively, are represented in a form where each sample time is allocated its own plot. In Figure S1 we see that, at 0 fs, the wavepacket is sampled at normal mode coordinates between −0.5 and 2.5, whilst after 20 fs the sampled points are located between −0.5 and 3.0. After this time the sampled points begin to be found with normal mode coordinates as low as −2.0 (diffusion coordinate slightly greater than −0.3) indicating motion across the potential barrier, along mode 1A’, as expected. Looking at Figure S2 we see that the sampled normal mode coordinates do not change greatly over time; the diffusion coordinate changes with time but each of those coordinates corresponds to a similar range of the normal mode coordinates, mainly between −1 and 3 (the only outliers being found at 40 fs where the range extends to a normal mode coordinate of −2), reflecting the lack of correlation between mode 36A’ and the diffusion coordinate.

**Figure 2:**
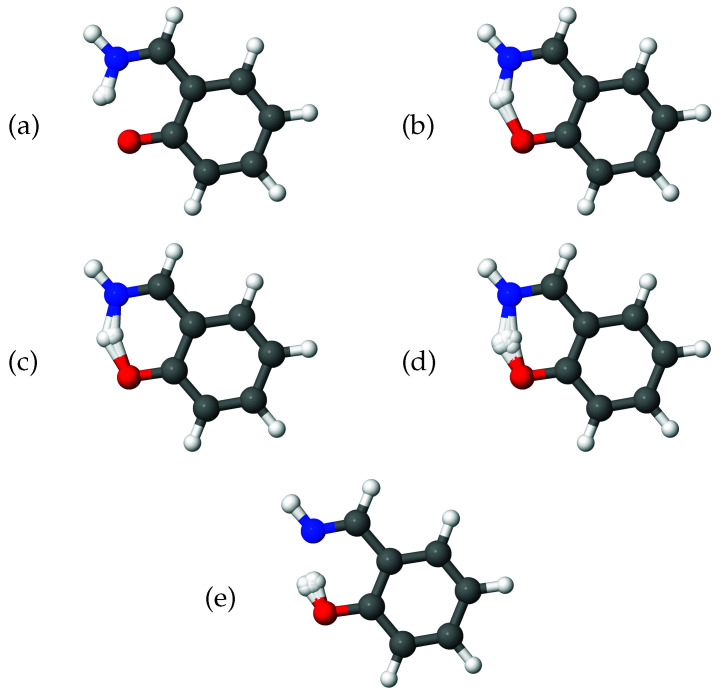
Sampled geometries of salicylaldimine grouped according to their associated diffusion coordinate, $\theta_{2}$: (**a**) $-0.3<\theta_{2}<-0.2$; (**b**) $-0.2\leq \theta_{2}<-0.1$; (**c**) $-0.1\leq \theta_{2}<0.0$; (**d**) $0.0\leq \theta_{2}<0.1$; (**e**) $0.1\leq \theta_{2}<0.2$.

As a further illustration of the nature of the diffusion coordinate, we present the plots in Figure 2, which show the overlaid geometries of salicylaldimine, sampled during the diffusion map analysis and grouped according to their associated diffusion coordinate, $\theta_{2}$ (ignoring the time-labelling of configurations). In Figure 2a, we show geometries with $-0.3<\theta_{2}<-0.2$ and it is clear to see that all of the geometries can be characterised as keto tautomers, with the tunnelling hydrogen bonded to the nitrogen atom (blue). Moving to increasing values of $\theta_{2}$ we see from Figure 2b,e, which represent geometries with diffusion coordinates of, respectively, $-0.2\geq \theta_{2}<-0.1$, $-0.1\geq \theta_{2}<0.0$, $0.0\geq \theta_{2}<0.1$ and $0.1\geq \theta_{2}<0.2$, that the hydrogen atom moves across to the oxygen atom (red) to give the imine tautomer. In all of the plots the geometries are quite tightly clustered together which indicates that the reaction path is fairly narrow i.e., that $\theta_{2}$ is the dominant variable within the dynamics. This width is associated with motion in the 36A’ bending mode, which, as we have seen in Figure 1b is poorly correlated with the overall reaction.

It may be worth saying something here about the choice of parameters in the analysis, particularly the density threshold coefficient, $\beta_{\min}$, (here set to 0.2) and the kernel scaling parameter, *D*. First, if the density threshold, $\beta_{\min}$ is too large, then the sampling of gridpoints inevitably slows down because more points have to be checked before the required number of points (those with sufficiently high density) are located. We have re-analysed the wavepacket propagation using various values of $\beta_{\min}$ (keeping all other parameters as used in the original analysis) and the results are presented in Table 2. In this case the calculation took less than 1 s in all cases so the slowdown was negligible, but can become significant for larger systems where calculation of the density at each gridpoint takes longer (in MCTDH calculations the density is evaluated by combining the potentially numerous SPF and wavefunction expansion coefficients).

Examining the results in Table 2 we see that the largest non-trivial eigenvalues increased when the density threshold $\beta_{\min}$ is decreased. We also see that, of the values tested, the largest proportional spectral gap was found when $\beta_{\min}=0.2$, meaning that the decomposition of the dynamics into a single mode is most obvious when $\beta_{\min}=0.2$ because the first non-trivial eigenvector most clearly dominates.

Next we consider the effect of changing the kernel distance parameter, *D*. In Table 3 we present results for the two largest, non-trivial eigenvalues using different values of *D*; in all cases, $\beta_{\min}=0.2$ and all other parameters are the same as for the original analysis above. From this Table we see that the eigenvalues increase as *D* decreases, but the proportional gap between them decreases (aside from small rises when *D* is very large). If D=0.5 or D=1 then there is not a significant spectral gap between the two non-trivial eigenvalues, indicating that the analysis has failed to find a single dominant mode to characterise the dynamics, in contrast to those analyses using larger values of *D*. In both cases D=0.5 and D=1, there are also no significant gaps between successive eigenvalues up to $\epsilon_{6}$ at least (only these six eigenvalues are reported), so the dimensionality reduction appears to have failed. Furthermore, for D=0.5 we note that the first eigenvalue, $\epsilon_{1}$, is no longer equal to one as it is in all other cases, indicating that the diffusion operator is no longer producing a trivial first eigenvector. Furthermore, in this case we also find that the plots of each normal mode coordinate against the diffusion coordinate no longer resembles those in Figure 1; the correlation between the diffusion coordinate and the 1A’ normal mode is lost. Virshup et al. [52] noted that *D* should be large enough that discretisation effects are smoothed out but small enough not to obscure large scale features. Our brief analysis of the *D* parameter here supports this; D≥2 seems to be reasonable in the 2D salicylaldimine case. We should note that the PES here is fairly simple, being an asymmetric double well in two dimensions, so using a large value of *D* causes no issues; the largest eigenvalues decrease but the proportional spectral gap stays large and the correlation between the diffusion coordinate and the 1A’ normal mode is still clear (as is the lack of correlation with mode 36A’), even when D=100.

#### 3.1.2. 9D Salicylaldimine

Moving on to a more complex system, we consider 9D MCTDH calculations for salicylaldimine. In this case we use the DVRs and initial wavefunction conditions as outlined in Table 1, but here we use the following combinations of normal modes: {1A’}; {32A’,36A’}; {5A’,7A’}; {9A’,10A’}; {11A’,13A”}. Here the notation {*mode a, mode b*} indicates that the enclosed normal modes are treated as a single combined mode i.e., they are represented by 2D SPFs in the MCTDH equations-of-motion; for all five combined modes 6 SPFs were used. The wavepacket propagation was run for 100 fs using the default Adams-Bashforth-Moulton (ABM) integrator in the Quantics package. To represent the PES we used KRR, sampling energies from the Polyak potential. Every 1 fs $10^{3}$ coordinates were sampled, with energies being added to the database if the KRR variance at the sampled point exceeded $10^{-3}$. The 2D additive kernel (Equation (13)) was used to fit the PES using α=0.02 along each DOF. A secondary SVD fitting was performed on the KRR PES, with sufficient 2D terms being retained to ensure the residue ||R|| was less than $10^{-3}$ (Equation (22)).

After wavepacket propagation, time-dependent wavefunctions were analysed by DM, using a kernel scaling parameter of D=3 and a sampling threshold with $\beta_{\min}=0.2$. 20 gridpoints were sampled every 10 fs. The first three non-trivial eigenvalues were 0.476372, 0.176858 and 0.161721, giving a spectral gap ratio of 0.371 between the first and second eigenvectors, suggesting that the 9D dynamics can be reduced to a single, dominant mode. The eigenvector with the largest eigenvalue defines the diffusion coordinate, $\theta_{2}$.

**Figure 3:**
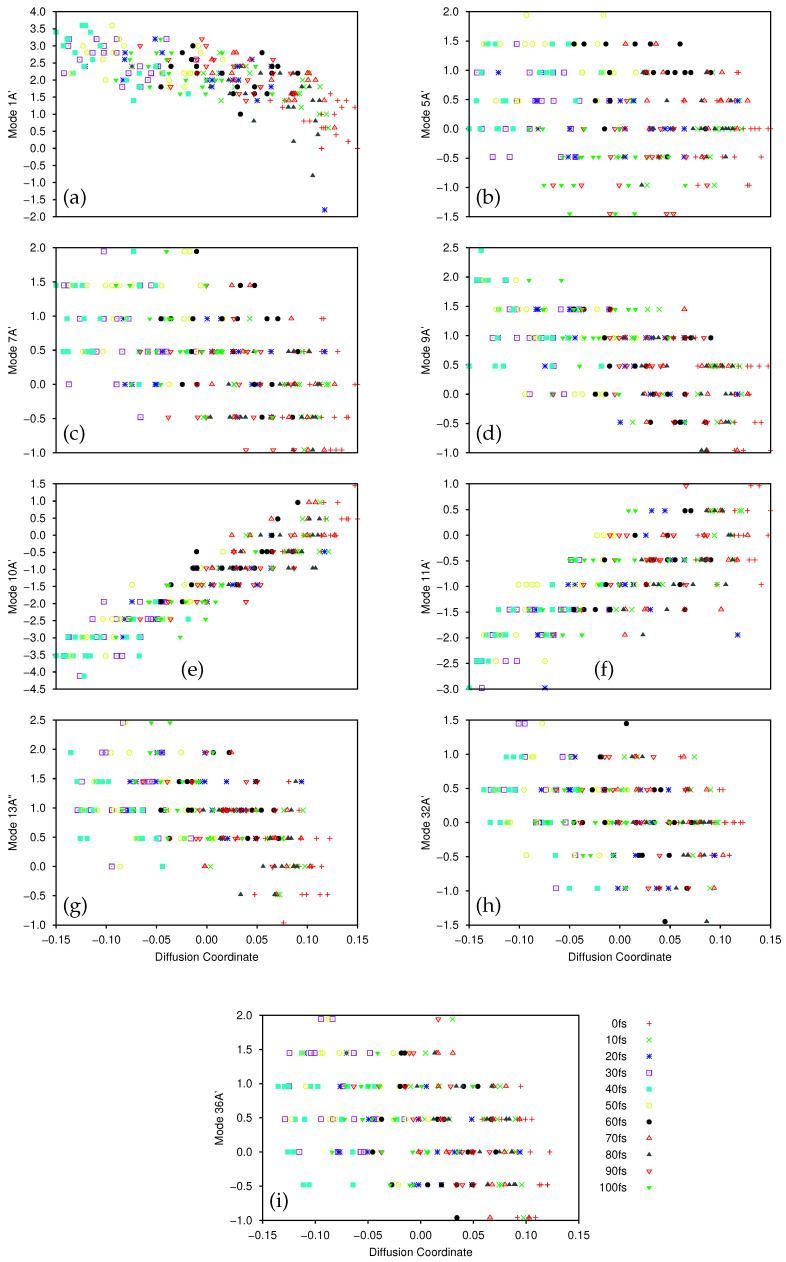
Sampled normal mode coordinates of salicylaldimine plotted as functions of the diffusion coordinate: (**a**) Mode 1A’; (**b**) Mode 5A’; (**c**) Mode 7A’; (**d**) Mode 9A’; (**e**) Mode 10A’; (**f**) Mode 11A’; (**g**) Mode 13A”; (**h**) Mode 32A’; (**i**) Mode 36A’. In all cases data points are coloured according to the sample time; 20 gridpoints were sampled every 10 fs during the dynamics. The key to the right of plot (**i**) applies to all plots.

In Figure 3 we plot the normal mode coordinates of the sampled points against the diffusion coordinate $\theta_{2}$, with points coloured according to their sample time. From these nine plots we see that the clear correlation of the 1A’ mode with the diffusion coordinate is weakened; without the sampled points with mode coordinates of less than −0.5 (bottom right of Figure 3a) this would be even less apparent. Of the other modes, the diffusion coordinate has the clearest dependence on 10A’ with weaker dependencies on 11A’ and, even less so, on 9A’ (see Figure 2 in Polyak et al. [67] for diagrams of the vibrational modes), but 10A’ can be characterised as the bending of the oxygen and the carbon bonded to the nitrogen towards one another, whilst 9A’ is a bending of the OH group towards the ring and 11A’ is motion of the oxygen and nitrogen towards one another. The time dependence of the diffusion map can be seen more clearly by considering Figures S3–S11 in the Supplementary Materials, which are versions of Figure 3a–i respectively, decomposed into the individual time samples. In all of these plots we can see clear motion back and forth along the diffusion coordinate as time progresses. Considering both the plots here and in the Supplementary Materials we note that the spread of the sampled points along the respective normal mode coordinates for each value of the diffusion coordinate is similar (about 2 mass-frequency scaled normal mode units) indicating that the wavepacket is fairly well localised during the course of the dynamics. This spread is consistent among both the modes which are relatively well correlated with the diffusion coordinate, and those that are not. Considering the best correlated modes (1A’, 10A’ and 11A’) we see that the overall total magnitude of the motion along these modes, as reflected in the range between the maximum and minimum sampled normal mode coordinates, is largest for these modes (5 units for mode 1A’, 6 for 10A’ and 4 for 11A’), whilst for the less correlated modes the overall magnitude of the motion is less than or equal to 3.5 normal mode units. The spread of the wavepacket makes up a much larger proportion of the overall motion in the less correlated modes, indicating that these modes are less important in the dynamics, although there is clear motion along each of them for the duration of the dynamics (as there is along modes 1A’, 10A’ and 11A’). We thus find that although the 9D salicylaldimine dynamics can be decomposed into a single diffusion coordinate, this is no longer simply related to a single normal mode, but to multiple modes.

**Figure 4:**
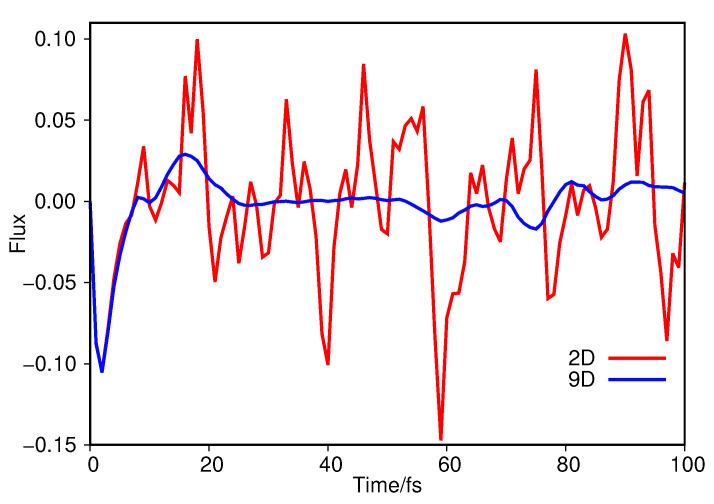
Wavepacket flux over the hydrogen transfer transition barrier in salicylaldimine as a function of propagation time. The solid, red line is the flux seen during dynamics in 2 dimensions whilst the dashed, blue line is that given when using the 9 normal modes listed in the text.

We can further see this by examining the flow of the wavepacket across the transition barrier between the keto and imine tautomers; this can be measured by evaluating the expectation value of a flux operator [6], at every timestep, represented by a surface perpendicular to mode 1A’ located at the peak of the transition barrier. In Figure 4 we plot the expectation value of the flux operator as a function of propagation time for both the 2D (Section 3.1.1) and 9D calculations on salicylaldimine.

Looking first at the flux across the proton-transfer barrier when using just two normal modes, it is clear that the wavepacket moves back and forth across the barrier for the duration of the dynamics. This clearly matches the picture given by the diffusion map in Figure 1 and Figure 2; the overall dynamics is dominated by motion along the 1A’ transition mode. Turning next to the 9D flux, we see an initial match between the flux in this case and that in 2D, with a negative flux indicating wavepacket motion from the imine side of the barrier to the keto. The flux approaches zero just before 10 fs and then we see motion back towards the imine side up to 20 fs. However, after this time there is little wavepacket flux over the transition barrier compared to that seen in the 2D case. This is indicative of the wavepacket motion transferring into other modes, after the initial movement along 1A’, due to inter-mode coupling. From the results of the diffusion map the coupling appears to be strongly into mode 10A’, which is backed up by looking at the position expectation values of the wavepacket along each mode; the largest magnitude motion is indeed along 10A’, going from an initial $\langle q_{10}\rangle =0$ to a minimum of $\langle q_{10}\rangle =-2.87$ after 38 fs.

### 3.2. Dynamics of Ethene

As a more complex example of the application of DM, we consider the non-adiabatic dynamics of ethene using the KRR-based direct-dynamics MCTDH (DD-MCTDH) method. The geometry of ethene was optimised using the state-averaged complete active space self-consistent field method in Molpro (version 2015.1.35), refs. [61,68,69] with the averaging being over all three electronic states (equally weighted) represented when using an active space comprising two electrons in two orbitals (SA(3)-CASSCF(2,2)). The 6-31G(d,p) basis set was used in these calculations. A frequency calculation was performed at the resulting geometry to provide the normal modes and frequencies needed to construct the coordinate system used in the dynamics, both in an 8D subset and the full 12D case. All energies were calculated using Molpro (versions 2015.1.35 and 2015.1.44). We discuss the 8D dynamics first.

#### 3.2.1. 8D Ethene

The initial 8D study of ethene used all normal modes except those which are best described as C-H stretches of various combinations (the highest frequency modes). The symmetry labels (in the D${}_{2h}$ point group) of the modes are shown in Table 4 along with their frequencies at the level of electronic structure theory used here.

The 8D dynamics of ethene was performed using the DVR bases shown in Table 4 along the eight mass-frequency scaled normal modes on all three electronic states. The initial wavefunction was constructed as an 8D Gaussian function of width $1/\sqrt{2}$, centred at the origin of the coordinate system (the Franck-Condon geometry) and placed on the first excited state (with B${}_{2u}$ symmetry). The wavepacket was propagated for 100 fs using the default ABM integrator for MCTDH. Four 2D combined modes were used: {1B${}_{2u}$,6B${}_{1u}$}, {2B${}_{2g}$,4B${}_{3g}$}, {5A${}_{g}$,7A${}_{g}$}, {3B${}_{3u}$,8A${}_{u}$}. 9 SPFs were used for each 2D combined mode to expand the wavefunction on the ground and second excited state (both of A${}_{g}$ symmetry) whilst 15 SPFs each were used on the initial state.

To construct the PES, 100 coordinates were sampled every 1 fs around the centre of the wavefunction on each state, and energies added to the database if the KRR variance at the selected geometry exceeded $10^{-3}$. Adiabatic energies for all three states were calculated at the SA(3)-CASSCF(2,2)/6-31G(d,p) level before being diabatised using the Procrustes diabatisation scheme. In addition to the KRR variance being used to determine which points to add to the database, an energy threshold of 20 eV was also used, such that if the calculated ground state energy exceeded this value it was rejected. In addition, before carrying out an electronic structure calculation, the KRR predicted energy was evaluated there and if found to be greater than 22 eV (a 10% discrepancy in the threshold to allow for errors in the KRR extrapolation of the PES), the point was rejected before expending time calculating the energy. The KRR fit of the diabatic energies and couplings was achieved using the additive kernel (Equation (13)) with α=0.01 along each mode. A total of 493 points were retained in the database compiled over the course of the dynamics. Finally, a secondary SVD fitting of the KRR PES was performed, with enough terms being retained to keep $||R||<10^{-3}\;E_{h}$. The number of terms in this expansion varied during the dynamics, depending on the number of sampled points in the KRR fit, but ranged from an initial 827, up to a maximum of 1421 before ending at 1387.

After propagation, the dynamics on the ground and initial excited states were separately analysed by DM. We used D=15 and $\beta_{\min}=0.2$. Here, 20 gridpoints were sampled every 10 fs and for the ground state analysis, the first sampling time was 10 fs to allow time for the wavepacket to begin transferring from the excited state.

**Figure 5:**
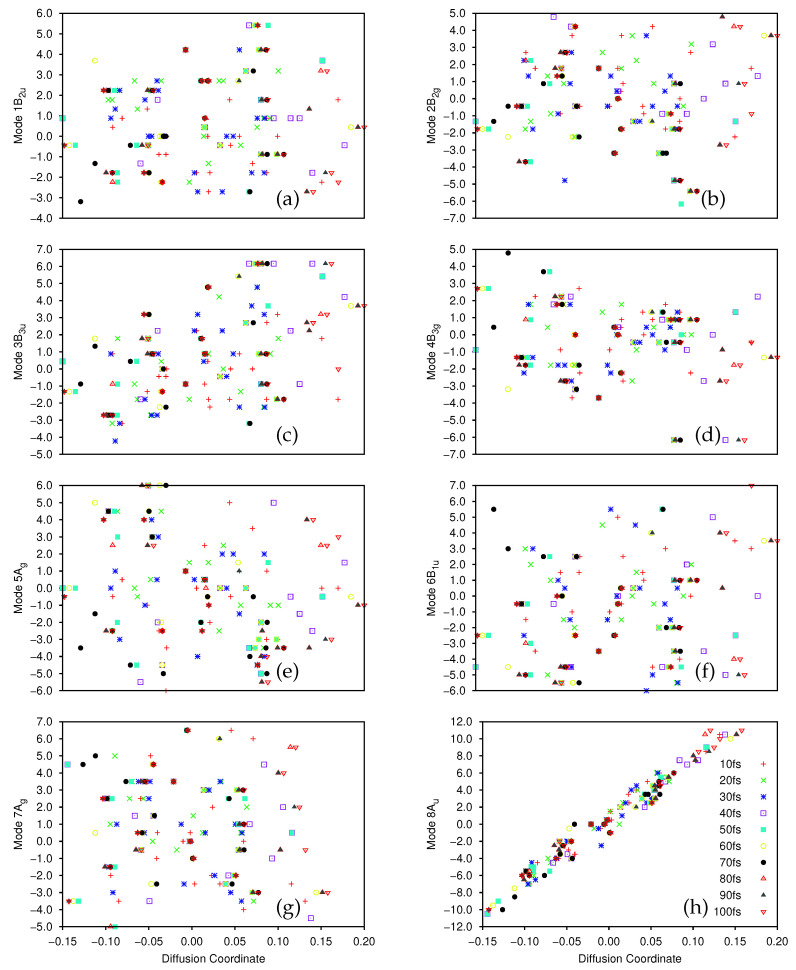
Normal mode coordinates of ethene, sampled on the ground, diabatic electronic state, plotted as functions of the diffusion coordinate: (**a**) Mode 1B${}_{2u}$; (**b**) Mode 2B${}_{2g}$; (**c**) Mode 3B${}_{3u}$; (**d**) Mode 4B${}_{3g}$; (**e**) Mode 5A${}_{g}$; (**f**) Mode 6B${}_{1u}$; (**g**) Mode 7A${}_{g}$; (**h**) Mode 8A${}_{u}$. In all cases data points are coloured according to the sample time; 20 gridpoints were sampled every 10 fs during the dynamics, the first sampling being after 10 fs. Key within plot (**h**) applies to all other plots.

**Figure 6:**
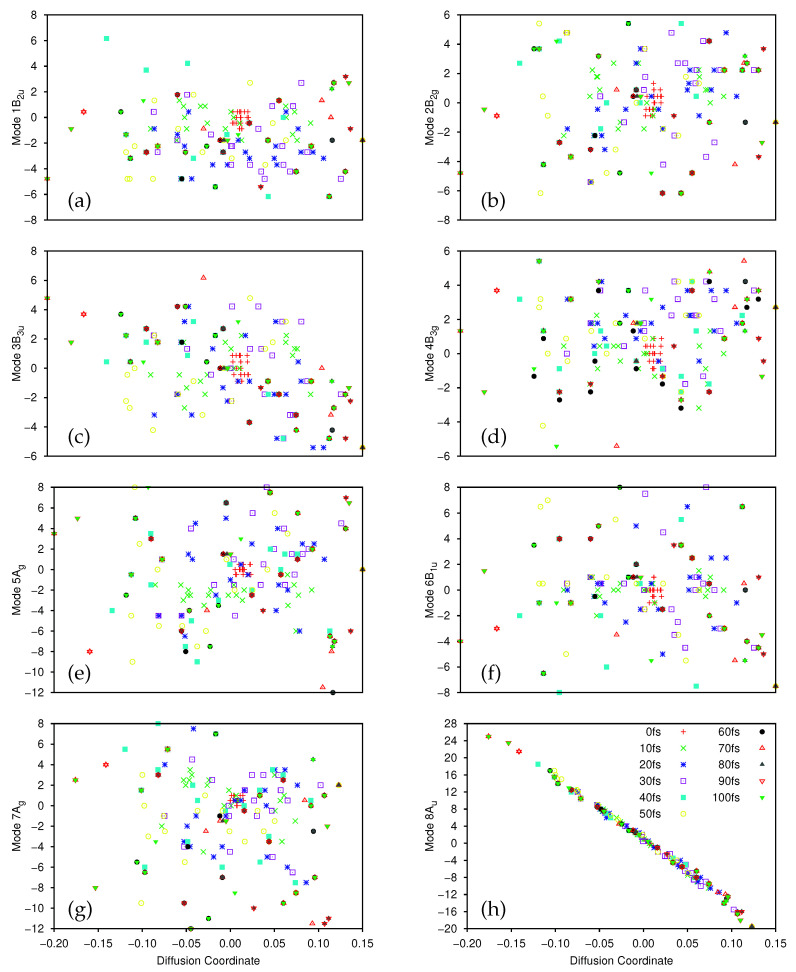
Normal mode coordinates of ethene, sampled on the first, diabatic electronic excited state, plotted as functions of the diffusion coordinate: (**a**) Mode 1B${}_{2u}$; (**b**) Mode 2B${}_{2g}$; (**c**) Mode 3B${}_{3u}$; (**d**) Mode 4B${}_{3g}$; (**e**) Mode 5A${}_{g}$; (**f**) Mode 6B${}_{1u}$; (**g**) Mode 7A${}_{g}$; (**h**) Mode 8A${}_{u}$. In all cases data points are coloured according to the sample time; 20 gridpoints were sampled every 10 fs during the dynamics. Key within plot (**h**) applies to all other plots.

In Figure 5 and Figure 6 we present plots of the results of the DM analysis, showing the correlation between the diffusion coordinates on the ground (Figure 5) and first excited (Figure 6) electronic states and the coordinates of the associated sampled points along the eight normal modes (mass-frequency scaled). We note that the three highest, non-trivial eigenvalues are: for the ground state, 0.235, 0.106 and 0.0881; for the excited state motion, 0.651, 0.370 and 0.233. Again we see a significant spectral gap between the two largest eigenvalues indicating that the overall motion of ethene in both states, after excitation from and subsequent non-radiative transfer back to the ground state, is dominated by a single diffusion coordinate. Looking at the eight plots in both figures, it is clear that the diffusion coordinate is dominated by the 8A${}_{u}$ normal mode (i.e., it correlates best with the diffusion coordinate) on both states; this mode is essentially a torsion mode, with the two CH${}_{2}$ groups rotating in opposite directions. All other normal modes show little or no correlation with the diffusion coordinate. Such torsional motion is expected to dominate for ethene, the excitation corresponding to a breaking of the ethene π-bond hence allowing free rotation around the C-C bond i.e., giving a *cis*-*trans* isomerization motion. So, we see that the diffusion map method has successfully extracted the most important mode from the large number of dimensions being followed in the wavepacket dynamics.

We note that the gradient of the plots containing the 8A${}_{u}$ normal mode and the diffusion coordinate differ for both states (Figure 5h and Figure 6h), the ground state diffusion coordinate having positive correlation with the 8A${}_{u}$ normal mode coordinate while the correlation is negative for the excited state. This is due to the fact that the diffusion coordinate is defined by the elements of an eigenvector of the diffusion operator matrix, but the overall sign of the eigenvectors of linear operators is arbitrary, hence so is the correlation of the diffusion coordinate with the normal mode.

To see the time-dependence of the diffusion maps more clearly, the data in Figure 5 and Figure 6 is presented in the Supplementary Materials with the sampled points from each timestep having their own plot (Figures S12–S19 in the Supplementary Materials correspond to those in Figure 5, whilst Figures S20–S27 therein represent the data from Figure 6). Considering the eight plots in Figure 6 (as well as parts (a) of Figures S20–S27), describing the dynamics on the excited state, we see the compactness of the initial wavefunction (the cluster of red crosses around the origin in all cases) before the wavefunction begins to move. In all cases we see a spreading of the sampled points over time, which begins soon after excitation. In the case of the 8A${}_{u}$ mode this spread is in both directions along the line of correlation between the normal mode and diffusion coordinates, whilst for the other modes the spread of the sampled points is less uniform. For the 1B${}_{2u}$ mode (Figure 6 and Figure S20) the sampled points spread along the diffusion coordinate initially before expanding along the normal mode coordinate up to 40 fs before partially contracting back. A similar delay in the spread of the sampled points along the normal mode is seen for mode 6B${}_{1u}$ (Figure 6f and Figure S25), the first major motion being seen after 20 fs, however, in this case we see no contraction at later times. For mode 4B${}_{3g}$ the initial wavepacket expansion is followed by a definite motion along the normal mode coordinate between 20 and 40 fs; a full dispersion along the diffusion coordinate is only seen after 70 fs. Considering mode 5A*g* (Figure 6e and Figure S24), the initial spread of the sampled points is dominated that along the normal mode coordinate with the diffusion coordinate spread coming more slowly. For the remaining modes (2B${}_{2g}$, 3B${}_{3u}$ and 7A${}_{g}$) the spreading of the sampled points in the diffusion map appears to occur in both directions simultaneously. Returning to the ground state (Figure 5 and Figures S12–S19 in the Supplementary Materials) we see that the distribution of points is wider to begin with because we only sampled points from 10 fs onwards where the wavepacket on the initially populated excited state had already had time to spread. This indicates that population transfer between the diabatic states is occurring over a wide region of space rather than at localised points. In all modes there is a contraction of distribution of the sampled points along the diffusion coordinate up to 30 fs before a rebound to the full range of the diffusion coordinate In mode 8A${}_{u}$ the initial spread of the sampled points follows the line of correlation seen in Figure 5h whereas for the other modes there is no initial correlation. Further on the subject of population transfer, we note that there was relatively little between the diabatic states over the 100 fs course of the dynamics, the vast majority being over the first 5 fs to the both ground and second excited states; at most 1.5% of the wavepacket transferred to the ground state whilst up to 4.2% went to the upper state.

**Figure 7:**
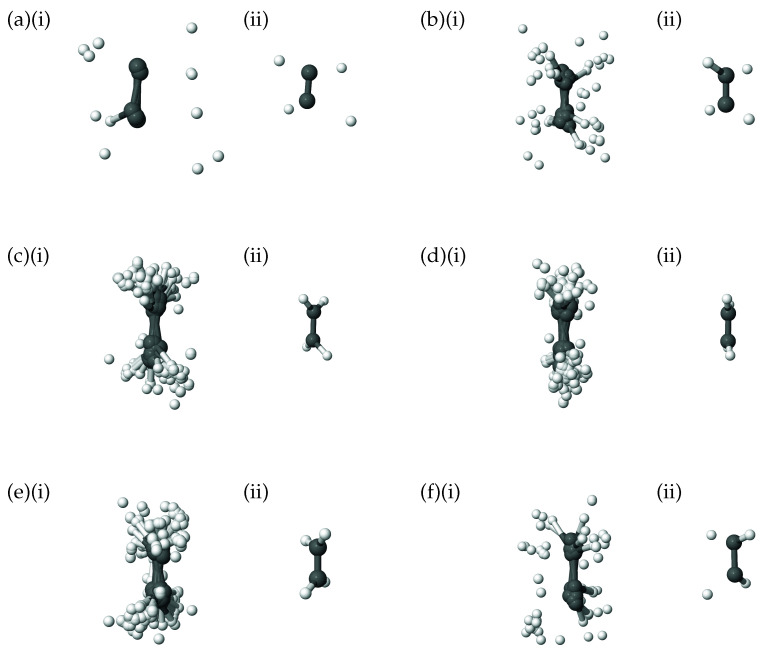
Geometries of salicylaldimine grouped according to their associated diffusion coordinate, $\theta_{2}$: (**a**) $-0.18<\theta_{2}<-0.13$; (**b**) $-0.13\leq \theta_{2}<-0.08$; (**c**) $-0.08\leq \theta_{2}<-0.03$; (**d**) $-0.03\leq \theta_{2}<0.03$; (**e**) $0.03\leq \theta_{2}<0.08$; (**f**) $0.08\leq \theta_{2}<0.13$. In each case plot (i) represents all sampled geometries within the diffusion coordinate domain, whilst plot (ii) is the average of the sampled geometries in the corresponding plot (i).

In addition to the plots showing the correlation of the 8A${}_{u}$ normal mode with the diffusion coordinate in ethene, in Figure 7 we show pictures of the ethene geometries, sampled during the diffusion map analysis, grouped according to their diffusion coordinates. In all six diagrams, (i) shows all sampled geometries, superimposed on one another, whilst, for clarity, (ii) gives the average of those geometries. It is apparent that, as we move across the diffusion-coordinate range from $-0.18<\theta_{2}<-0.13$ (Figure 7a) to that of $0.08\leq \theta_{2}<0.13$ (Figure 7f), the angle between the two CH${}_{2}$ groups reduces, going through geometries close to planar when $-0.03\leq \theta_{2}<0.03$ (Figure 7d) before increasing again in the opposite orientation to that in Figure 7a. This is further confirmation that the diffusion coordinate can mainly be characterised as a torsion mode, closely related to normal mode 8A${}_{u}$. Each of the panels in Figure 7 shows many different geometries corresponding to the motion across all normal modes except 8A${}_{u}$; these generate a spread of geometries around the important rotation about the C-C bond. This range corresponds to the uncorrelated spread of geometries sampled along all modes except 8A${}_{u}$, as seen in Figure 6.

At this point we can compare our results to those of Virshup et al. [52] where, because they used individual trajectories rather than a grid of basis functions as we are here, they were able to define a diffusion reaction coordinate (DRC) by integrating the Jacobian between the diffusion coordinate and real space coordinates, and then follow selected trajectories along the DRC over time. The previous work by Virshup et al., noted that the pyramidalisation of one of the CH${}_{2}$ groups followed the initial torsion motion resulting after electronic excitation; indeed, there is a minimum-energy conical intersection found at such a twisted-pyramidalised geometry resulting from such a torsion-pyramidalisation mechanism. The previously-generated DRC includes this path, and on further to hydrogen migration. In contrast, we see no clear sign of this pyramidalisation in Figure 5 and Figure 6, nor in Figure 7; this is conceivably due to limitations in our coordinate system, the use of normal modes perhaps being too restrictive to observe the pyramidalisation (let alone the hydrogen migration). To further analyse this we can consider the calculated eigenvectors of the diffusion operator with lower eigenvalues i.e., $\theta_{3}$, $\theta_{4}$, $\theta_{5}$ and $\theta_{6}$. For these eigenvectors we find that $\theta_{3}$ and $\theta_{6}$ are correlated with mode 8A${}_{u}$ (albeit more weakly than with $\theta_{2}$) whilst $\theta_{4}$ and $\theta_{5}$ are weakly correlated with modes 7A${}_{g}$ (C-C stretch with hydrogens moving in the opposite direction to their associated carbon) and 5A${}_{g}$ (C-C stretch with all hydrogens moving in concert with their bonded carbons), respectively. In none of the five considered eigenvectors do we see correlation with either mode 2B${}_{2g}$ (out-of-plane motion of all hydrogens in opposite directions at each end of the molecule) or 3B${}_{3u}$ (out-of-plane bending of all hydrogens in the same direction), which might be considered likely to contribute to the pyramidalisation motion. A different coordinate system may allow such motion to be observed; this conjecture will have to await further implementation to check. As a further check on the dynamics of ethene we also performed calculations in the full-dimensional space of normal modes, which we consider in the next section.

#### 3.2.2. 12D Ethene

Having noted the lack of pyramidalisation in the 8D dynamics of ethene, we finally consider 12D dynamics (i.e., including the four hydrogen stretching modes neglected in Section 3.2.1) in order to see whether the inclusion of these modes reveals the pyramidalisation.

The same level of electronic structure, and hence optimised geometry and normal modes were used as in the 8D calculations. Information about the DVR bases and mode frequencies are shown in Table 5. The KRR and SVD sampling and fitting during the dynamics were also as described in Section 3.2.1, the additional four modes being treated in the same way as the original eight. The initial wavefunction was a Gaussian function of width $1/\sqrt{2}$ in each DOF, centred at the Franck-Condon point on the first excited state. During the course of the dynamics 688 energies were added to the database, and there were a total of 2854 terms in the secondary SVD fit at the end of the wavepacket propagation. The normal modes were combined into six combined modes for the purpose of the time-dependent (SPF) basis, these being: {1B${}_{2u}$,2B${}_{2g}$}; {3B${}_{3u}$,4B${}_{3g}$}; {5A${}_{g}$,6B${}_{1u}$}; {7A${}_{g}$,10A${}_{g}$}; {8A${}_{u}$}; {9B${}_{1u}$,11B${}_{3g}$,12B${}_{2u}$}. For all combined modes, 5 SPFs were used on the first excited state, whilst a single SPF was used for each of the other two states. A small number of SPFs was necessary to limit memory demands on the computer hardware used, so as a result the propagation is far from converged with respect to the time-dependent basis. It is, however, worth illustrating, with future calculations in mind, whether we can gain useful information about the important DOFs from a poorly converged, high-dimensional calculation, as this will allow subsequent, lower-dimensional calculations to focus on the important modes, and hence to approach convergence.

After the dynamics propagation, the wavepacket on the ground and first excited state was analysed using the DM method; 20 points were sampled every 10 fs (the first 10 fs were skipped for the ground state to allow time for population transfer), and we used D=15 for the first excited state, D=10 for the ground-state, and $\beta_{\min}=0.2$ in all cases. The largest five non-trivial eigenvalues of the diffusion operator were, for the ground state, 0.420, 0.232, 0.180, 0.162 and 0.152, while for the excited state they were 0.480, 0.322, 0.214, 0.204 and 0.154.

**Figure 8:**
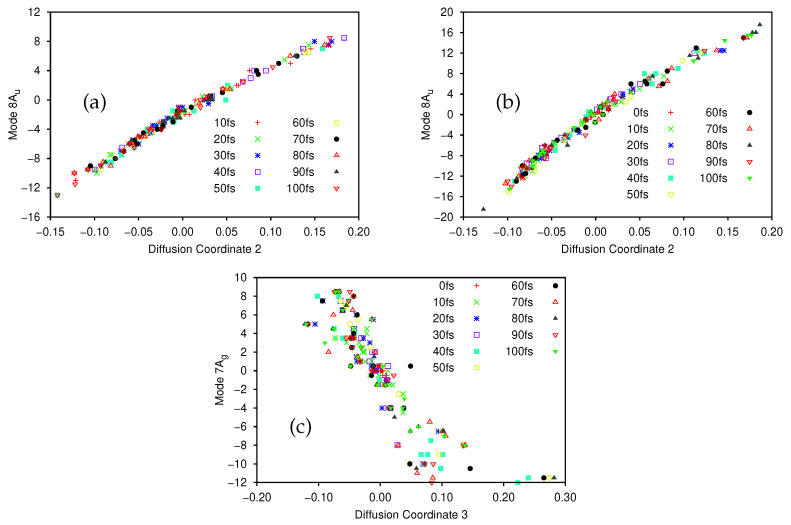
Sampled normal mode coordinates of ethene plotted as a function of the diffusion coordinates resulting from 12D dynamics. (**a**) 8A${}_{u}$ normal mode coordinates as a function of diffusion coordinate $\theta_{2}$ on the ground electronic state. (**b**) 8A${}_{u}$ normal mode coordinates as a function of diffusion coordinate $\theta_{2}$ on the first excited electronic state. (**c**) 7A${}_{g}$ normal mode coordinates as a function of diffusion coordinate $\theta_{3}$ on the first excited electronic state. In all cases data points are coloured according to the sample time; 20 gridpoints were sampled every 10 fs during the dynamics, the first sampling on the ground state being after 10 fs.

There is a clear spectral gap for the ground state, indicating that the dynamics on that state can be well represented by a single diffusion coordinate $\theta_{2}$. In Figure 8a we show the correlation between the sampled 8A${}_{u}$ normal mode coordinates and their corresponding diffusion coordinates. The spectral gap is less clear in the excited state case, where the $\theta_{3}$ eigenvector appears to play a more significant role in the dynamics. In Figure 8b we show the plot of the relationship between the sampled 8A${}_{u}$ normal mode coordinates and the $\theta_{2}$ coordinate on the excited state; again we see a very clear correlation between the two. In Figure 8c we show the correlation between the 7A${}_{g}$ mode and $\theta_{3}$ (which is even clearer if the points with $0.2<\theta_{3}<0.3$ are ignored. As noted in Section 3.2.1, 7A${}_{g}$ is a C-C stretching mode, so even here we see that there is little sign of pyramidalisation. Looking further at $\theta_{4}$ (mixture of 8A${}_{u}$ and 10A${}_{g}$), and $\theta_{5}$ and $\theta_{6}$ (both 8A${}_{u}$) on the excited state we see no clear evidence of pyramidalisation whilst, on the ground state, the lower eigenvalue diffusion coordinates correlate with 11B${}_{3g}$ ($\theta_{3}$), 8A${}_{u}$/9B${}_{1u}$ ($\theta_{4}$), 12B${}_{2u}$ ($\theta_{5}$) and 10A${}_{g}$ ($\theta_{6}$) (i.e., all four hydrogen stretching modes are involved along with the torsion, rather than any other out-of-plane motion). It is clear, however, that even with a few SPFs, using all 12 normal modes of ethene, we see the same torsion mode dominating the dynamics as with the better converged 8D calculation; the diffusion map can thus be used to glean useful information.

As with all previous calculations we provide, in the Supplementary Materials, deconstructed versions of the plots in Figure 8 (Figures S28–S30 corresponding to Figure 8a–c respectively) to help clarify the time evolution of the diffusion map. In much the same way as was seen for the 8D calculation, the sampled points on the excited state (Figure 8b,c, and Figures S29 and S30) are initially tightly clustered around the origin, before they spread out along the lines of correlation between the normal mode and diffusion coordinates. On the ground state (Figure 8a and Figure S28) the initial set of sampled points is already well distributed, reflecting the fact that the initial sampling was at 10 fs, after the wavepacket had had time to evolve away from the Franck-Condon point. Of potential interest are the small clusters of points locted at the top right of the plots, most easily seen in Figure S28 at times of 20, 30, 50, 60, 80 and 90 fs, which suggests a splitting of the wavepacket along the torsion mode, although the splitting is less clear at intermediate times.

Taking the analysis further, we performed a calculation to locate the twisted-pyramidalised conical intersection (TPCI) between the ground and first excited states, before using the vctrans program in the Quantics package to transform the Cartesian coordinates of the TPCI into the space of the mass-frequency scaled normal modes used for the dynamics. The results of this calculation are shown in Table 6, from which it is clear that the largest magnitude components are in the C-H stretching modes (9B${}_{1u}$, 10A${}_{g}$, 11B${}_{3g}$ and 12B${}_{2u}$), the largest distortion outside these four modes being along the 8A${}_{u}$ torsion mode, whose importance is thus confirmed. We saw that the one of the four C-H stretching modes (10A${}_{g}$) made an appearance in the $\theta_{4}$ diffusion coordinate on the excited state, whilst all four appeared in the lower eigenvalue diffusion coordinates on the ground state. Therefore, whilst the dominant diffusion coordinates in the dynamics corresponded to the torsion mode, there is evidence of motion in the C-H stretching modes, which are important to describe the pyramidalisation, in the less significant diffusion coordinates.

A shortcoming of our initial dynamics calculation is also apparent by comparing Table 5 and Table 6, where we note that the TPCI coordinate is not actually included in the domain of the DVR grid with the grids along modes 9B${}_{3g}$, 11B${}_{3g}$ and 12B${}_{2u}$ all being too short. As such a second DD-MCTDH calculation was performed using longer, but less dense for reasons of computational cost, DVR grids which are given in Table 7 with the frequencies being the same as those in Table 5. The initial conditions and KRR sampling for the calculation were as for the initial calculation, but the calculation was only run for 90 fs. 665 points were added to the energy database whilst the number of terms in the SVD fit of the PES was 3417 at the end.

Diffusion map analysis was carried out on the results of the second dynamics calculation for the ground and first exited states using the same parameters as for the first calculation. For the ground state the largest five, non-trivial eigenvalues of the diffusion operator were 0.163, 0.147, 0.136, 0.131 and 0.0926, and for the excited state they were 0.157, 0.137, 0.0932, 0.0897 and 0.0693. In both cases there is no clear spectral gap in the eigenvalues suggesting that the motion of the molecule cannot be reduced to a single mode as was the case for the original calculations; this may be due to the lack of convergence of the dynamics because of the reduction in the density of the gridpoints, such that the gap is not observed. The diffusion map analysis was repeated with lower values of *D* in an attempt to locate a spectral gap in line with the results in Table 3, but none was found, therefore we have to consider at least the five diffusion coordinates with the largest eigenvalues as contributing to the dynamics.

On the excited state, diffusion coordinate $\theta_{2}$ correlates most strongly with mode 9B${}_{1u}$ and more weakly with 12B${}_{2u}$, both of which are C-H stretches, whilst torsion mode 8A${}_{u}$ correlates with $\theta_{3}$. Coordinate $\theta_{4}$ is weakly correlated with the three modes, 8A${}_{u}$, 9B${}_{1u}$ and 11B${}_{3g}$, and $\theta_{5}$ correlates with the C-C stretching mode, 7A${}_{g}$. The diffusion coordinate with lowest eigenvalue in this selection, $\theta_{5}$, is weakly correlated with modes 2B${}_{2g}$, 5A${}_{g}$ and 6B${}_{1u}$, none of which play a great role in reaching the TPCI, as is the case with $\theta_{5}$ and its mode, 7A${}_{g}$. However, we see that the three diffusion coordinates with the largest eigenvalues contain contributions from the torsion mode and three of the C-H stretch modes. The absence of 10A${}_{g}$ is surprising, but may be related to the poor convergence of the dynamics.

On the ground state, $\theta_{2}$ is correlated with modes 2B${}_{2g}$, 4B${}_{3g}$ and 8A${}_{u}$, whilst $\theta_{3}$ correlates most strongly with the torsion mode and less so with 4B${}_{3g}$ and 11B${}_{2g}$. C-H stretching mode 12B${}_{2u}$ correlates strongly with coordinate $\theta_{4}$, whilst $\theta_{5}$ has contributions from the three C-H stretches excluding 10A${}_{g}$, and, finally, $\theta_{6}$ is correlated with mode 7A${}_{g}$.

From the second calculation on 12D ethene we see from the diffusion map analysis, especially on the excited state, that the torsion mode and three of the C-H stretching modes are important, and these modes also appear in the diffusion coordinates on the ground state; this is indicative of some motion towards the TPCI. The picture is not clear due to the lack of a spectral gap, but we gain useful (if incomplete, with the missing 10A${}_{g}$) information about the dynamics towards the conical intersection, even with an unconverged calculation. Longer time propagations, in both the first and second calculations, may resolve more clearly the modes involved.

## 4. Discussion

In this work we have presented an implementation of a NLDR method, specifically DM, to study the grid-based nuclear quantum dynamics methods implemented in the Quantics package. The method has been tested on two many-dimensional molecular systems, salicylaldimine and ethene, both of which have been treated using direct-dynamics (although the methods discussed here can also be used where pre-fitted PESs have been used).

Salicylaldimine was studied initially; this is a system we have examined in the past, allowing us to check the results of the DM analysis compared to our previous understanding of the dynamics. The initial 2D study (using DD-SM) demonstrated the dominance of the hydrogen-transfer normal mode in the make up of the diffusion coordinate, as expected. We were also able to analyse the parameters used in the DM model to provide some guidance for future users as to their influence. Moving on to a 9D model of salicylaldimine, using MCTDH in this case, we were again able to see the hydrogen-transfer mode contributing to the dominant diffusion coordinate, but this time other normal modes played a significant role too. This was further confirmed by considering the flux of the wavepacket across the keto-imine barrier along the transfer mode, the magnitude of which was greatly reduced after the first 20 fs, indicating the conversion of motion across the barrier into that in other, orthogonal modes mediated by inter-mode coupling. The DM method thus provides clearer insight into the dynamics of salicylaldimine than the flux analysis alone.

Because much of our work is focussed on modelling the non-adiabatic dynamics of electronically excited molecules, the DM method has also been applied to wavepacket motion on excited electronic states (each of which was treated individually). We have studied two models of ethene, the initial nuclear wavepacket having been promoted to the first electronic excited state; first, an 8D system ignoring the four hydrogen stretching modes and, second, the full 12D system. In both of these cases, the wavepackets on the initial and ground states were analysed using DM; for both states, in both dimensionalities, the dominant diffusion coordinate can primarily be categorised as being a torsion mode corresponding to the 8A${}_{u}$ normal mode. This makes sense considering that the electronic excitation results in the severing of the ethene π-bond, allowing rotation about the C-C bond. However, earlier work on ethene using DMs by Virshup et al. [52] pointed out that the initial torsion should be followed by a pyramidalisation of one of the CH${}_{2}$ groups, but this was not seen here in either the 8D or first 12D cases, where the spectral gap in the diffusion map analysis implied a single dominant torsion motion. The further calculation, which located the TPCI, indicated that the inclusion of C-H stretching modes in the dynamics was vital as was extension of the DVR grids used in the first 12D calculation. The diffusion map analysis on the second 12D calculation indicated the importance of 3 of the C-H stretching modes along with the torsion mode; a better converged dynamics calculation may have clarified matters further. We also conjecture that the difficulty in seeing the pyramidalisation may be a limitation of our use of normal modes defined at the Franck-Condon point; use of valence coordinates, for example, may allow further insight at geometries far from this point (e.g., at dihedral angles around 90°), but we have not so far implemented such coordinates in our DD-GB methods, although we plan to do so shortly.

## 5. Conclusions

In conclusion, we have presented a useful addition to the suite of analysis methods applicable to grid-based dynamics, and we have demonstrated this approach in direct MCTDH simulations of many-dimensional molecular systems with multiple electronic states. Future work will include the implementation of the DM method for DD-vMCG, where the use of Gaussian basis functions may allow the use of DRCs, as previously implemented by Virshup et al., for AIMS. Further calculations aimed at a more thorough analysis of ethene isomerization are also in progress, in order to consolidate the different viewpoints given by grid-based and trajectory-based simulations of photodynamics.

The work presented here has focussed on using diffusion maps to determine the dominant dynamical pathways followed by the two molecules under consideration, but it may be true that important components of the dynamics are missed by such an approach. Consider, for example, an oscillating wavepacket, where a small part of the wavepacket moves over an energy barrier into a dissociative mode at some point during each oscillation; the method as presented here would not detect this dissociation. Future work will include modifying our method in an attempt to capture such motion by altering the sampling method; currently points are included in the sample if the wavefunction density there is greater than or equal to the parameter, $\beta_{\min}$, multiplied by the density at the gridpoint closest to the centre of the wavepacket, $\rho_{0}$, but we could introduce another parameter, $\beta_{\max}$, so that only gridpoints where the density is in the range $\beta_{\min}\rho_{0}\leq \rho \leq \beta_{\max}\rho_{0}$ are selected. It would be interesting to see whether such an approach can capture weaker features of the dynamics.

## Figures and Tables

**Table 1:** Details of the normal modes, discrete variable representation (DVR) bases and initial wavepacket conditions used in the salicylaldimine calculations. (^a^) Frequencies of the normal modes calculated at the proton transfer transition state. Ref. [67] (^b^) Type of underlying basis functions used to generate the DVR bases: sine denotes eigenfunctions of the 1D particle-in-a-box and HO refers to the eigenfunctions of the harmonic oscillator problem. (^c^) The number of DVR gridpoints for each mode. (^d^) Locations of the initial and final DVR gridpoints along each mode. Note that the sine DVR gridpoints are evenly spaced whilst the HO points are not, being more concentrated towards the origin. (^e^) Coordinate of the centre of the initial wavepacket. (^f^) Width of the initial wavepacket.

Mode	Freq. ^a^/cm${}^{-1}$	DVR ^b^	No. DVR ^c^	DVR Domain ^d^	WP Centre ^e^	WP Width ^f^
1A’	1512.48*i*	sine	101	[−10,10]	0.96	0.5706
5A”	423.75	HO	21	[−5.55,5.55]	0	0.7409
7A’	535.89	HO	21	[−5.55,5.55]	0	0.7150
9A’	630.30	HO	21	[−5.55,5.55]	0	0.7115
10A’	639.60	HO	21	[−5.55,5.55]	0	0.7745
11A’	791.82	HO	21	[−5.55,5.55]	0	0.7590
13A’	847.13	HO	21	[−5.55,5.55]	0	0.6902
32A’	1662.73	HO	21	[−5.55,5.55]	0	0.6707
36A’	2175.61	HO	21	[−5.55,5.55]	0.14	0.7704

**Table 2:** Effect on the largest non-trivial diffusion operator eigenvalues of increasing $\beta_{\min}$, the density threshold coefficient. $\epsilon_{2}$ is the largest non-trivial eigenvalue and $\epsilon_{3}$ is the second largest. The kernel distance scaling parameter was D=2 in all cases and the number of required gridpoint samples at each timestep was 10.

$\beta_{\min}$	$\epsilon_{2}$	$\epsilon_{3}$	$\epsilon_{3}/\epsilon_{2}$
0.1	0.640975	0.535722	0.836
0.2	0.629970	0.364329	0.578
0.3	0.388341	0.341085	0.878
0.5	0.235725	0.153087	0.649
0.8	0.115689	0.083850	0.725

**Table 3:** Effect of increasing *D* on the largest non-trivial diffusion operator. Here, $\epsilon_{2}$ is the largest non-trivial eigenvalue and $\epsilon_{3}$ the second largest. The density threshold coefficient, $\beta_{\min}$, was 0.2 in all cases and the number of required gridpoint samples at each timestep was 10.

*D*	$\epsilon_{2}$	$\epsilon_{3}$	$\epsilon_{3}/\epsilon_{2}$
0.5	0.987008	0.980774	0.994
1	0.923951	0.836574	0.905
2	0.629970	0.364329	0.578
3	0.351864	0.171568	0.488
5	0.134912	0.062413	0.463
10	0.033891	0.015604	0.460
20	0.008463	0.003900	0.460
50	0.001353	0.000624	0.461
100	0.000338	0.000156	0.462

**Table 4:** Details of the normal modes, DVR bases and initial wavepacket conditions used in the 8D ethene calculations. (^a^) Frequencies of the normal modes calculated at the minimum energy geometry of the electronic ground state. (^b^) Type of underlying basis functions used to generate the DVR bases: sine denotes eigenfunctions of the 1D particle-in-a-box and HO refers to the eigenfunctions of the harmonic oscillator problem. (^c^) Number of DVR gridpoints for each mode. (^d^) Locations of the initial and final DVR gridpoints along each mode. Note that the sine DVR gridpoints are evenly spaced whilst the HO points are not, being more concentrated towards the origin.

Mode	Freq. ^a^/cm${}^{-1}$	DVR ^b^	No. DVR ^c^	DVR Domain ^d^
1B${}_{2u}$	884.14	HO	25	[−6.164,6.164]
2B${}_{2g}$	1056.60	HO	25	[−6.164,6.164]
3B${}_{3u}$	1144.47	HO	25	[−6.164,6.164]
4B${}_{3g}$	1337.21	HO	25	[−6.164,6.164]
5A${}_{g}$	1439.57	sine	41	[−12.0,8.0]
6B${}_{1u}$	1592.97	sine	33	[−8.0,8.0]
7A${}_{g}$	1777.10	sine	41	[−12.0,8.0]
8A${}_{u}$	2164.29	sine	101	[−25.0,25.0]

**Table 5:** Details of the normal modes, DVR bases and initial wavepacket conditions used in the initial 12D ethene calculation. (^a^) Frequencies of the normal modes calculated at the minimum energy geometry of the electronic ground state. (^b^) Type of underlying basis functions used to generate the DVR bases: sine denotes eigenfunctions of the 1D particle-in-a-box and HO refers to the eigenfunctions of the harmonic oscillator problem. (^c^) Number of DVR gridpoints for each mode. (^d^) Locations of the initial and final DVR gridpoints along each mode. Note that the sine DVR gridpoints are evenly spaced whilst the HO points are not, being more concentrated towards the origin.

Mode	Freq. ^a^/cm${}^{-1}$	DVR ^b^	No. DVR ^c^	DVR Domain ^d^
1B${}_{2u}$	884.14	HO	25	[−6.164,6.164]
2B${}_{2g}$	1056.60	HO	21	[−5.550,5.550]
3B${}_{3u}$	1144.47	HO	25	[−6.164,6.164]
B${}_{3g}$	1337.21	HO	25	[−6.164,6.164]
5A${}_{g}$	1439.57	sine	41	[−12.0,8.0]
6B${}_{1u}$	1592.97	sine	37	[−8.0,8.0]
7A${}_{g}$	1777.10	sine	43	[−12.0,9.0]
8A${}_{u}$	2164.29	sine	101	[−25.0,25.0]
9B${}_{1u}$	3301.63	HO	25	[−6.164,6.164]
10A${}_{g}$	3322.07	sine	37	[−9.0,9.0]
11B${}_{3g}$	3383.71	HO	21	[−5.550,5.550]
12B${}_{2u}$	3409.48	HO	21	[−5.550,5.550]

**Table 6:** Dimensionless mass-frequency scale normal mode coordinates of the twisted-pyramidalised conical intersection. Mode frequencies given in Table 5.

Mode	Coordinate	Mode	Coordinate
1B${}_{2u}$	−2.58	7A${}_{g}$	2.61
2B${}_{2g}$	0.000116	8A${}_{u}$	5.94
3B${}_{3u}$	0.000116	9B${}_{1u}$	9.27
4B${}_{3g}$	−2.22	10A${}_{g}$	−8.99
5A${}_{g}$	−0.692	11B${}_{3g}$	6.47
6B${}_{1u}$	1.21	12B${}_{2u}$	−6.75

**Table 7:** Details of the normal modes, DVR bases and initial wavepacket conditions used in the second 12D ethene calculation, which includes the coordinate of the twisted-pyramidalised conical intersection. (^a^) Type of underlying basis functions used to generate the DVR bases: sine denotes eigenfunctions of the 1D particle-in-a-box and HO refers to the eigenfunctions of the harmonic oscillator problem. (^b^) Number of DVR gridpoints for each mode. (^c^) Locations of the initial and final DVR gridpoints along each mode. Note that the sine DVR gridpoints are evenly spaced whilst the HO points are not, being more concentrated towards the origin.

Mode	DVR ^a^	No. DVR ^b^	DVR Domain ^c^
1B${}_{2u}$	HO	21	[−5.55,5.55]
2B${}_{2g}$	HO	21	[−5.55,5.55]
3B${}_{3u}$	HO	21	[−5.55,5.55]
B${}_{3g}$	HO	21	[−5.55,5.55]
5A${}_{g}$	sine	21	[−12.0,8.0]
6B${}_{1u}$	sine	17	[−8.0,8.0]
7A${}_{g}$	sine	21	[−12.0,8.0]
8A${}_{u}$	sine	51	[−25.0,25.0]
9B${}_{1u}$	sine	31	[−15.0,15.0]
10A${}_{g}$	sine	31	[−15.0,15.0]
11B${}_{3g}$	sine	25	[−12.0,12.0]
12B${}_{2u}$	sine	25	[−12.0,12.0]

## Data Availability

Data can be found at wrap.warwick.ac.uk/159820. Computer code developed here is freely available as part of the Quantics package: www2.chem.ucl.ac.uk/quantics/.

## Supplementary Materials

The following are available online. Figure S1 shows a time-resolved version of the data in Figure 1a in the main paper. Figure S2 shows a time-resolved version of the data in Figure 1b. Figures S3–S11 show time-resolved versions of the data in Figure 3a–i respectively. Figures S12–S19 show time-resolved versions of the data in Figure 5a–h respectively. Figures S20–S27 show time-resolved versions of the data in Figure 6a–h respectively. Figure S28 shows a time-resolved version of the data in Figure 8a. Figure S29 shows a time-resolved version of the data in Figure 8b. Figure S30 shows a time-resolved version of the data in Figure 8c.
