# Supplementary material for: Analyzing Grid-Based Direct Quantum Molecular Dynamics Using Non-Linear Dimensionality Reduction
Source: Molecules. 2021 Dec 7;26(24):7418. doi: 10.3390/molecules26247418 (PMC8708769; doi:10.3390/molecules26247418)
Supplement: Supplementary file 1 [file molecules-26-07418-s001.zip › molecules-1465280-supplementary.pdf]

Supplementary Information: Analyzing Grid-Based Direct Quantum Molecular Dynamics Using Non-Linear Dimensionality Reduction

Gareth W. Richings and Scott Habershon

This document contains supplementary information to the above titled paper, in particular it contains deconstructed versions of Figures 1, 3, 5, 6 and 8 in that work *i.e.* those figures contain time-dependent data overlaid in single plots, whereas here each timestep is given its own figure. In this document:

- Fig. S1 shows a time-resolved version of the data in Fig. 1(a) in the main paper.
- Fig. S2 shows a time-resolved version of the data in Fig. 1(b).
- Figs. S3, S4, S5, S6, S7, S8, S9, S10 and S11 show time-resolved versions of the data in Figs. 3(a)-(i) respectively.
- Figs. S12, S12, S13, S14, S15, S16, S17, S18 and S19 show time-resolved versions of the data in Figs. 5(a)-(h) respectively.
- Figs. S20, S20, S21, S22, S23, S24, S25, S26 and S27 show time-resolved versions of the data in Figs. 6(a)-(h) respectively.
- Fig. S28 shows a time-resolved version of the data in Fig. 8(a).
- Fig. S29 shows a time-resolved version of the data in Fig. 8(b).
- Fig. S30 shows a time-resolved version of the data in Fig. 8(c).

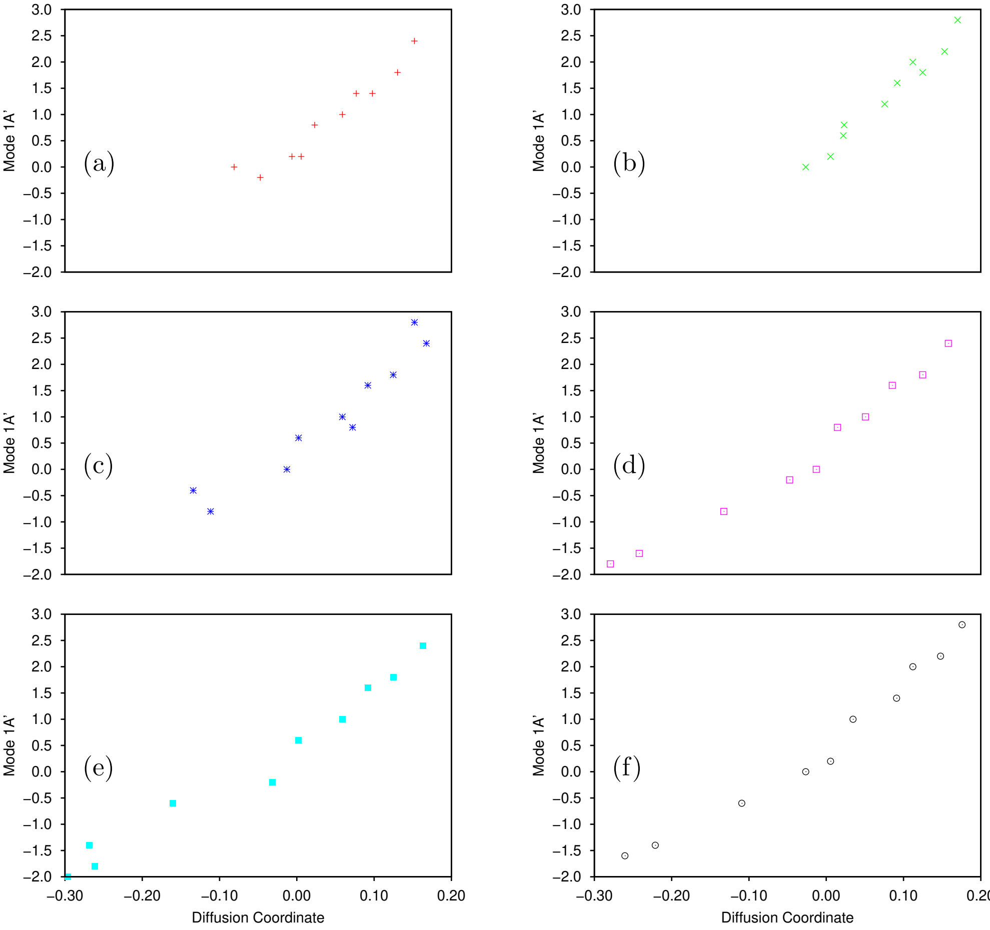

Figure S1: Sampled 1A' normal mode coordinates of salicylaldimine plotted as functions of the diffusion coordinate, with each plot representing a different sampling time: **(a)** 0 fs; **(b)** 20 fs; **(c)** 40 fs; **(d)** 60 fs; **(e)** 80 fs; **(f)** 100 fs. Data is the same as that presented in Figure 1(a) in the main paper for the 2D calculation.

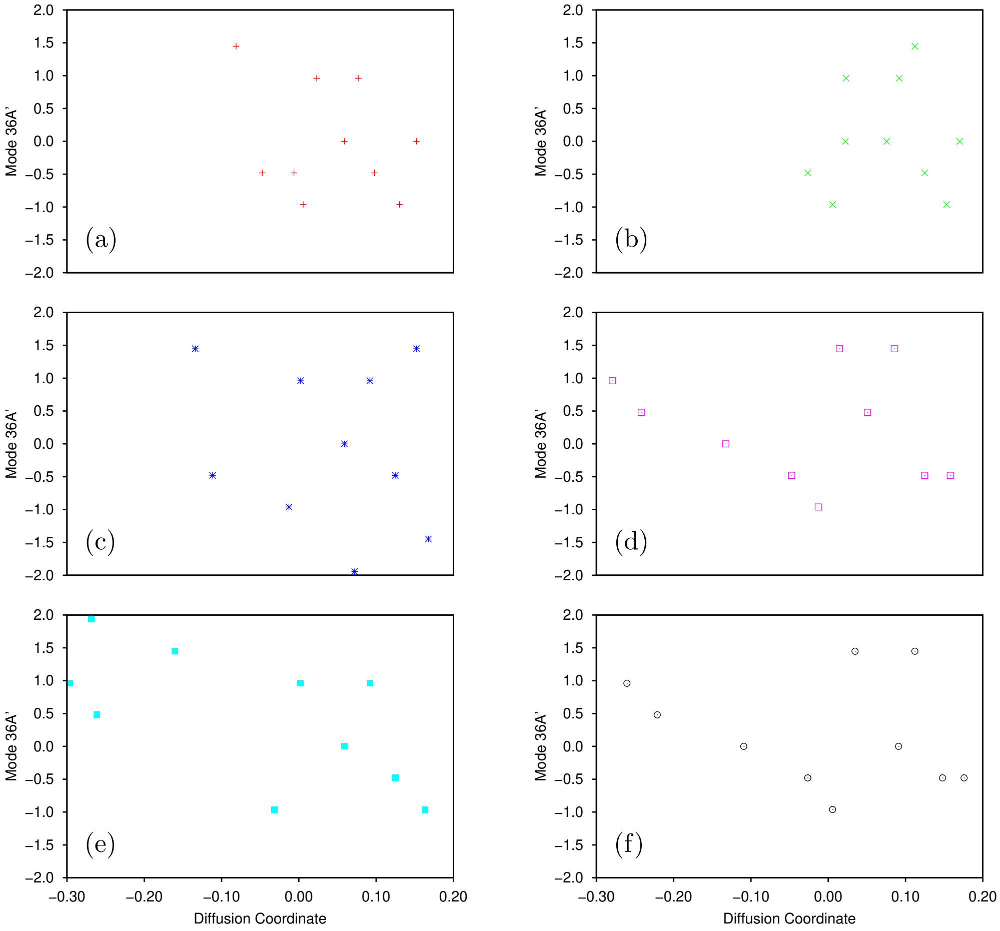

Figure S2: Sampled 36A' normal mode coordinates of salicylaldimine plotted as functions of the diffusion coordinate, with each plot representing a different sampling time: **(a)** 0 fs; **(b)** 20 fs; **(c)** 40 fs; **(d)** 60 fs; **(e)** 80 fs; **(f)** 100 fs. Data is the same as that presented in Figure 1(b) in the main paper for the 2D calculation.

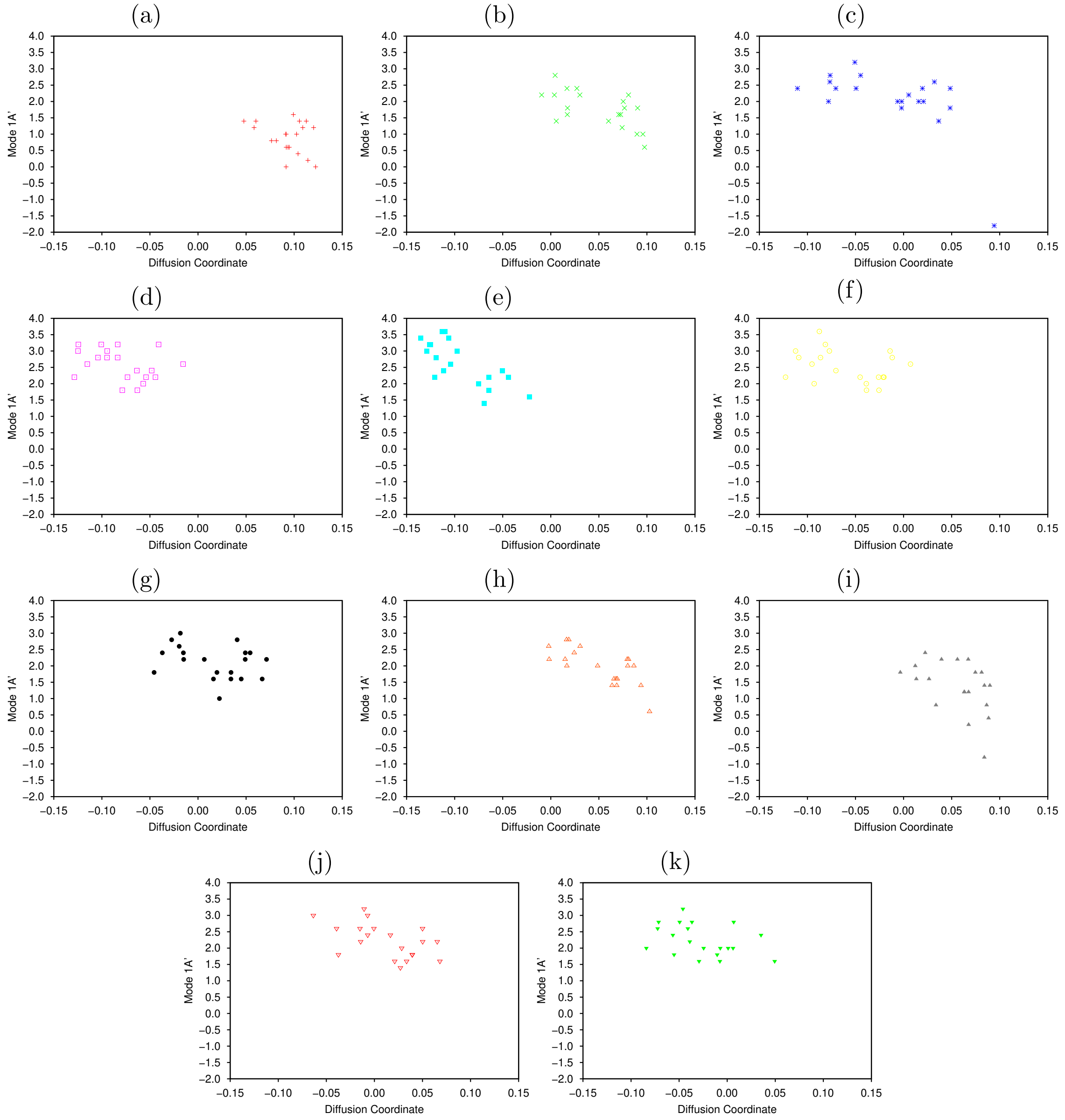

Figure S3: Sampled 1A' normal mode coordinates of salicylaldimine plotted as functions of the diffusion coordinate, with each plot representing a different sampling time: **(a)** 0 fs; **(b)** 10 fs; **(c)** 20 fs; **(d)** 30 fs; **(e)** 40 fs; **(f)** 50 fs; **(g)** 60 fs; **(h)** 70 fs; **(i)** 80 fs; **(j)** 90 fs; **(k)** 100 fs. Data is the same as that presented in Figure 3(a) in the main paper for the 9D calculation.

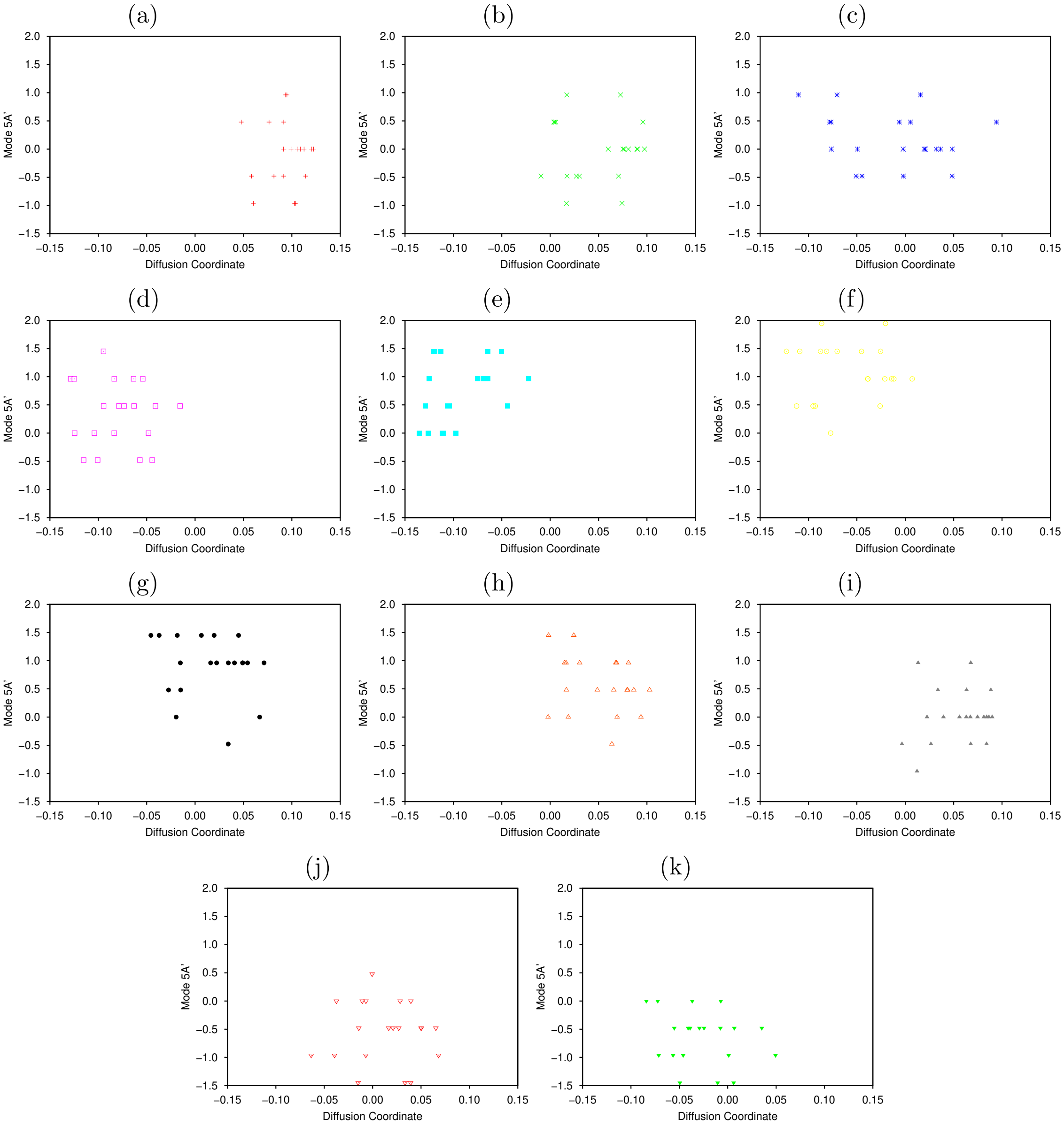

Figure S4: Sampled 5A' normal mode coordinates of salicylaldimine plotted as functions of the diffusion coordinate, with each plot representing a different sampling time: **(a)** 0 fs; **(b)** 10 fs; **(c)** 20 fs; **(d)** 30 fs; **(e)** 40 fs; **(f)** 50 fs; **(g)** 60 fs; **(h)** 70 fs; **(i)** 80 fs; **(j)** 90 fs; **(k)** 100 fs. Data is the same as that presented in Figure 3(b) in the main paper for the 9D calculation.

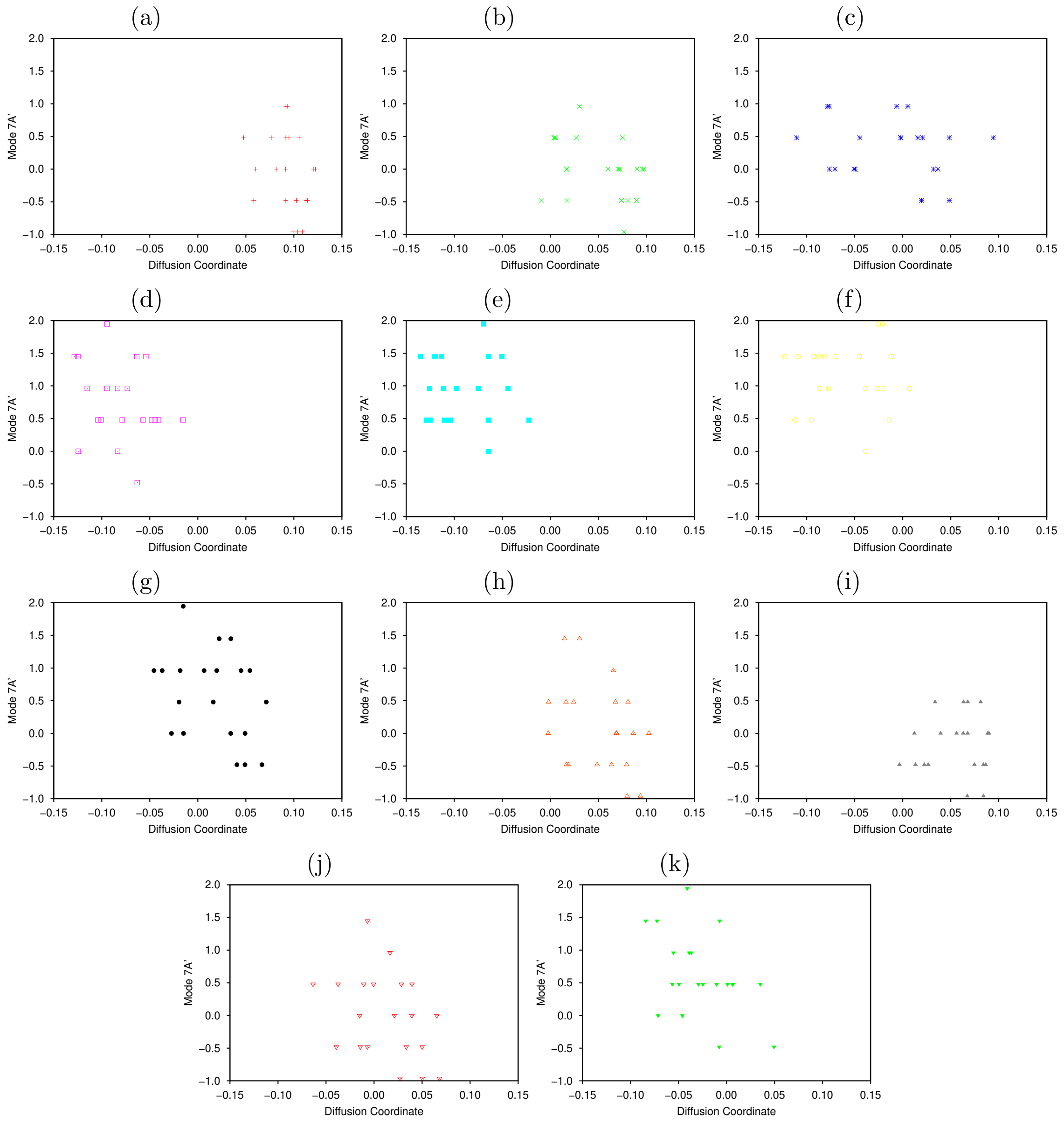

Figure S5: Sampled 7A' normal mode coordinates of salicylaldimine plotted as functions of the diffusion coordinate, with each plot representing a different sampling time: **(a)** 0 fs; **(b)** 10 fs; **(c)** 20 fs; **(d)** 30 fs; **(e)** 40 fs; **(f)** 50 fs; **(g)** 60 fs; **(h)** 70 fs; **(i)** 80 fs; **(j)** 90 fs; **(k)** 100 fs. Data is the same as that presented in Figure 3(c) in the main paper for the 9D calculation.

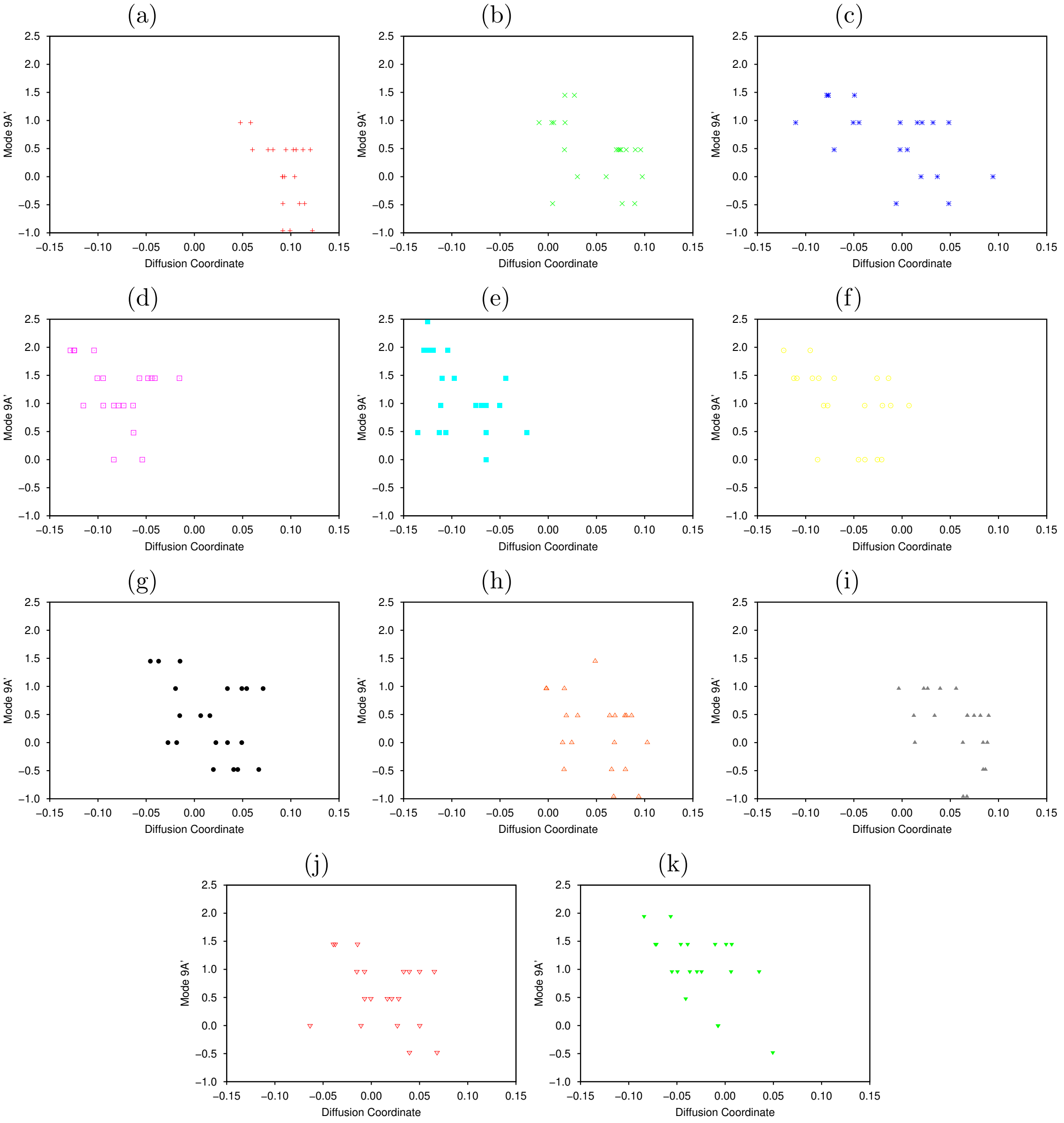

Figure S6: Sampled 9A' normal mode coordinates of salicylaldimine plotted as functions of the diffusion coordinate, with each plot representing a different sampling time: **(a)** 0 fs; **(b)** 10 fs; **(c)** 20 fs; **(d)** 30 fs; **(e)** 40 fs; **(f)** 50 fs; **(g)** 60 fs; **(h)** 70 fs; **(i)** 80 fs; **(j)** 90 fs; **(k)** 100 fs. Data is the same as that presented in Figure 3(d) in the main paper for the 9D calculation.

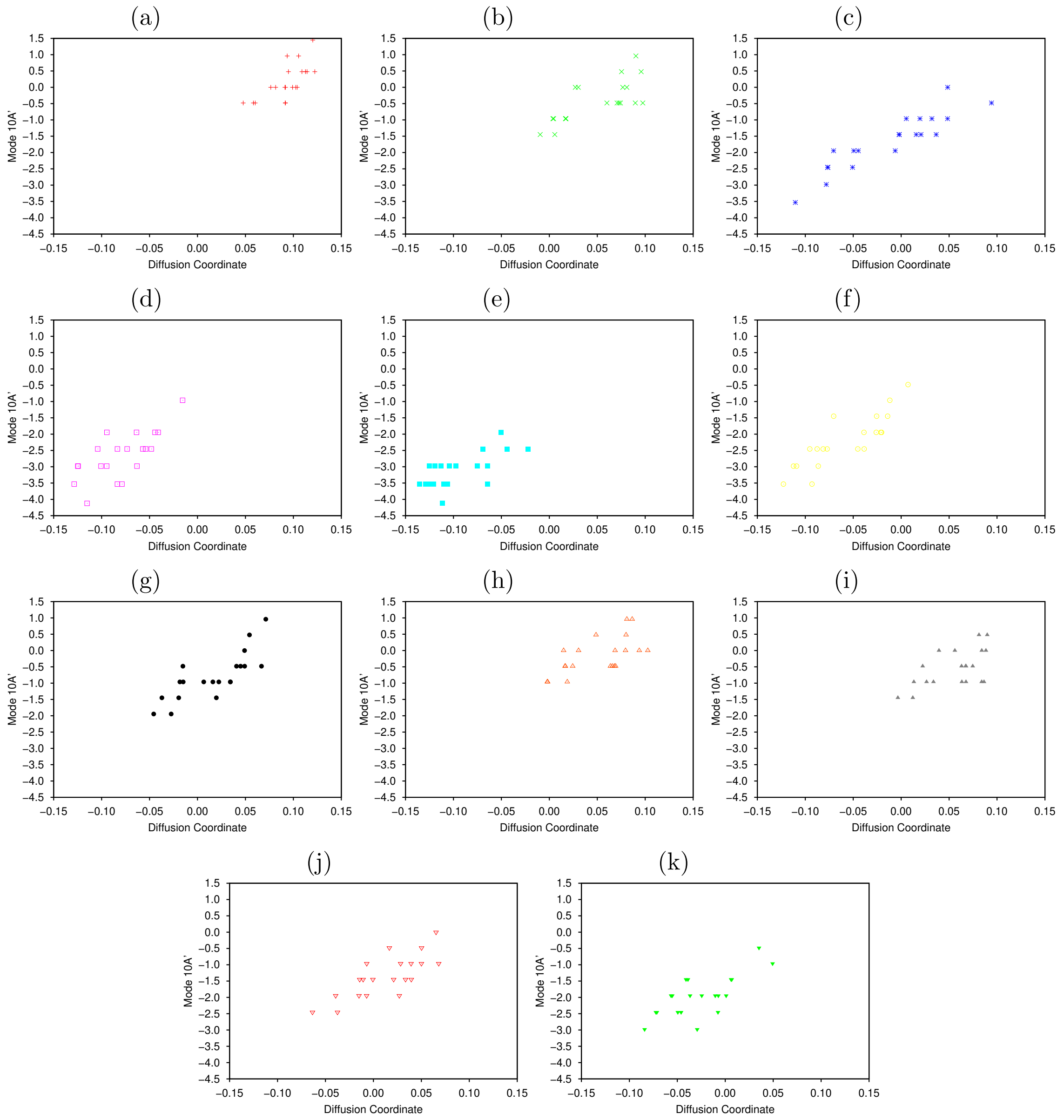

Figure S7: Sampled 10A' normal mode coordinates of salicylaldimine plotted as functions of the diffusion coordinate, with each plot representing a different sampling time: **(a)** 0 fs; **(b)** 10 fs; **(c)** 20 fs; **(d)** 30 fs; **(e)** 40 fs; **(f)** 50 fs; **(g)** 60 fs; **(h)** 70 fs; **(i)** 80 fs; **(j)** 90 fs; **(k)** 100 fs. Data is the same as that presented in Figure 3(e) in the main paper for the 9D calculation.

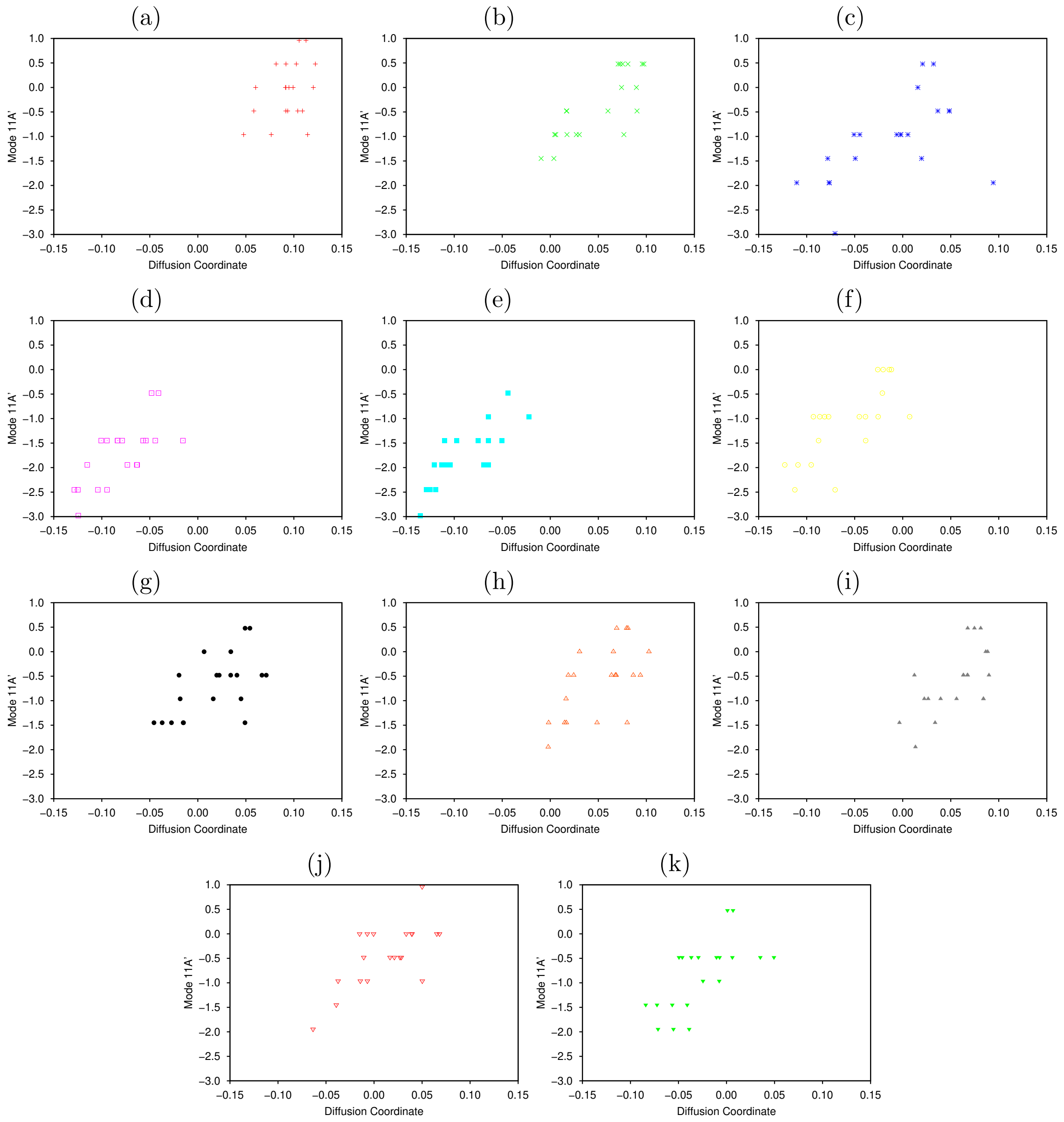

Figure S8: Sampled 11A' normal mode coordinates of salicylaldimine plotted as functions of the diffusion coordinate, with each plot representing a different sampling time: **(a)** 0 fs; **(b)** 10 fs; **(c)** 20 fs; **(d)** 30 fs; **(e)** 40 fs; **(f)** 50 fs; **(g)** 60 fs; **(h)** 70 fs; **(i)** 80 fs; **(j)** 90 fs; **(k)** 100 fs. Data is the same as that presented in Figure 3(f) in the main paper for the 9D calculation.

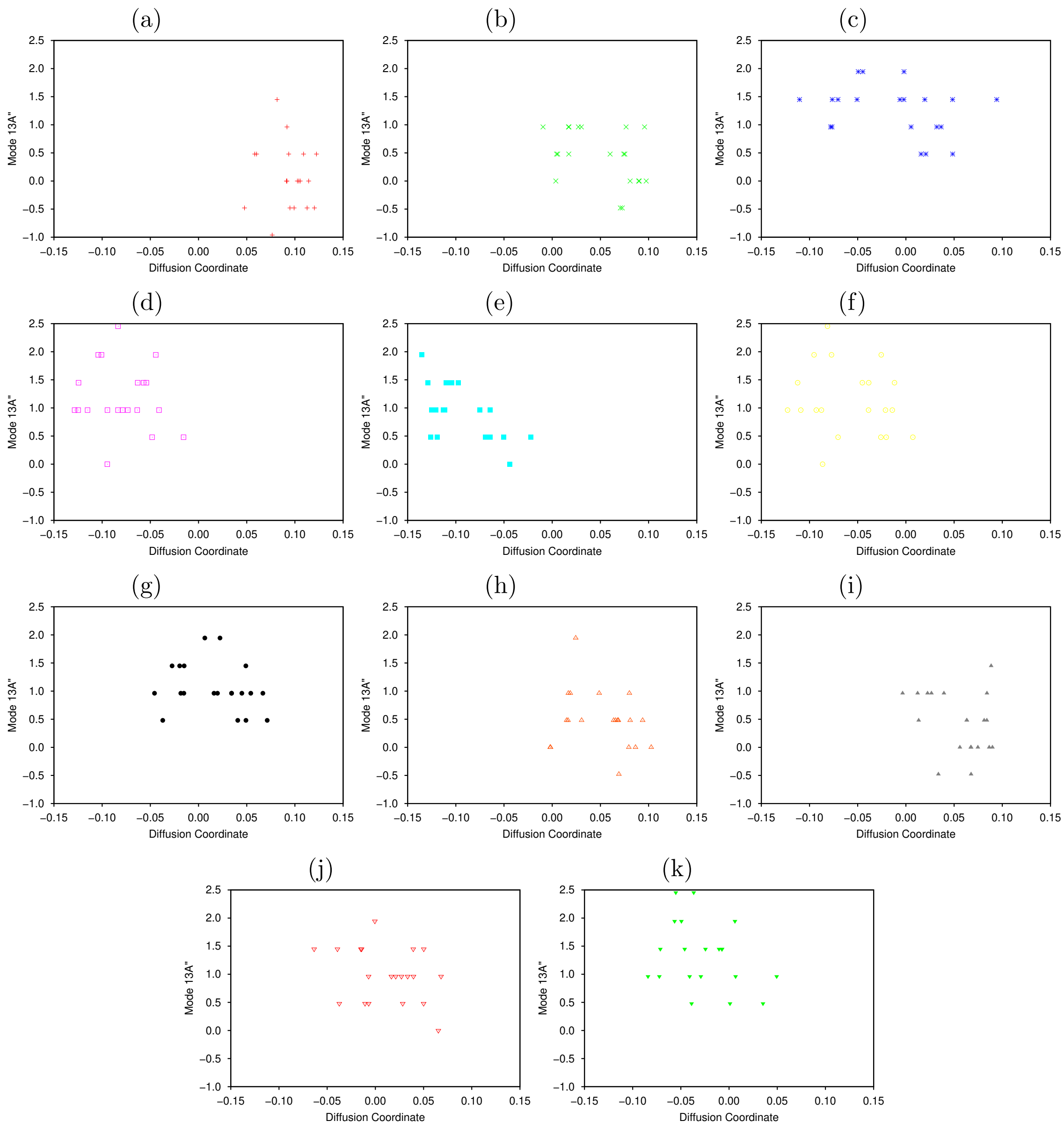

Figure S9: Sampled 13A" normal mode coordinates of salicylaldimine plotted as functions of the diffusion coordinate, with each plot representing a different sampling time: **(a)** 0 fs; **(b)** 10 fs; **(c)** 20 fs; **(d)** 30 fs; **(e)** 40 fs; **(f)** 50 fs; **(g)** 60 fs; **(h)** 70 fs; **(i)** 80 fs; **(j)** 90 fs; **(k)** 100 fs. Data is the same as that presented in Figure 3(g) in the main paper for the 9D calculation.

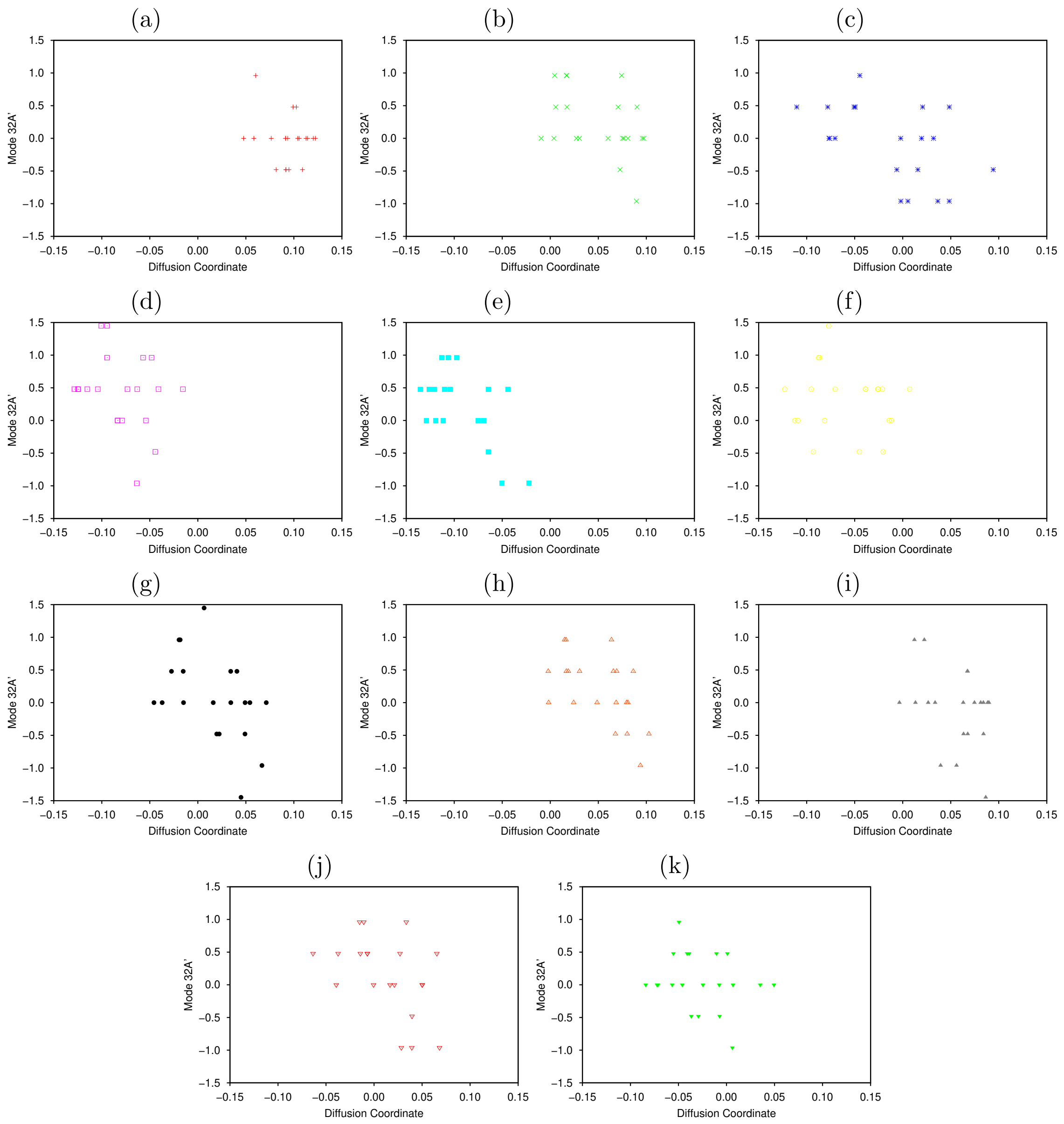

Figure S10: Sampled 32A' normal mode coordinates of salicylaldimine plotted as functions of the diffusion coordinate, with each plot representing a different sampling time: **(a)** 0 fs; **(b)** 10 fs; **(c)** 20 fs; **(d)** 30 fs; **(e)** 40 fs; **(f)** 50 fs; **(g)** 60 fs; **(h)** 70 fs; **(i)** 80 fs; **(j)** 90 fs; **(k)** 100 fs. Data is the same as that presented in Figure 3(i) in the main paper for the 9D calculation.

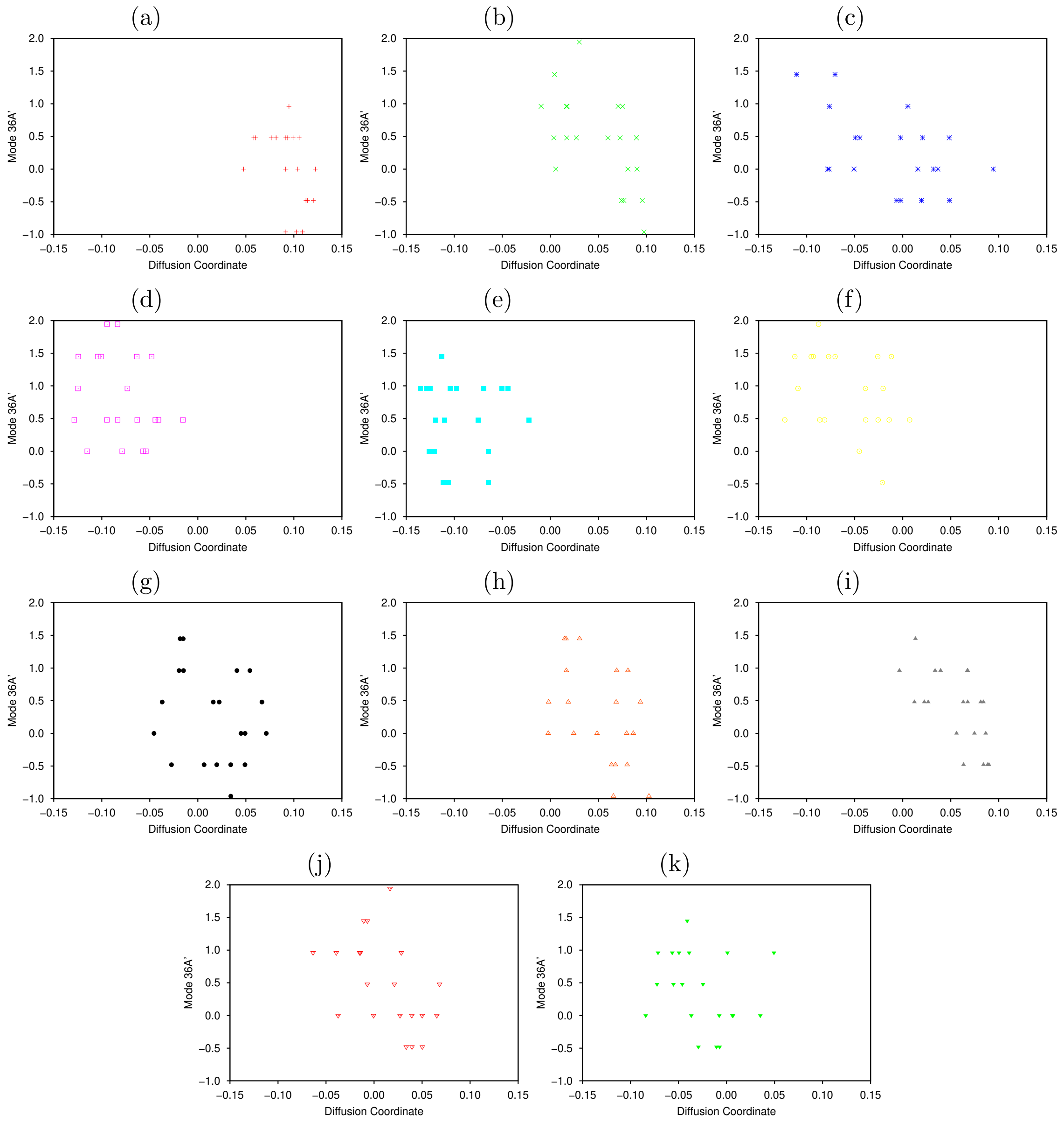

Figure S11: Sampled 36A' normal mode coordinates of salicylaldimine plotted as functions of the diffusion coordinate, with each plot representing a different sampling time: **(a)** 0 fs; **(b)** 10 fs; **(c)** 20 fs; **(d)** 30 fs; **(e)** 40 fs; **(f)** 50 fs; **(g)** 60 fs; **(h)** 70 fs; **(i)** 80 fs; **(j)** 90 fs; **(k)** 100 fs. Data is the same as that presented in Figure 3(i) in the main paper for the 9D calculation.

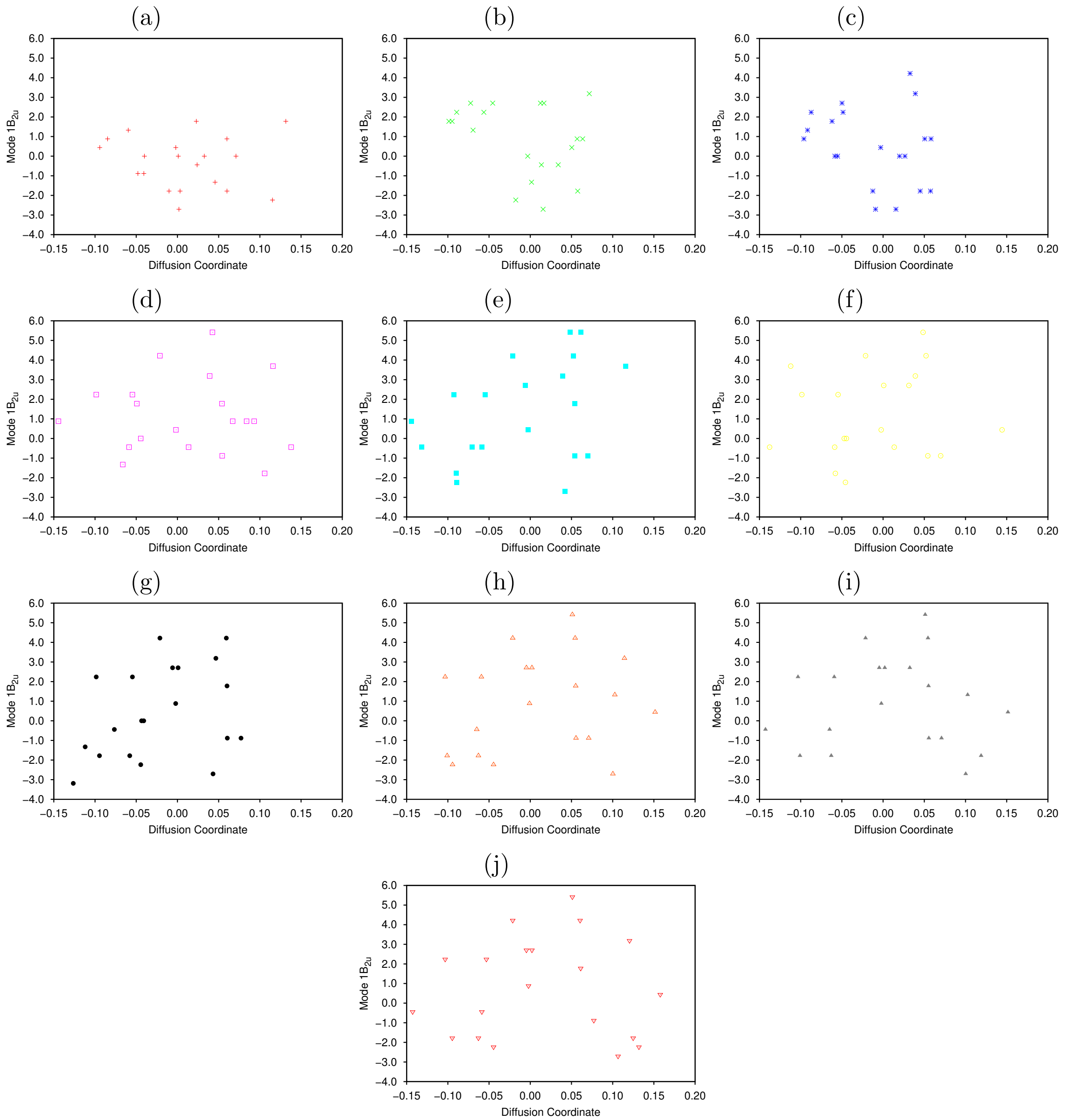

Figure S12: Sampled $1B_{2u}$ normal mode coordinates on the ground, diabatic state of ethene plotted as functions of the diffusion coordinate, with each plot representing a different sampling time: **(a)** 10 fs; **(b)** 20 fs; **(c)** 30 fs; **(d)** 40 fs; **(e)** 50 fs; **(f)** 60 fs; **(g)** 70 fs; **(h)** 80 fs; **(i)** 90 fs; **(j)** 100 fs. Data is the same as that presented in Figure 5(a) in the main paper for the 8D calculation.

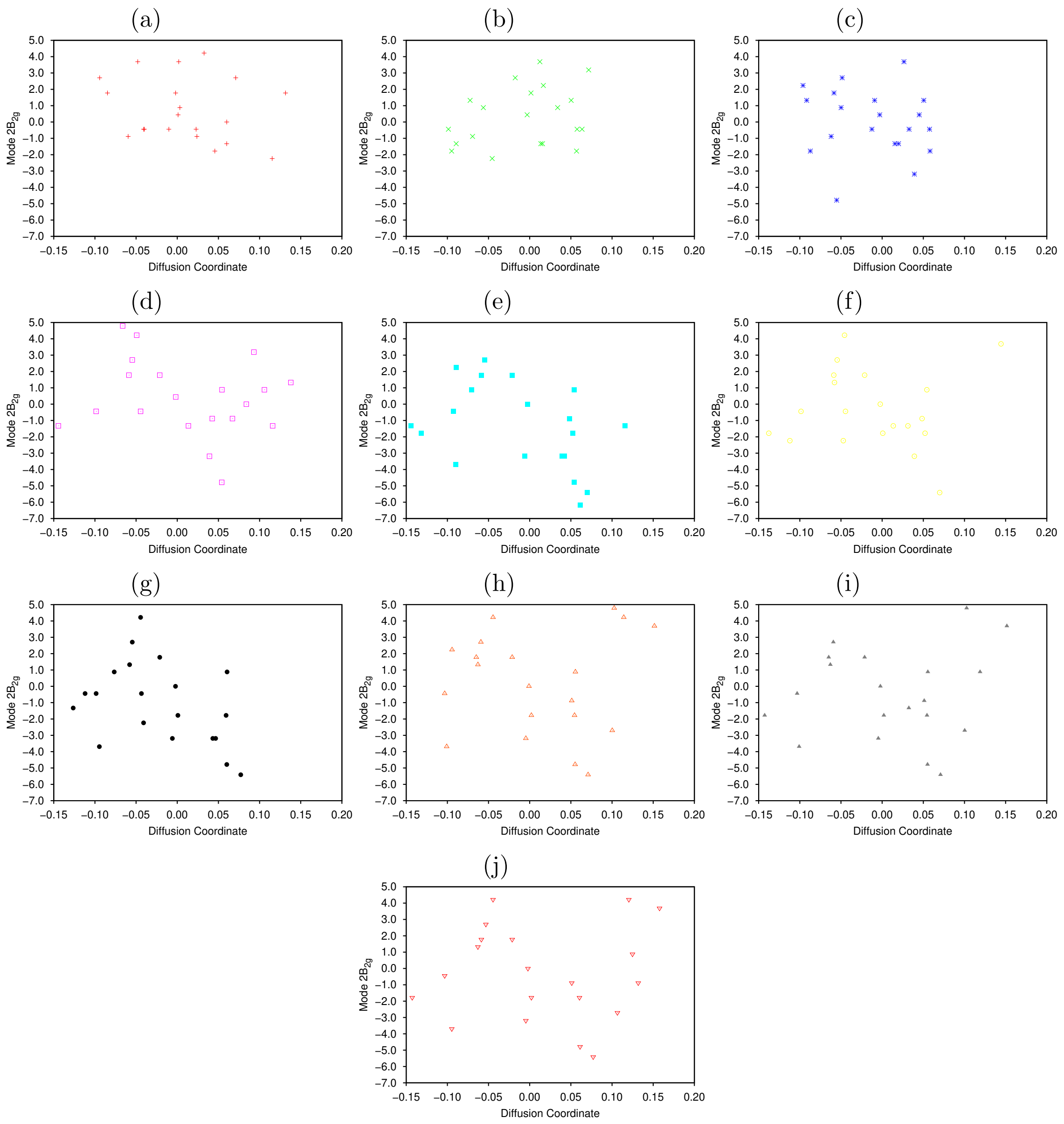

Figure S13: Sampled $2B_{2g}$ normal mode coordinates on the ground, diabatic state of ethene plotted as functions of the diffusion coordinate, with each plot representing a different sampling time: **(a)** 10 fs; **(b)** 20 fs; **(c)** 30 fs; **(d)** 40 fs; **(e)** 50 fs; **(f)** 60 fs; **(g)** 70 fs; **(h)** 80 fs; **(i)** 90 fs; **(j)** 100 fs. Data is the same as that presented in Figure 5(b) in the main paper for the 8D calculation.

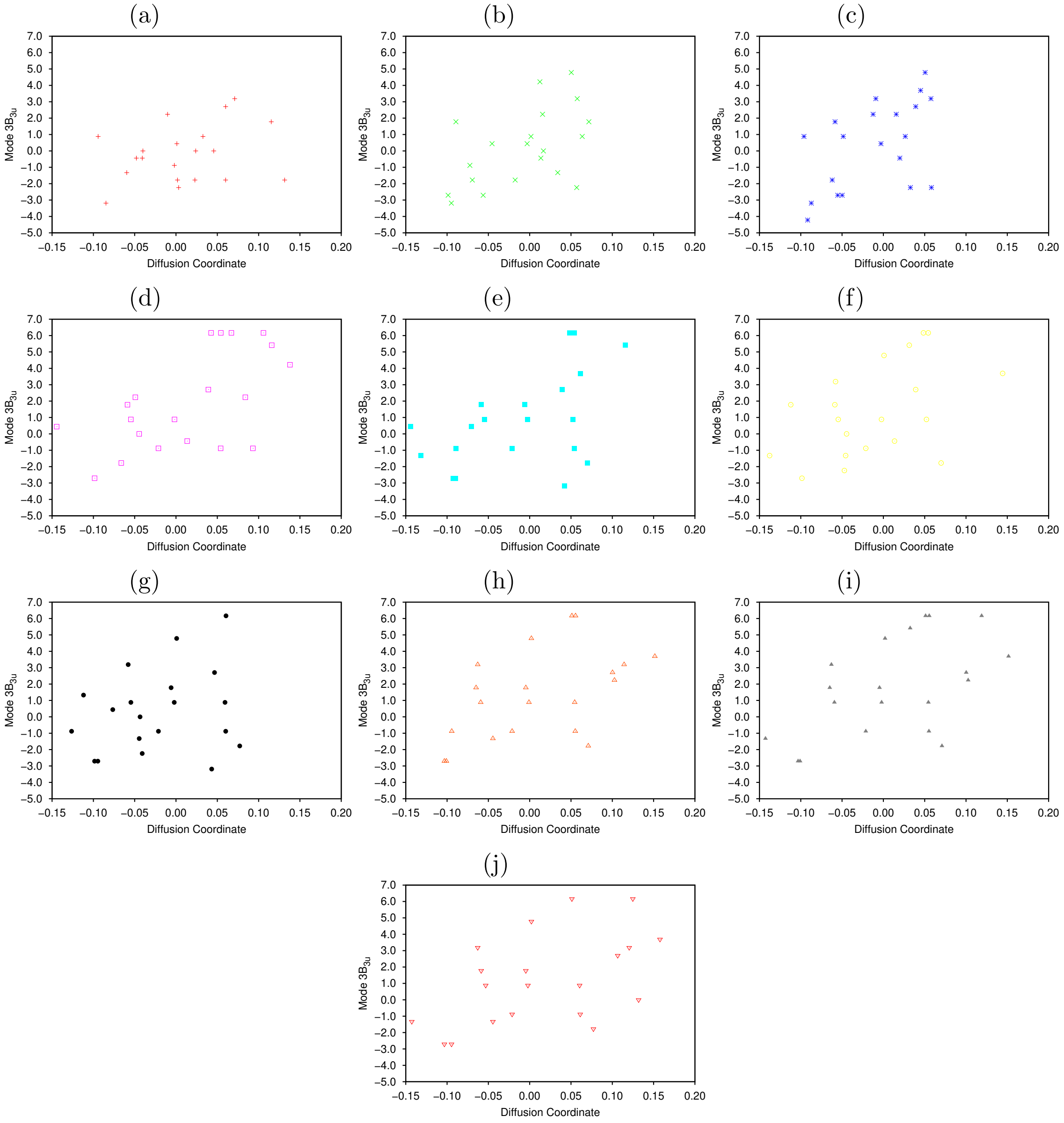

Figure S14: Sampled $3B_{3u}$ normal mode coordinates on the ground, diabatic state of ethene plotted as functions of the diffusion coordinate, with each plot representing a different sampling time: **(a)** 10 fs; **(b)** 20 fs; **(c)** 30 fs; **(d)** 40 fs; **(e)** 50 fs; **(f)** 60 fs; **(g)** 70 fs; **(h)** 80 fs; **(i)** 90 fs; **(j)** 100 fs. Data is the same as that presented in Figure 5(c) in the main paper for the 8D calculation.

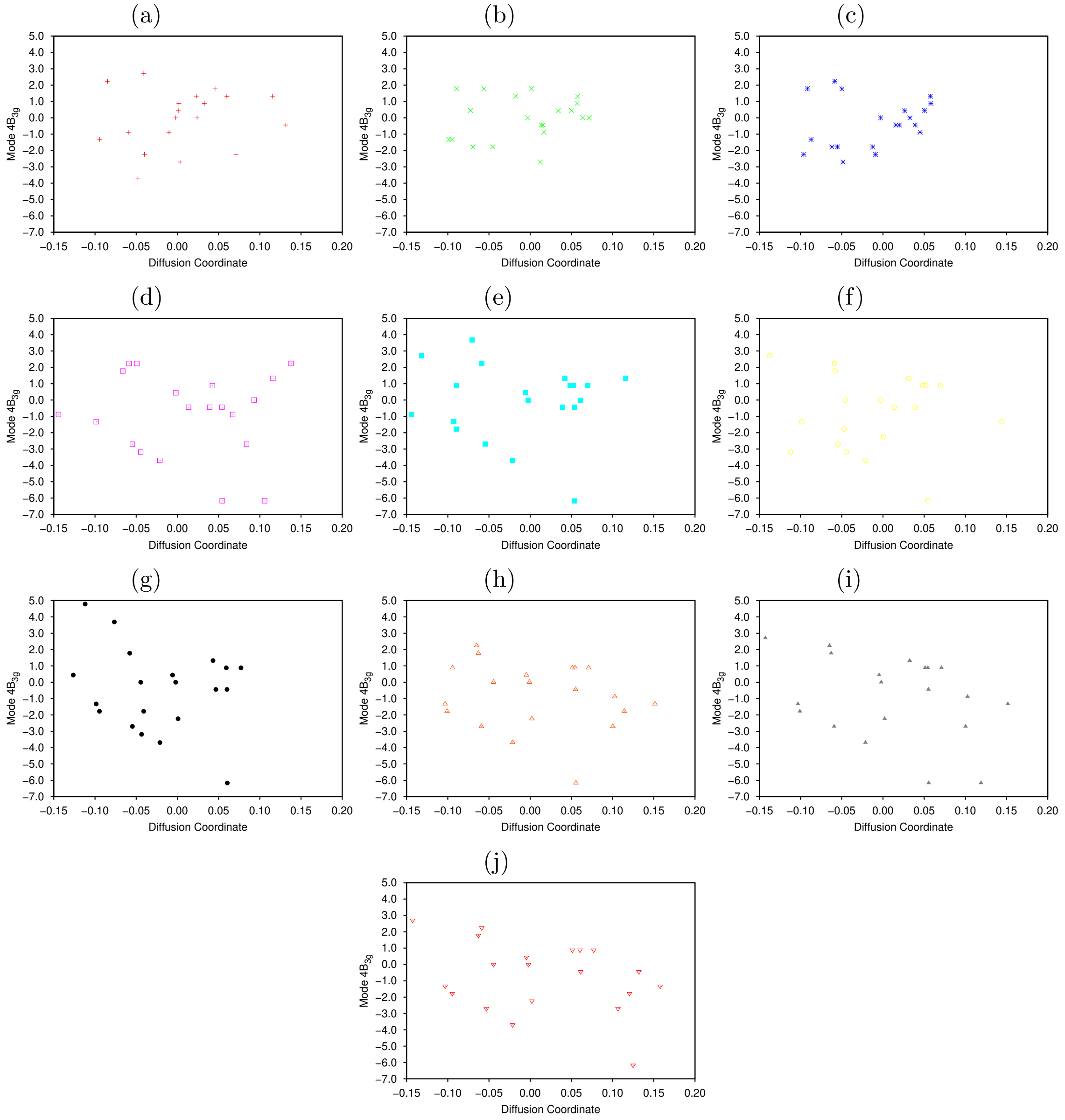

Figure S15: Sampled $4B_{3g}$ normal mode coordinates on the ground, diabatic state of ethene plotted as functions of the diffusion coordinate, with each plot representing a different sampling time: **(a)** 10 fs; **(b)** 20 fs; **(c)** 30 fs; **(d)** 40 fs; **(e)** 50 fs; **(f)** 60 fs; **(g)** 70 fs; **(h)** 80 fs; **(i)** 90 fs; **(j)** 100 fs. Data is the same as that presented in Figure 5(d) in the main paper for the 8D calculation.

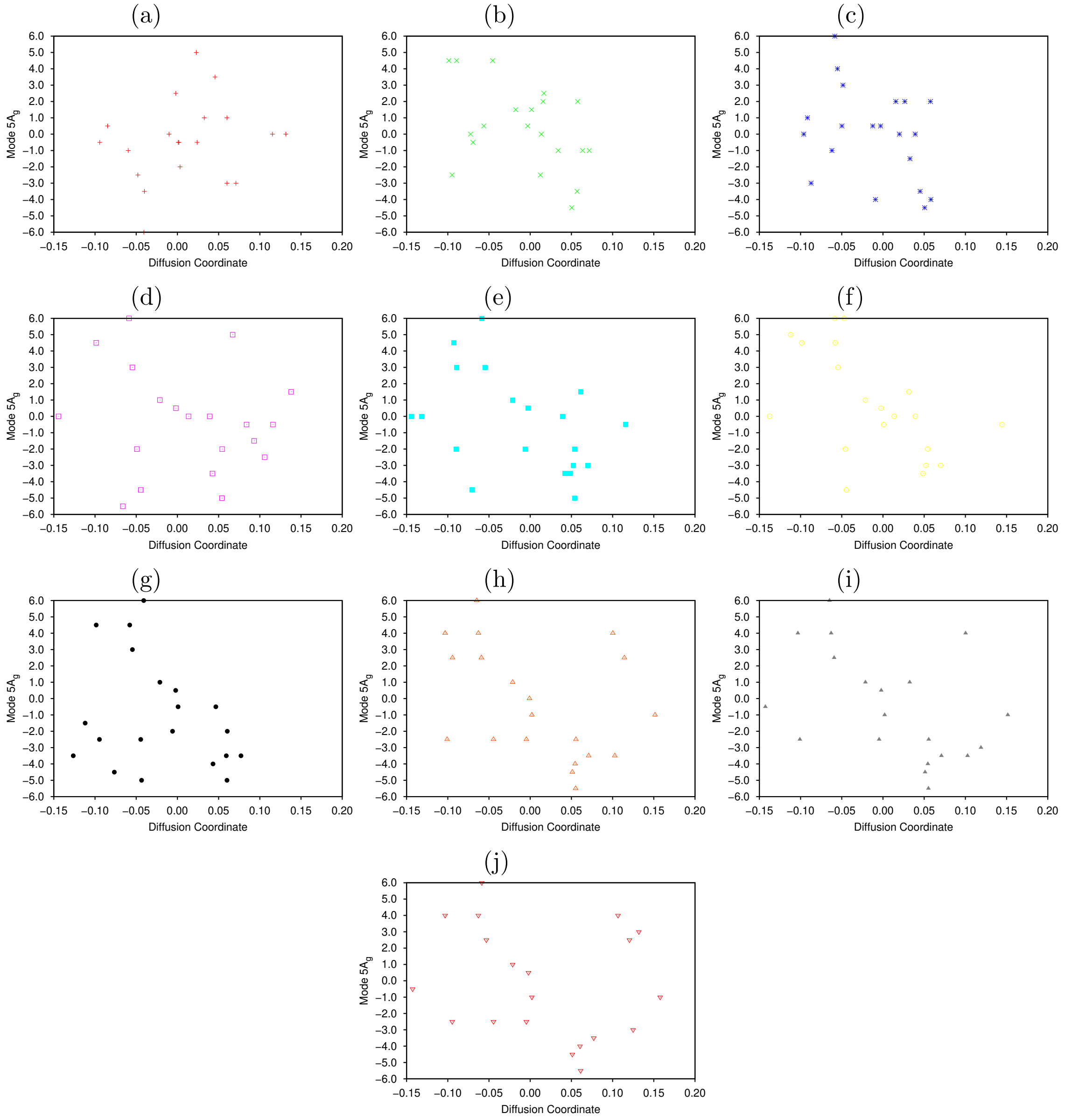

Figure S16: Sampled 5A$_g$ normal mode coordinates on the ground, diabatic state of ethene plotted as functions of the diffusion coordinate, with each plot representing a different sampling time: **(a)** 10 fs; **(b)** 20 fs; **(c)** 30 fs; **(d)** 40 fs; **(e)** 50 fs; **(f)** 60 fs; **(g)** 70 fs; **(h)** 80 fs; **(i)** 90 fs; **(j)** 100 fs. Data is the same as that presented in Figure 5(e) in the main paper for the 8D calculation.

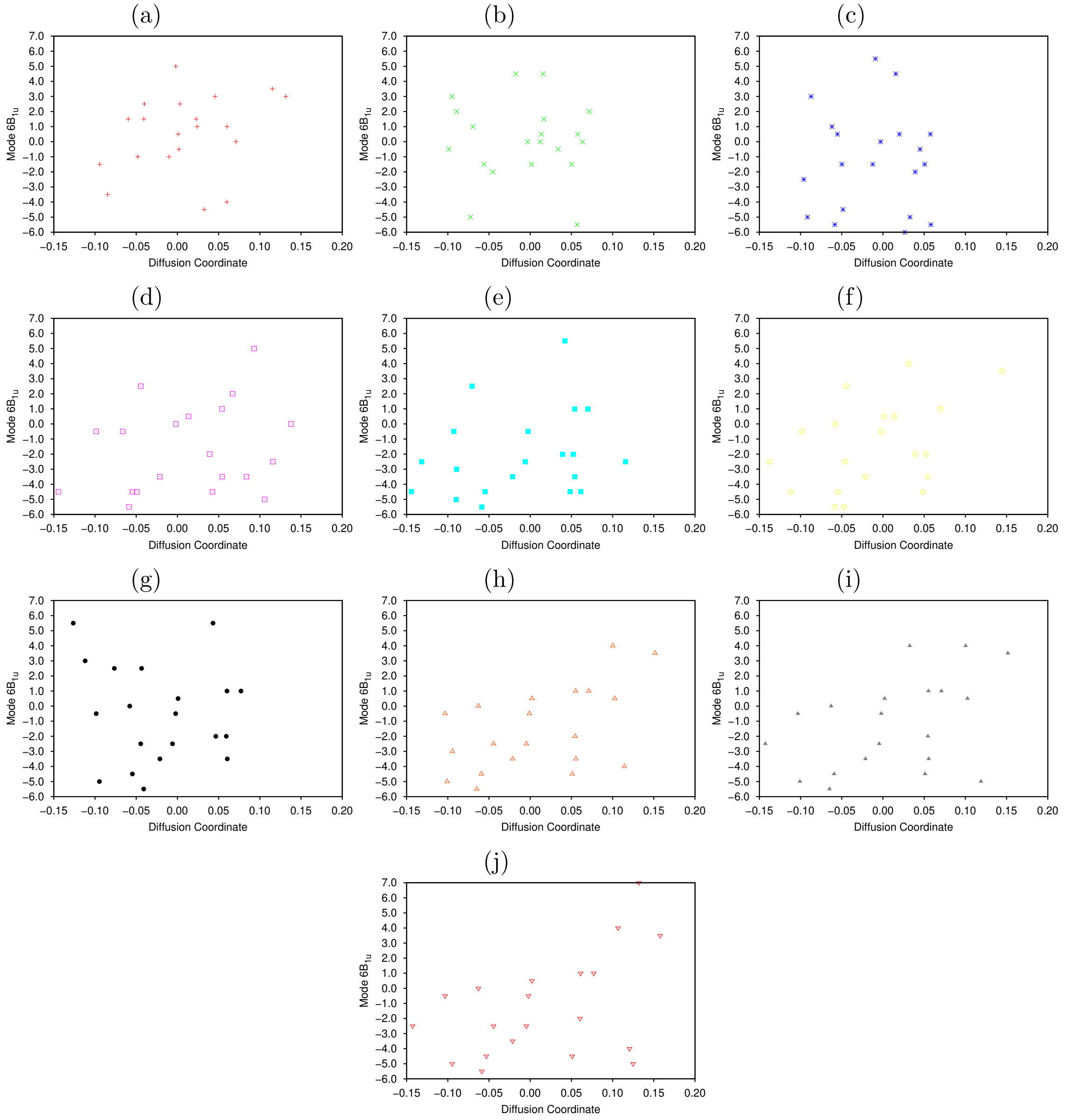

Figure S17: Sampled $6B_{1u}$ normal mode coordinates on the ground, diabatic state of ethene plotted as functions of the diffusion coordinate, with each plot representing a different sampling time: **(a)** 10 fs; **(b)** 20 fs; **(c)** 30 fs; **(d)** 40 fs; **(e)** 50 fs; **(f)** 60 fs; **(g)** 70 fs; **(h)** 80 fs; **(i)** 90 fs; **(j)** 100 fs. Data is the same as that presented in Figure 5(f) in the main paper for the 8D calculation.

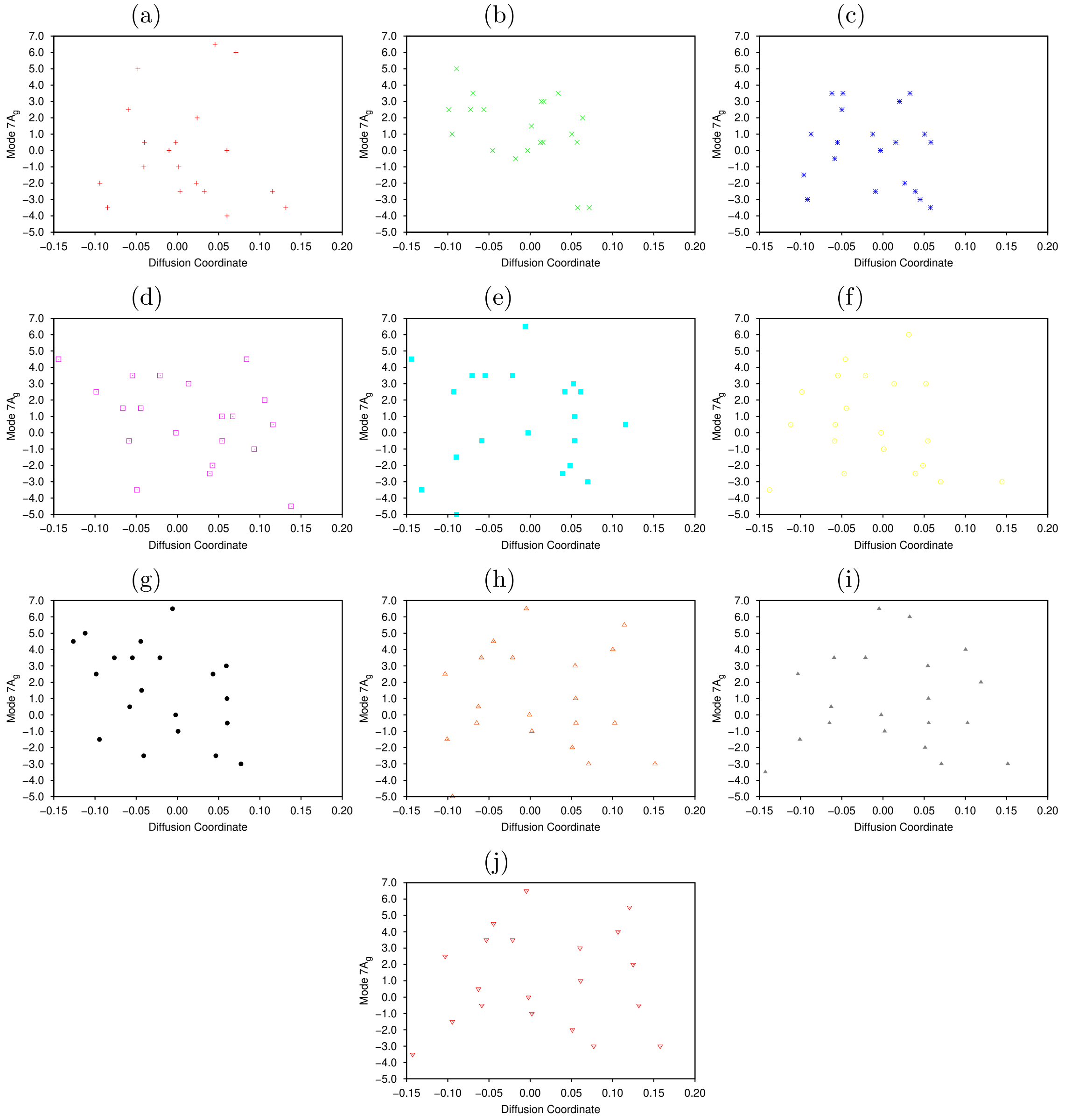

Figure S18: Sampled $7A_g$ normal mode coordinates on the ground, diabatic state of ethene plotted as functions of the diffusion coordinate, with each plot representing a different sampling time: **(a)** 10 fs; **(b)** 20 fs; **(c)** 30 fs; **(d)** 40 fs; **(e)** 50 fs; **(f)** 60 fs; **(g)** 70 fs; **(h)** 80 fs; **(i)** 90 fs; **(j)** 100 fs. Data is the same as that presented in Figure 5(g) in the main paper for the 8D calculation.

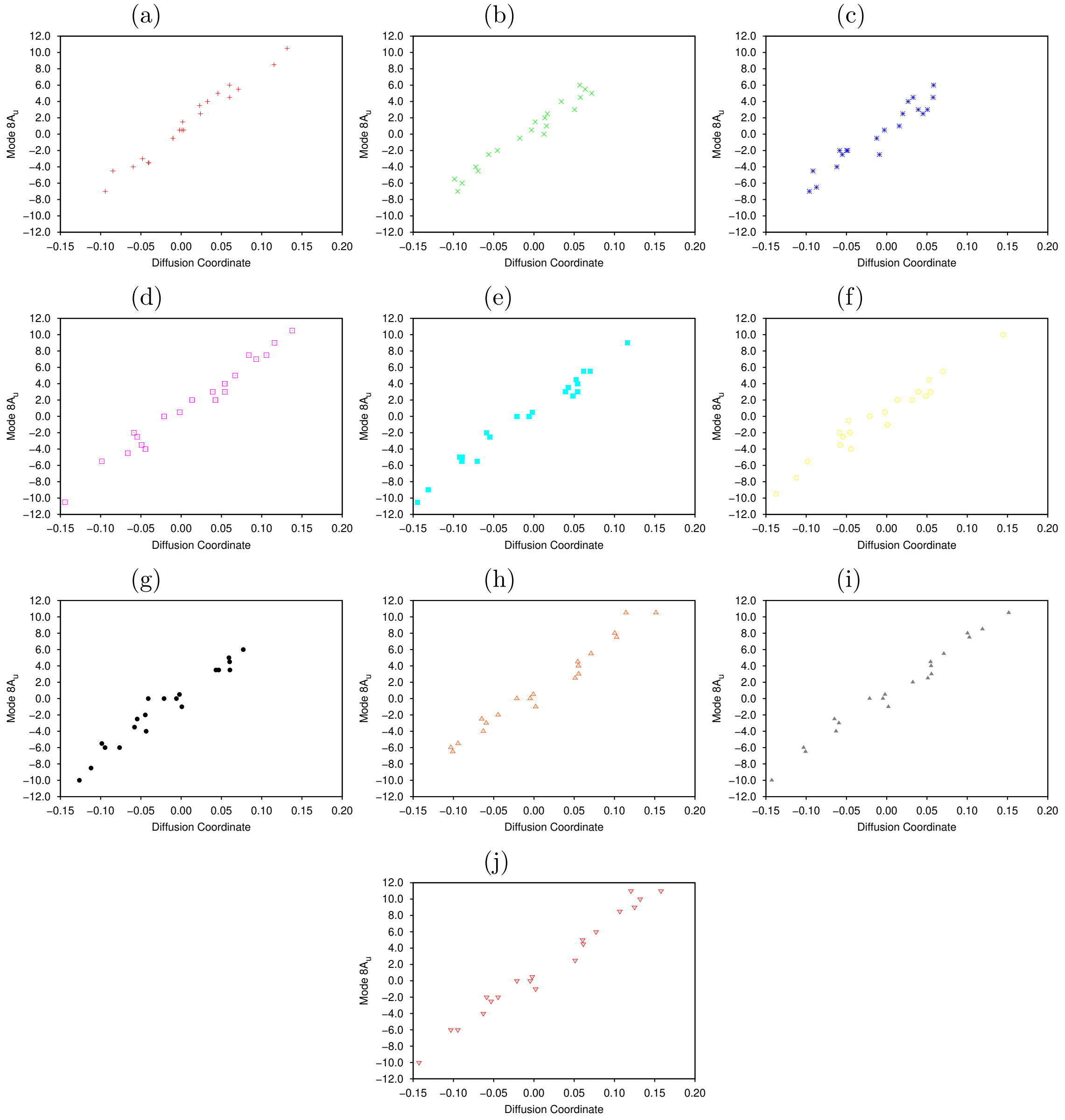

Figure S19: Sampled $8A_u$ normal mode coordinates on the ground, diabatic state of ethene plotted as functions of the diffusion coordinate, with each plot representing a different sampling time: **(a)** 10 fs; **(b)** 20 fs; **(c)** 30 fs; **(d)** 40 fs; **(e)** 50 fs; **(f)** 60 fs; **(g)** 70 fs; **(h)** 80 fs; **(i)** 90 fs; **(j)** 100 fs. Data is the same as that presented in Figure 5(h) in the main paper for the 8D calculation.

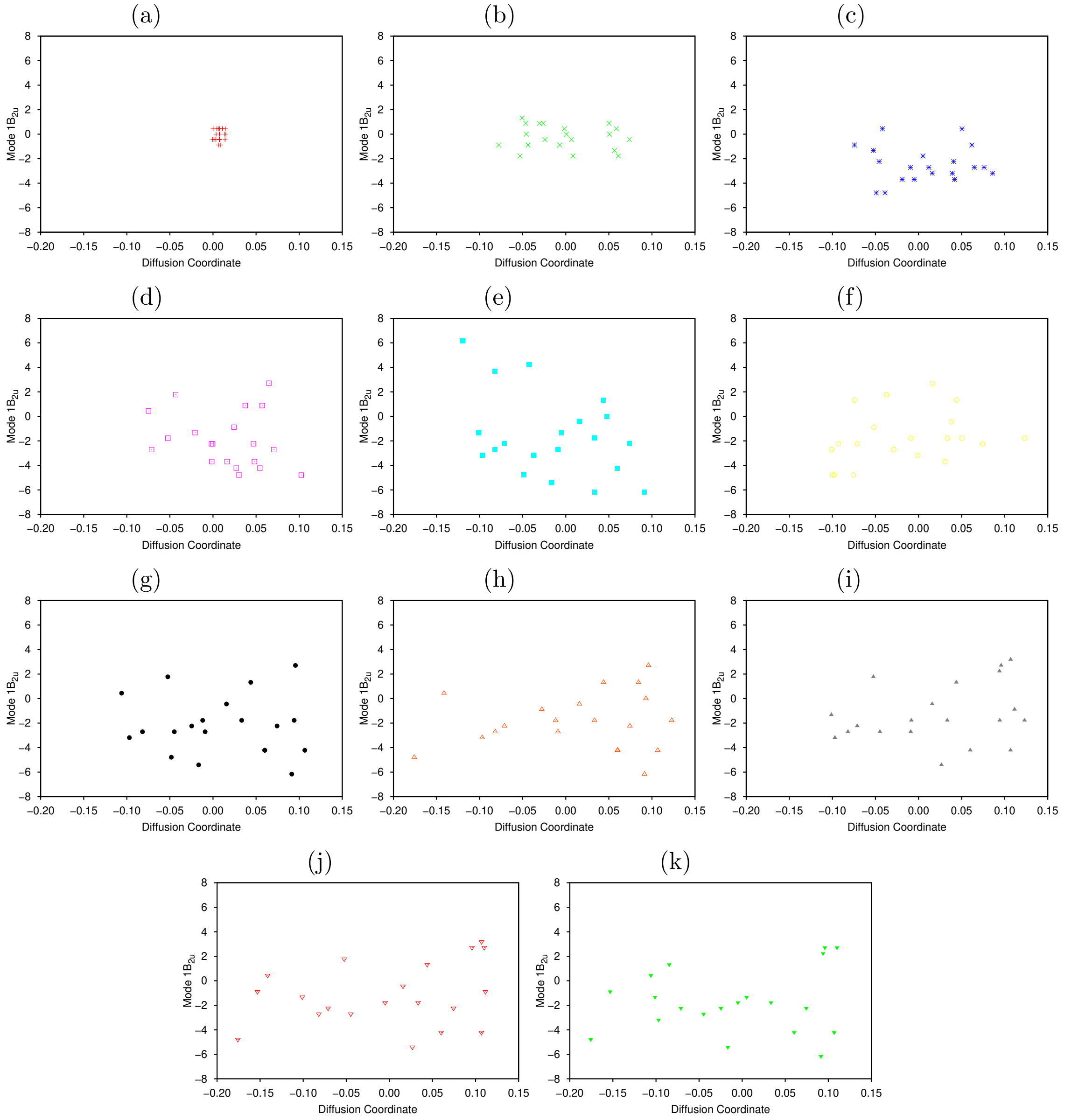

Figure S20: Sampled $1B_{2u}$ normal mode coordinates on the first, diabatic excited state of ethene plotted as functions of the diffusion coordinate, with each plot representing a different sampling time: **(a)** 0 fs; **(b)** 10 fs; **(c)** 20 fs; **(d)** 30 fs; **(e)** 40 fs; **(f)** 50 fs; **(g)** 60 fs; **(h)** 70 fs; **(i)** 80 fs; **(j)** 90 fs; **(k)** 100 fs. Data is the same as that presented in Figure 6(a) in the main paper for the 8D calculation.

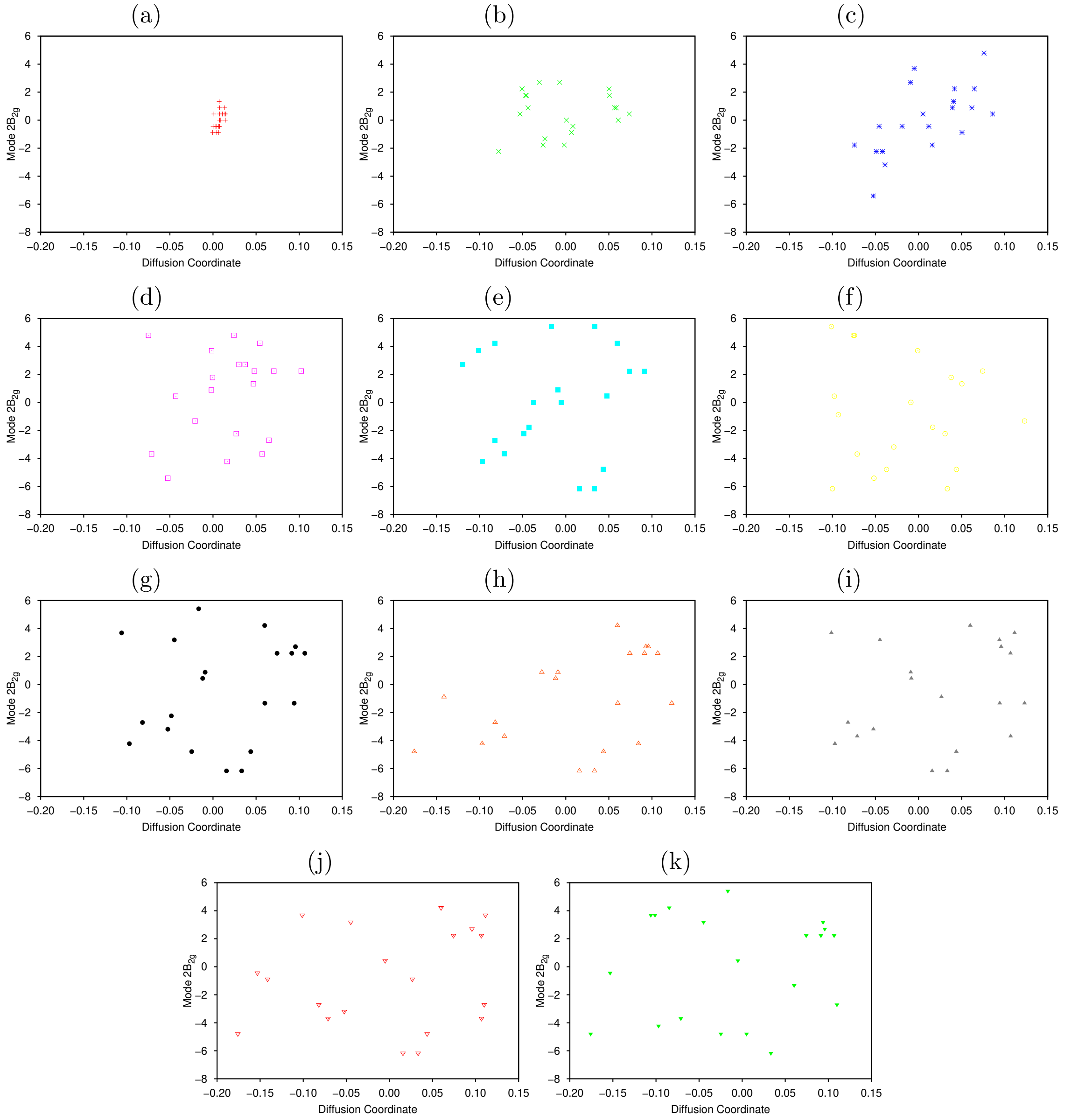

Figure S21: Sampled $2B_{2g}$ normal mode coordinates on the first, diabatic excited state of ethene plotted as functions of the diffusion coordinate, with each plot representing a different sampling time: **(a)** 0 fs; **(b)** 10 fs; **(c)** 20 fs; **(d)** 30 fs; **(e)** 40 fs; **(f)** 50 fs; **(g)** 60 fs; **(h)** 70 fs; **(i)** 80 fs; **(j)** 90 fs; **(k)** 100 fs. Data is the same as that presented in Figure 6(b) in the main paper for the 8D calculation.

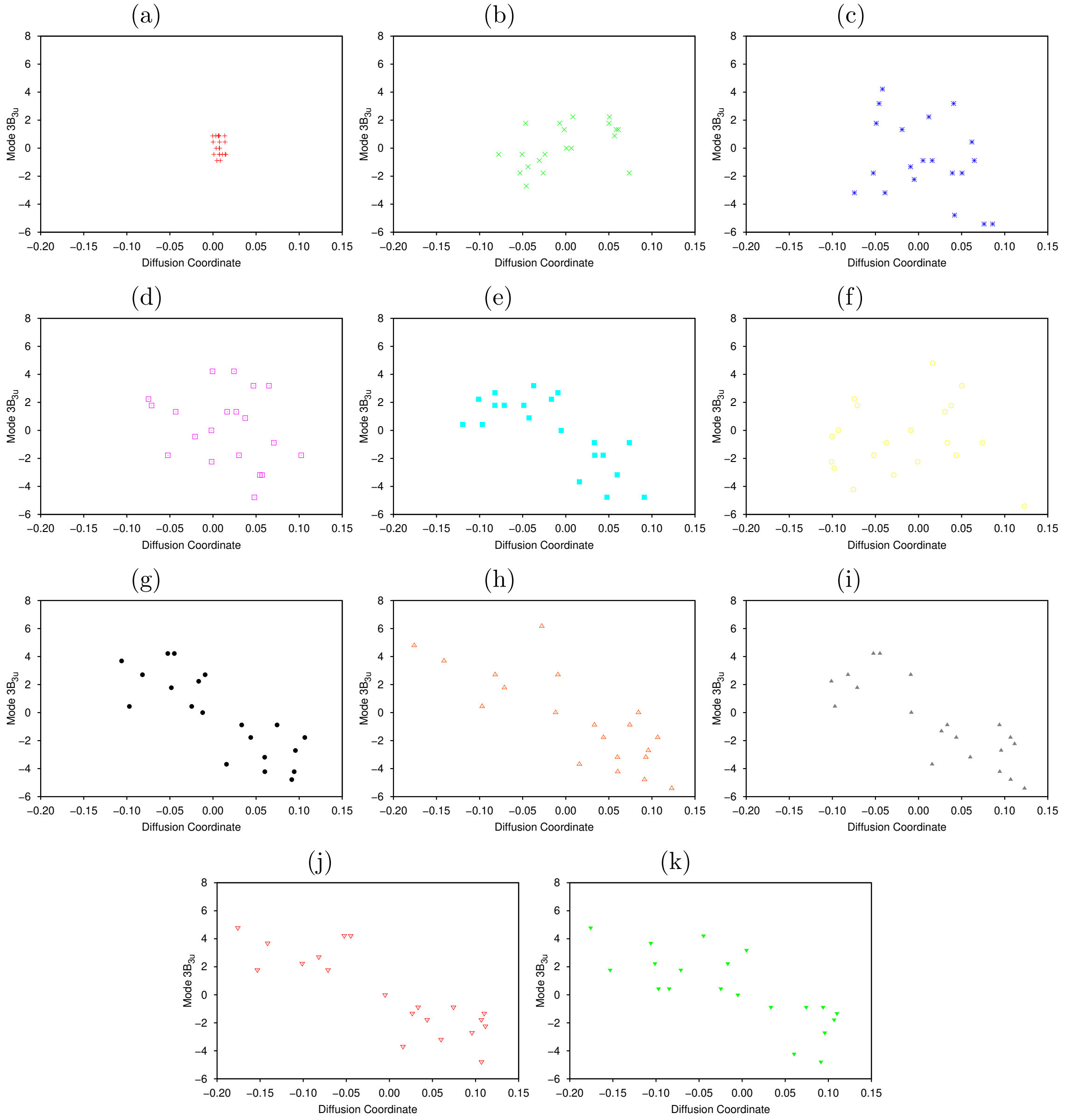

Figure S22: Sampled $3B_{3u}$ normal mode coordinates on the first, diabatic excited state of ethene plotted as functions of the diffusion coordinate, with each plot representing a different sampling time: **(a)** 0 fs; **(b)** 10 fs; **(c)** 20 fs; **(d)** 30 fs; **(e)** 40 fs; **(f)** 50 fs; **(g)** 60 fs; **(h)** 70 fs; **(i)** 80 fs; **(j)** 90 fs; **(k)** 100 fs. Data is the same as that presented in Figure 6(c) in the main paper for the 8D calculation.

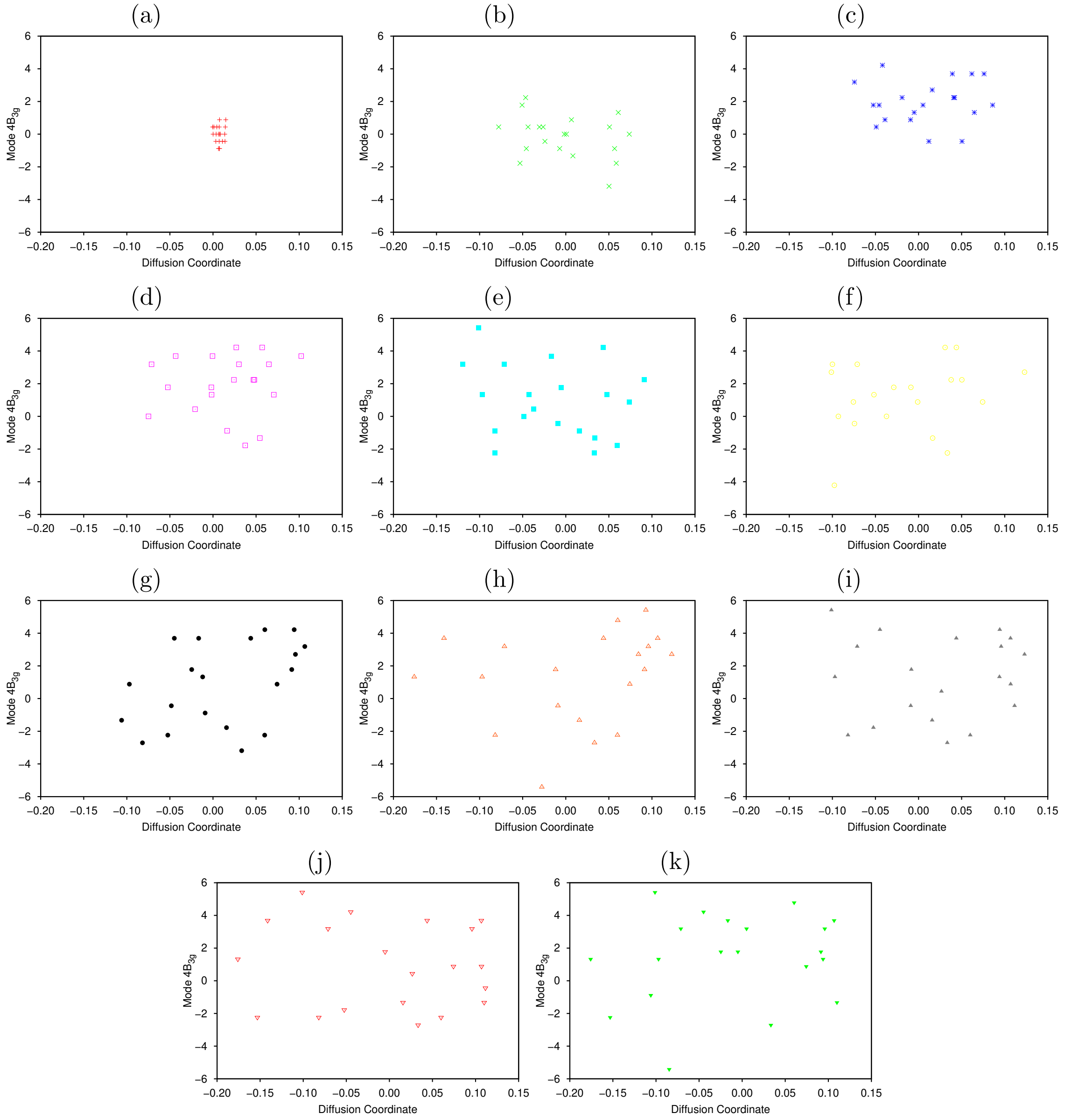

Figure S23: Sampled $4B_{3g}$ normal mode coordinates on the first, diabatic excited state of ethene plotted as functions of the diffusion coordinate, with each plot representing a different sampling time: **(a)** 0 fs; **(b)** 10 fs; **(c)** 20 fs; **(d)** 30 fs; **(e)** 40 fs; **(f)** 50 fs; **(g)** 60 fs; **(h)** 70 fs; **(i)** 80 fs; **(j)** 90 fs; **(k)** 100 fs. Data is the same as that presented in Figure 6(d) in the main paper for the 8D calculation.

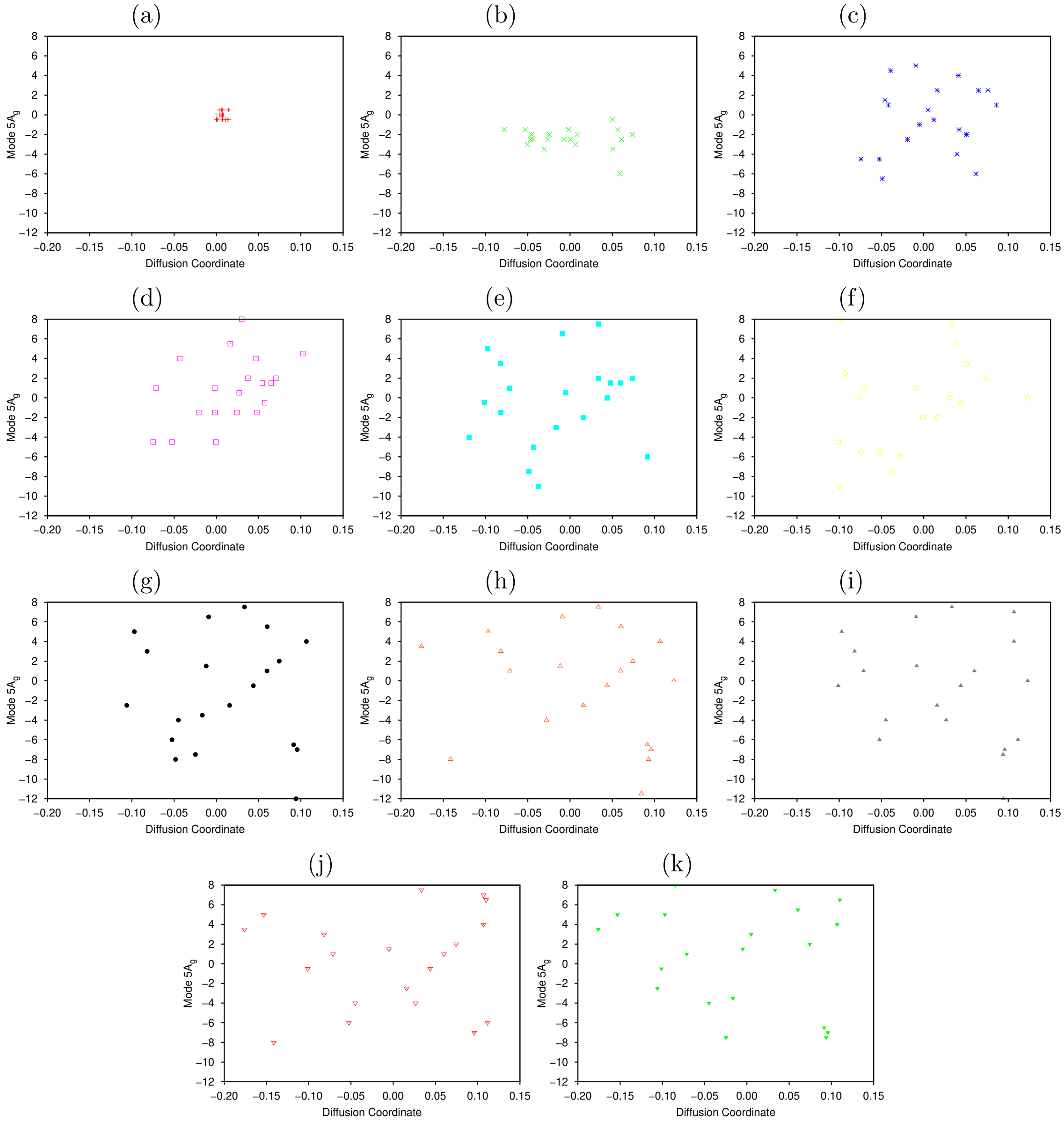

Figure S24: Sampled 5A$_g$ normal mode coordinates on the first, diabatic excited state of ethene plotted as functions of the diffusion coordinate, with each plot representing a different sampling time: **(a)** 0 fs; **(b)** 10 fs; **(c)** 20 fs; **(d)** 30 fs; **(e)** 40 fs; **(f)** 50 fs; **(g)** 60 fs; **(h)** 70 fs; **(i)** 80 fs; **(j)** 90 fs; **(k)** 100 fs. Data is the same as that presented in Figure 6(e) in the main paper for the 8D calculation.

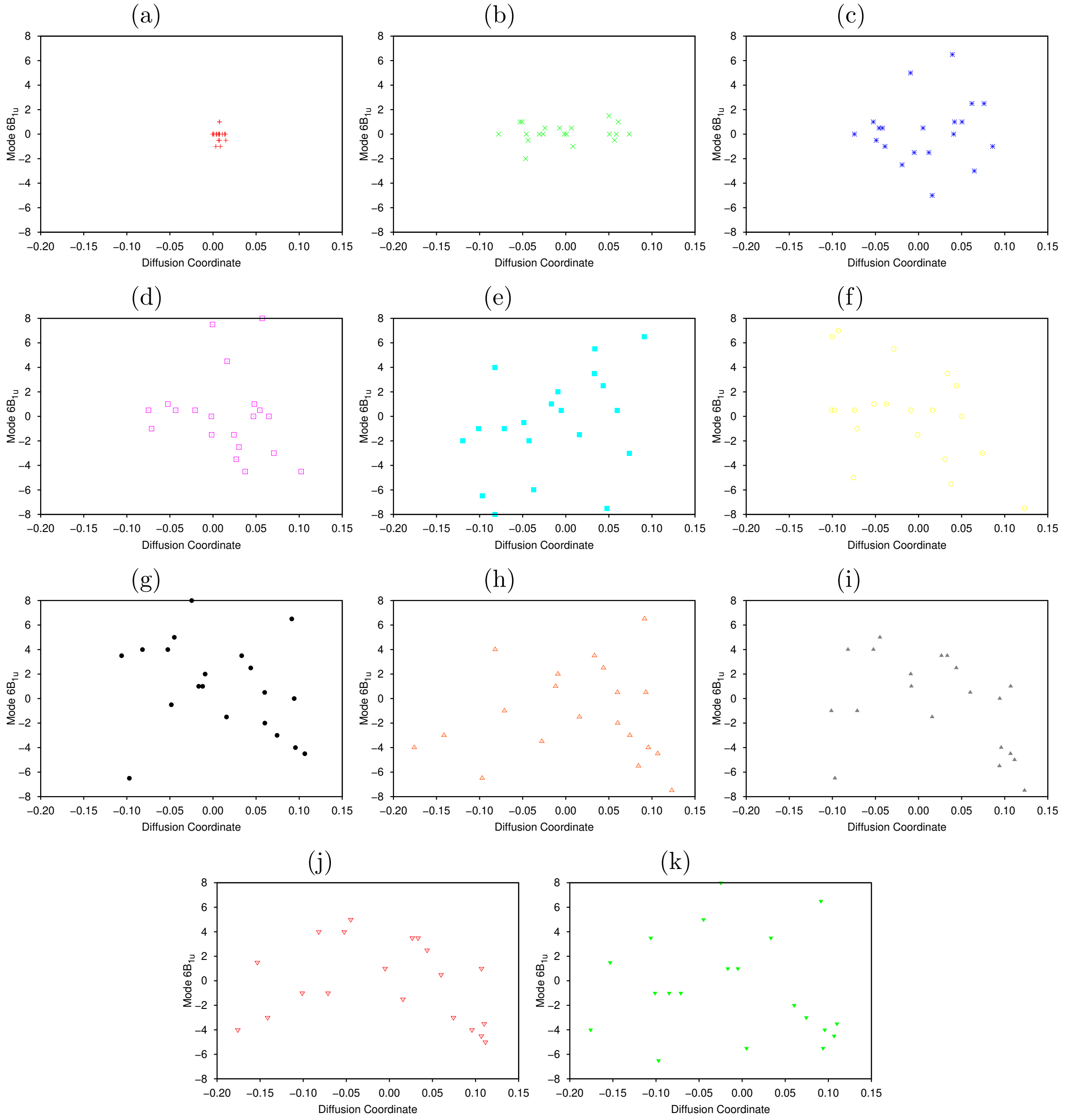

Figure S25: Sampled $6B_{1u}$ normal mode coordinates on the first, diabatic excited state of ethene plotted as functions of the diffusion coordinate, with each plot representing a different sampling time: **(a)** 0 fs; **(b)** 10 fs; **(c)** 20 fs; **(d)** 30 fs; **(e)** 40 fs; **(f)** 50 fs; **(g)** 60 fs; **(h)** 70 fs; **(i)** 80 fs; **(j)** 90 fs; **(k)** 100 fs. Data is the same as that presented in Figure 6(f) in the main paper for the 8D calculation.

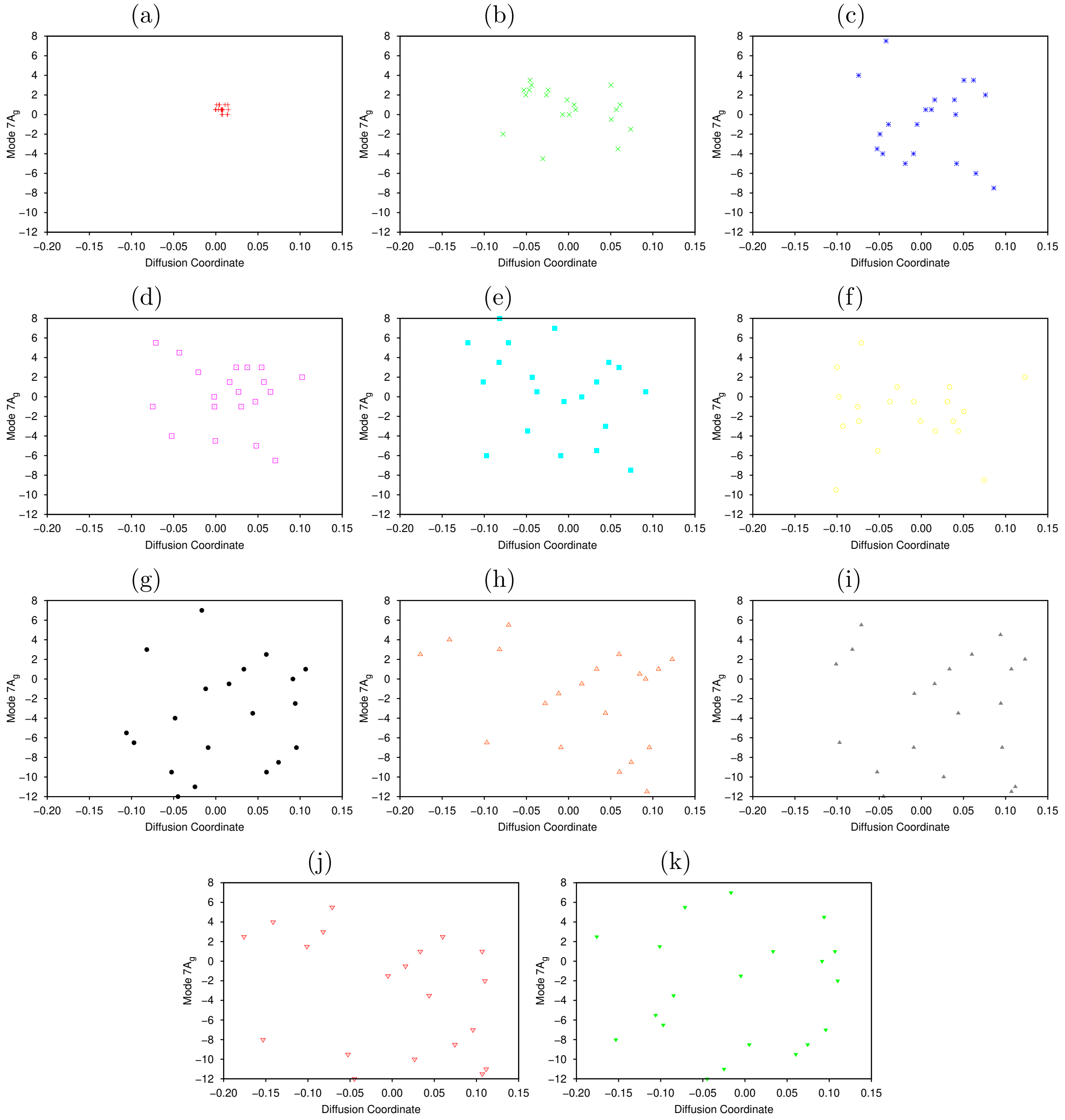

Figure S26: Sampled 7A$_g$ normal mode coordinates on the first, diabatic excited state of ethene plotted as functions of the diffusion coordinate, with each plot representing a different sampling time: **(a)** 0 fs; **(b)** 10 fs; **(c)** 20 fs; **(d)** 30 fs; **(e)** 40 fs; **(f)** 50 fs; **(g)** 60 fs; **(h)** 70 fs; **(i)** 80 fs; **(j)** 90 fs; **(k)** 100 fs. Data is the same as that presented in Figure 6(g) in the main paper for the 8D calculation.

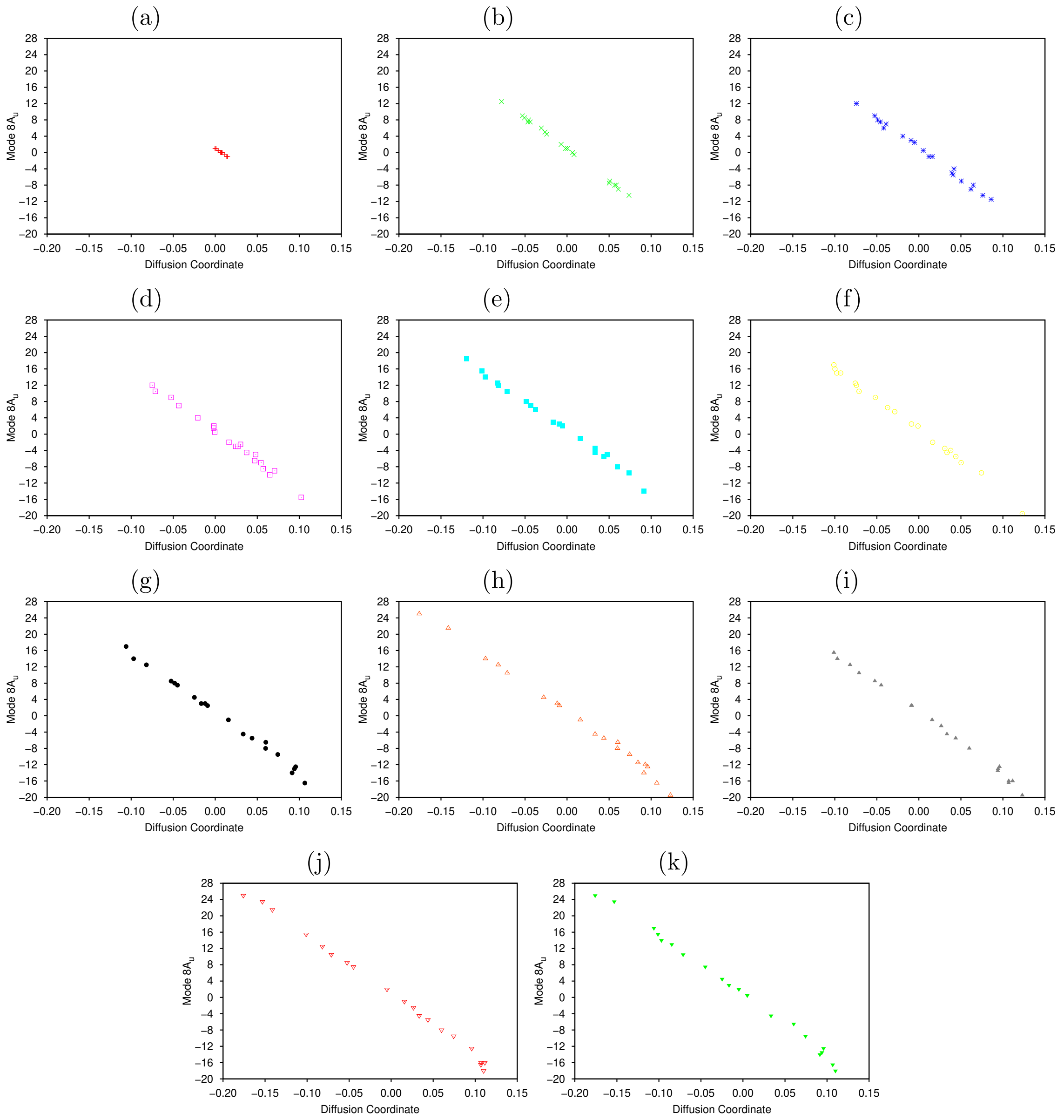

Figure S27: Sampled $8A_u$ normal mode coordinates on the first, diabatic excited state of ethene plotted as functions of the diffusion coordinate, with each plot representing a different sampling time: **(a)** 0 fs; **(b)** 10 fs; **(c)** 20 fs; **(d)** 30 fs; **(e)** 40 fs; **(f)** 50 fs; **(g)** 60 fs; **(h)** 70 fs; **(i)** 80 fs; **(j)** 90 fs; **(k)** 100 fs. Data is the same as that presented in Figure 6(h) in the main paper for the 8D calculation.

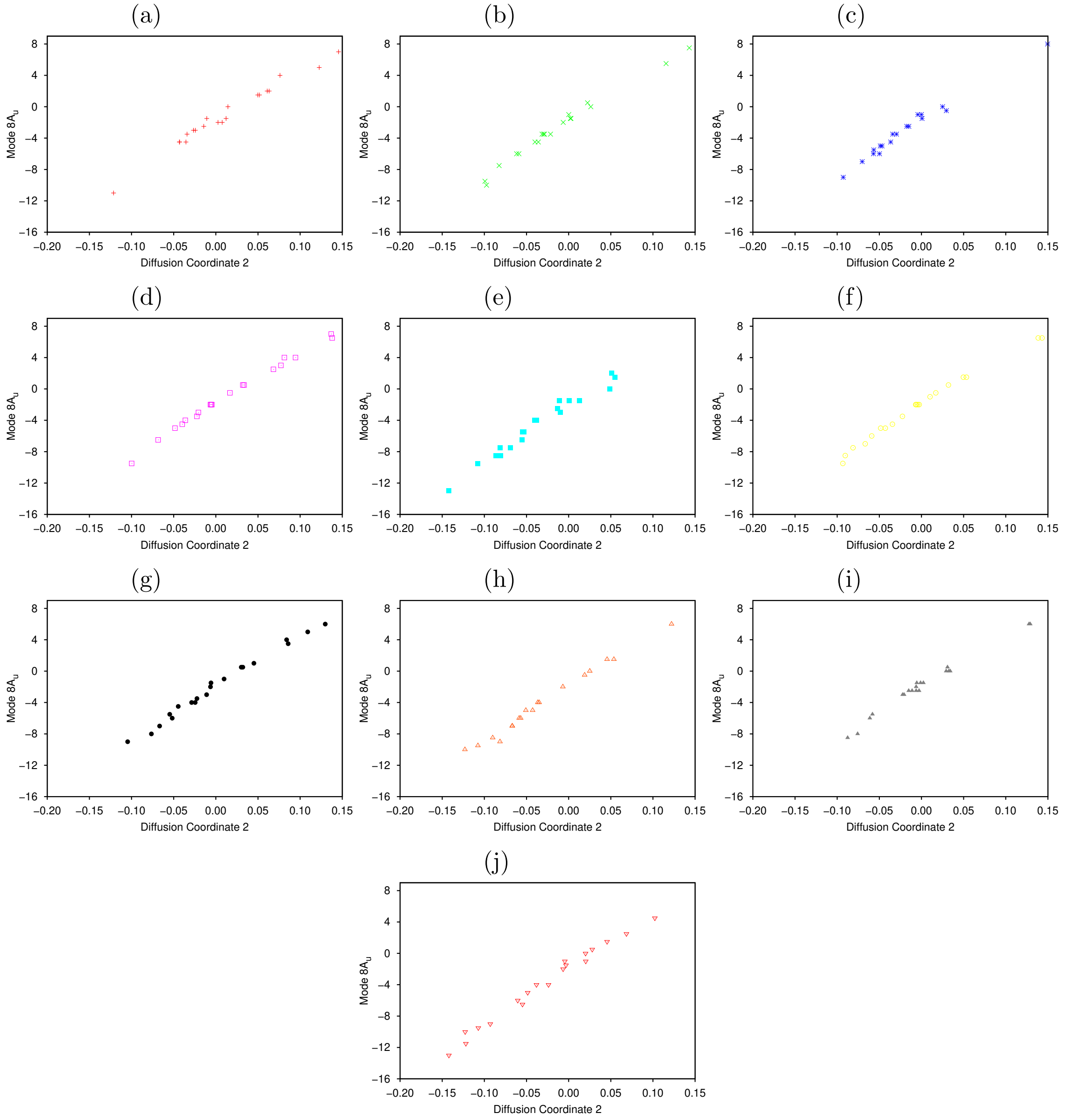

Figure S28: Sampled 8A$_u$ normal mode coordinates on the ground, diabatic state of ethene plotted as functions of diffusion coordinate 2, with each plot representing a different sampling time: **(a)** 10 fs; **(b)** 20 fs; **(c)** 30 fs; **(d)** 40 fs; **(e)** 50 fs; **(f)** 60 fs; **(g)** 70 fs; **(h)** 80 fs; **(i)** 90 fs; **(j)** 100 fs. Data is the same as that presented in Figure 8(a) in the main paper for the 12D calculation.

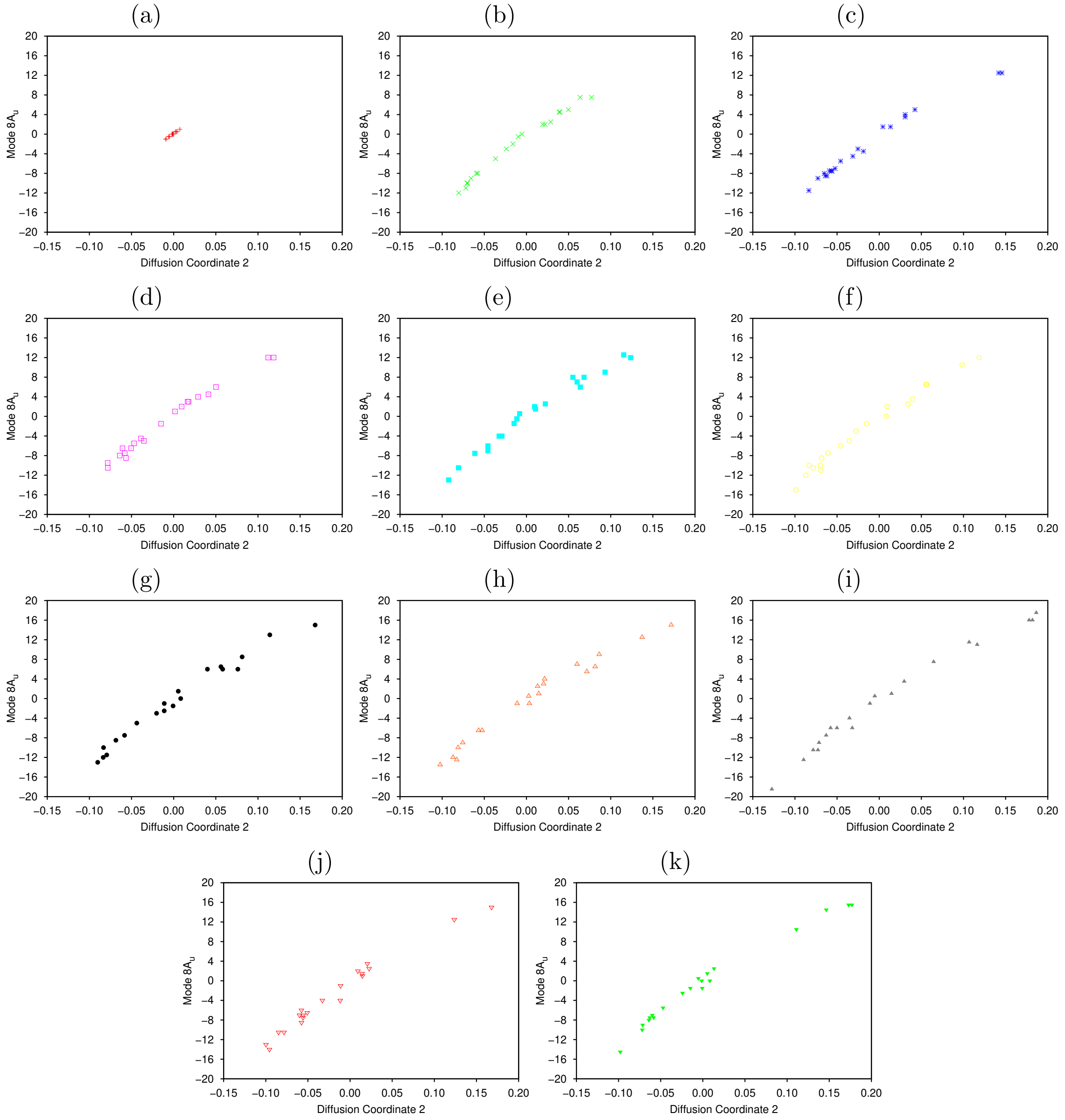

Figure S29: Sampled $8A_u$ normal mode coordinates on the first, diabatic excited state of ethene plotted as functions of diffusion coordinate 2, with each plot representing a different sampling time: **(a)** 0 fs; **(b)** 10 fs; **(c)** 20 fs; **(d)** 30 fs; **(e)** 40 fs; **(f)** 50 fs; **(g)** 60 fs; **(h)** 70 fs; **(i)** 80 fs; **(j)** 90 fs; **(k)** 100 fs. Data is the same as that presented in Figure 8(b) in the main paper for the 12D calculation.

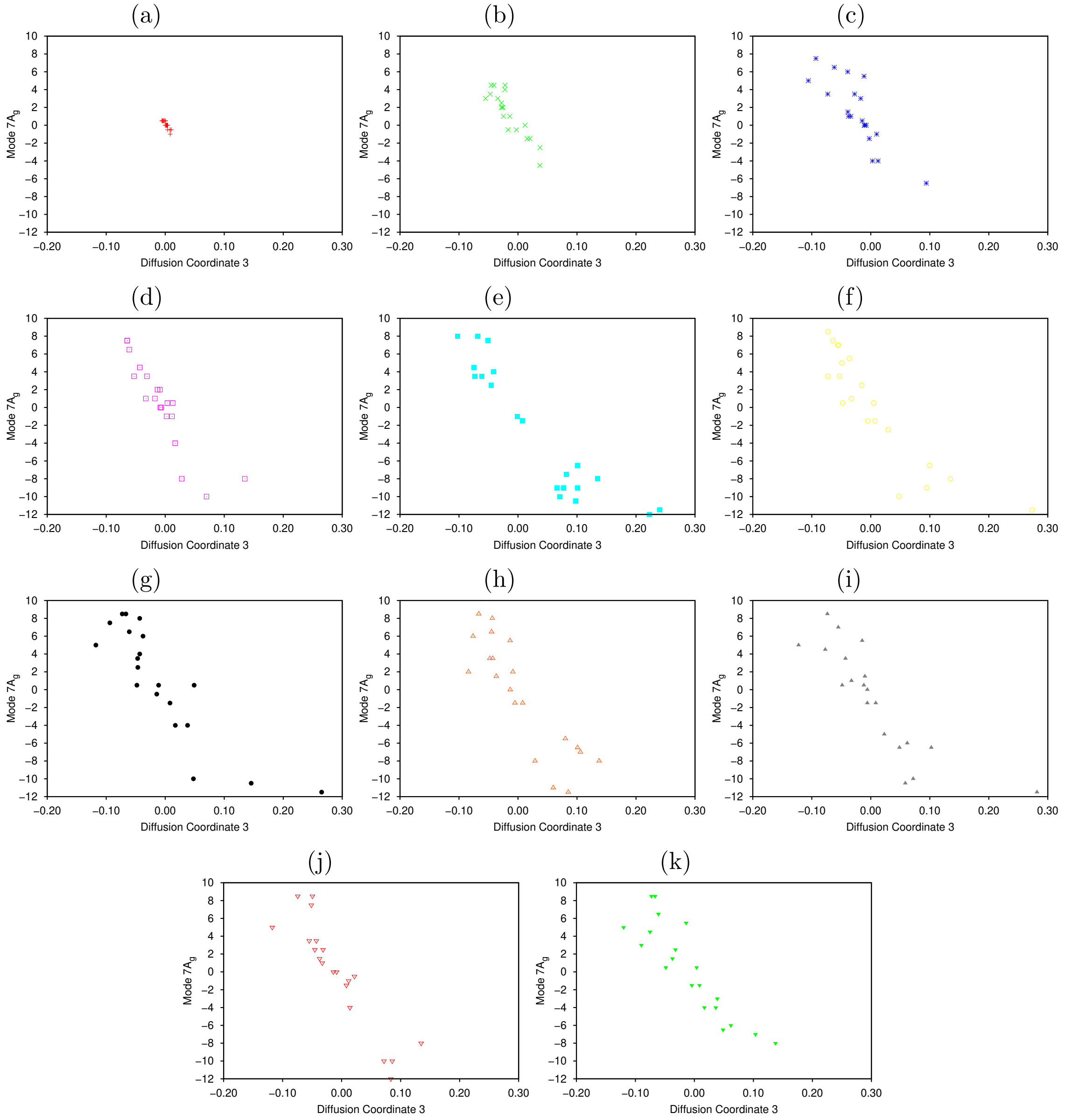

Figure S30: Sampled $7A_g$ normal mode coordinates on the first, diabatic excited state of ethene plotted as functions of diffusion coordinate 3, with each plot representing a different sampling time: **(a)** 0 fs; **(b)** 10 fs; **(c)** 20 fs; **(d)** 30 fs; **(e)** 40 fs; **(f)** 50 fs; **(g)** 60 fs; **(h)** 70 fs; **(i)** 80 fs; **(j)** 90 fs; **(k)** 100 fs. Data is the same as that presented in Figure 8(c) in the main paper for the 12D calculation.
